# Augmenting small tabular health data for training prognostic ensemble machine learning models using generative models

**DOI:** 10.1186/s12911-025-03266-3

**Published:** 2025-11-28

**Authors:** Dan Liu, Samer El Kababji, Nicholas Mitsakakis, Lisa Pilgram, Thomas D. Walters, Mark Clemons, Gregory R. Pond, Alaa El-Hussuna, Khaled El Emam

**Affiliations:** 1 Children’s Hospital of Eastern Ontario Research Institute, 401 Smyth Road, Ottawa, ON K1h 8l1 Canada; 2 School of Epidemiology and Public Health, University of Ottawa, Ottawa, ON Canada; 3 Department of Nephrology and Medical Intensive Care, Charité - Universitaetsmedizin Berlin, Berlin, Germany; 4 Hospital for Sick Children, Toronto, ON Canada; 5 Ottawa Hospital Research Institute, Ottawa, ON Canada; 6 Division of Medical Oncology, Department of Medicine, University of Ottawa, Ottawa, ON Canada; 7 McMaster University, Hamilton, ON Canada; 8 OpenSourceResearch, Aalborg, Denmark

**Keywords:** Data augmentation, Machine learning, Generative models, Artificial intelligence, Data scarcity, Synthetic data

## Abstract

**Background:**

Small datasets are common in health research. However, the generalization performance of machine learning models is suboptimal when the training datasets are small. To address this, data augmentation is one solution and is often used for imaging and time series data, but there are no evaluations on its potential benefits for tabular health data. Augmentation increases sample size and is seen as a form of regularization that increases the diversity of small datasets, leading them to perform better on unseen data.

**Objectives:**

Evaluate data augmentation using generative models on tabular health data and assess the impact of diversity versus increasing the sample size.

**Methods:**

Using 13 large health datasets, we performed a simulation to evaluate the impact of data augmentation on the prediction performance (as measured by the ROC-AUC, the area under the receiver operating characteristic curve) on binary classification gradient boosted decision tree models. Four different synthetic data generation models were evaluated. We also built a generalized linear mixed effect model to assess the variable importance for model performance improvements from augmentation. We illustrate the proposed method on seven small real datasets as an application. A comparison of augmentation with resampling (which is a proxy for a larger dataset with minimal impact on diversity) was performed.

**Results:**

Augmentation improves prognostic performance for datasets that have higher cardinality categorical variables and lower baseline ROC-AUC. No specific generative model consistently outperformed the others. For the seven small application datasets, augmenting the existing data results in an increase in ROC-AUC between 4.31% (ROC-AUC from 0.71 to 0.75) and 43.23% (ROC-AUC from 0.51 to 0.73), with an average 15.55% relative improvement, demonstrating the nontrivial impact of augmentation on small datasets (*p* = 0.0078). Augmentation ROC-AUC was higher than resampling only ROC-AUC (*p* = 0.016). The diversity of augmented datasets was higher than the diversity of resampled datasets (*p* = 0.046).

**Conclusions:**

This study demonstrates that data augmentation using generative models can have a marked benefit in terms of improved predictive performance for machine learning models on tabular health data, but only for datasets that meet baseline data complexity and predictive performance criteria. Our mixed effect model identified the most influential characteristics of the dataset and can help end-users have a more realistic expectation of the augmentation performance for a new dataset. Furthermore, augmentation performed better when having a smaller dataset, which is consistent with the argument that greater data diversity due to augmentation is beneficial.

**Clinical trial registration:**

Not applicable.

**Supplementary information:**

The online version contains supplementary material available at 10.1186/s12911-025-03266-3.

## Introduction

Many machine learning (ML) clinical prediction models are trained on datasets that are too small. Specifically, a median of 12.5 events per predictor variable has been reported in the literature [1] and 1.7 for oncology ensemble models [2]. However, to achieve stability while training ML models more than 200 events per predictor variable are often required [3], and the vast majority of ML modeling studies in oncology did not meet the minimum recommended sample sizes [4].

To address this data scarcity problem, there is a growing interest in using data augmentation to simulate additional observations from existing data [5]. This augmentation process increases the sample size of the dataset, which by itself is expected to improve ML model prognostic performance [3]. Augmentation can also be seen as a form of regularization [6], where the simulated data increase the diversity of the original dataset by generating more and different examples from the same population. Therefore, augmentation could improve the prediction accuracy on the unseen data and enable ML models trained on augmented data to achieve better generalization performance.

Despite the encouraging results on augmentation for different data modalities, only a small number of studies have been conducted to evaluate the impact of augmentation on tabular health data, and there is a lack of clear understanding of how and to what extent data augmentation affects the ML prognostic model performance on tabular data. To fill this gap, we make two contributions represented as two parts of the study in the current paper. First, we examine the data characteristics that impact augmentation for predictive modeling workloads. Second, we evaluate the extent to which the augmentation benefit is driven by data diversity over simply increasing the sample size.

The results that we obtained from extensive experiments demonstrate the benefits of data augmentation on tabular health data, particularly on those that have lower baseline ROC-AUC and categorical variables with higher cardinality. In the case studies, at least 4% of improvements have been observed in predictive model performance. We also provide evidence that the benefit of augmentation is due to the augmented data being more diversified instead of due to just adding more observations. These results support the wider use of generative model supported data augmentation for tabular data as a means to improve predictive performance for ML models trained on small datasets.

The rest of the paper is organized as follows. Section “Related work” discusses the existing relevant work. Section “Methods” presents our entire framework and experimental scheme designed to evaluate the augmentation approach using several generative models. Experimental and case studies results are presented in Section “Results”. Finally, Section “Discussion” includes our discussions, conclusions and recommendations for the practical use of our proposed approach.

## Related work

Data augmentation has been used in multiple domains, such as imaging, video and natural language processing data [5, 7–11], as summarized below.

Synthetic imaging samples can boost model accuracy [12–20]. Moreover, generative models, such as generative adversarial networks (GAN) and CycleGANs, were found particularly useful to transfer relatively abundant data modalities (e.g., CT, MRI) to either more expensive or underrepresented modalities [21–24]. Recently, several diffusion generative models achieved competitive results compared to GANs, while possessing better distribution coverage and ease of training and scalability [25–28].

In addition, it is worth noting that despite the wide application of augmentation techniques for imaging data, many are not directly applicable to tabular data. For example, flipping and cropping is a commonly used imaging augmentation technique to increase the diversity and expand the available data [7, 29, 30]. However, the rows and columns in the tabular data are often correlated, and either horizontal or vertical flipping may harm the data semantics and distort the underlying relationships. Color jitter is another powerful augmentation tool for imaging data that randomly adjusts the color channels and image brightness of the image without changing the object identity [31, 32]. However, when applying this to tabular data, such random variations may potentially disrupt the feature distributions and data structure.

Augmentation has also been applied to time series datasets, which, unlike imaging data, are typically difficult to access and obtain [33]. Data augmentation has been shown to be a viable solution to address the problem of incomplete and unbalanced time series datasets [29, 34–36].

Additionally, text classification is another domain of application. For instance, data augmentation enhanced the performance of learners on the confusion set disambiguation problem in text classification, compared to the choice of classifier [37]. Generic data augmentation was used to generate more adversarial examples for the text data in order to improve the deep learning model performance and reduce the impact of small changes in the text on class prediction [12, 38–41].

Data augmentation has been used to deal with small clinical trial data [42, 43]. Virtual subjects were successfully generated through deep learning models such as GANs and tabular variational autoencoders (VAEs), and the quality of synthetic clinical trial data were further improved using an interpretable data augmentation framework when the original data were limited [44–46].

Another popular application of augmentation in the literature is for gene expression data, which are high-dimensional, small in size and costly to gather [47]. Several variants of the traditional GAN models were introduced to generate high-quality synthetic genomic data and augment the original small gene expression data to enhance prediction performance [47–50]. Moreover, VAE models, though comparatively underexplored, were extended and demonstrated the potential benefits of augmentation techniques for genomic data [17, 51].

Tabular data are ubiquitous in practice, particularly in the health domain [52]. However, augmentation methods are typically applied in the case of outcome class imbalance. Variants of deep learning-based techniques, such as the conditional GAN and conditional Wasserstein GAN with gradient penalty, have been specifically presented as powerful augmentation tools to alleviate the class imbalance problem for metabolomics datasets [49, 53, 54]. When there is covariate imbalance, with certain groups under-represented in the data, generative models have been used to mitigate the representation bias that is introduced [55]. For augmenting overall records, methods such as sampling with replacement, sequential synthesis using decision trees [56], GANs [57], and VAEs [58] have been evaluated with encouraging results [59–63], though some deep learning architectures were found to be unstable [64].

Notwithstanding the promising findings on structured data, evaluations thus far have been small scale, and there is limited evidence on why augmentation works on tabular data. As a result, we explore several commonly used generative models for tabular data augmentation to assess the extent of benefit and gain an understanding of the data characteristics that boost the predictive model performances.

## Methods

Our study consists of a large scale simulation and evaluation of the extent to which augmentation can improve the predictive performance of gradient boosted decision trees (GBDTs) [65], and an examination of the factors that influence that performance benefit.

### Overview of simulation and evaluation processes

**Fig. 1:**
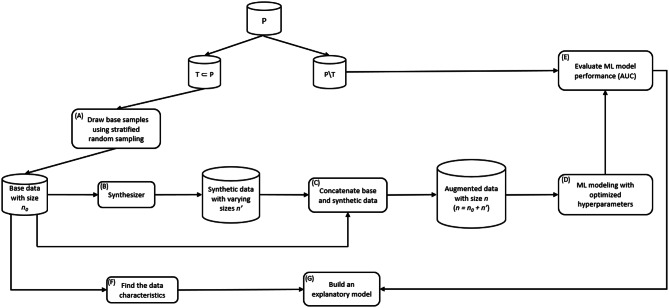
The methods workflow for part 1 of the study

**Fig. 2:**
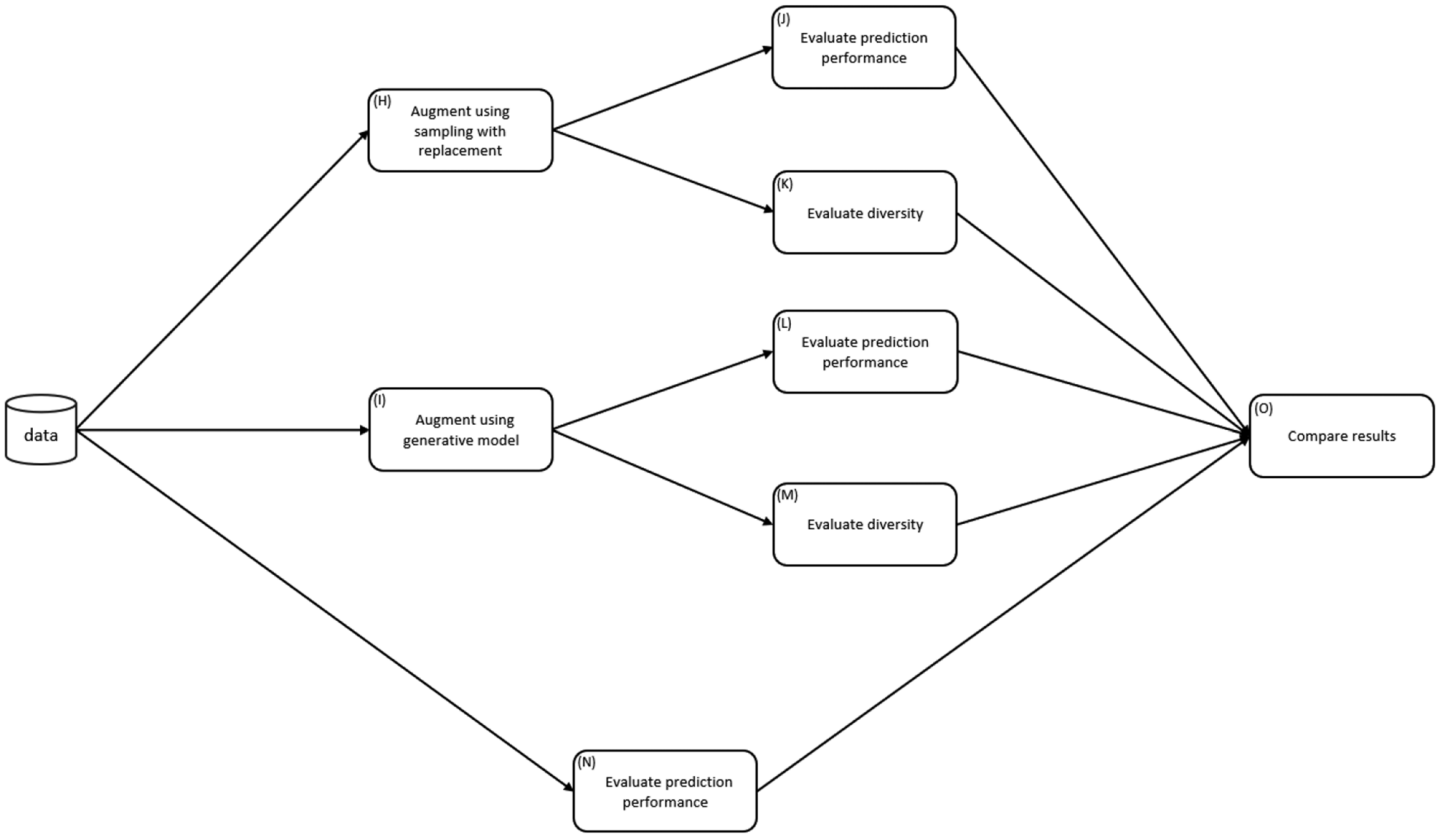
The methods workflow for part 2 of the study

The study had two parts. The overall workflow for part 1 is shown in Fig. 1, and part 2 in Fig. 2.

For part 1, we began with a large population dataset P and randomly split it into a training dataset T and a test dataset P\T with a 70%:30% split for train:test. The test set represents unseen patients that we used to evaluate the augmented data on.

We then drew a simple random sample (step A) of size n_0_ from the training dataset (the *base* dataset), which was augmented using a generative model, also called a synthesizer (step B), with a set of additional n’ records. The augmented dataset of size *n* = n_0_ + n’ (step C) was used to train a binary GBDT model (step D). The performance of that trained model was evaluated on the test dataset using the area under the receiver operating characteristic curve (ROC-AUC) (step E). This process was repeated for multiple values of n_0_.

We then trained a generalized linear mixed effect model using all the data generated from these simulations (step G) as well as specific characteristics of each dataset (step F) to evaluate the impacts of data characteristics and determine the ones that have the most impact on the predictive model performance.

Part 2 of the study involved the application of the augmentation using seven new datasets with realistic sizes seen in clinical research. In all cases, the generative models were used to augment the datasets (step I). Then, we evaluated the predictive performance improvement that one can expect to see through augmentation (comparing the results from L vs N).

We took the same seven datasets and augmented them using sampling with replacement (step H). This was intended to increase the sample size but not impact data diversity (comparing the results from K vs. M). By comparing the predictive performance of GBDT on augmented datasets with elevated diversity to that of resampled datasets with minimal impact on diversity, we were able to determine whether benefits from augmentation are due to diversity or due to larger sample sizes (comparing the results from J vs L).

In the remainder of the methods section, we provide details on these steps.

### Datasets

We have two sets of data corresponding to the two parts of our study.

The population real world datasets that were used in part 1 of the study are summarized in Table 1. These datasets cover heterogeneous domains, including public health, hospital discharge, infant and maternal health, adverse events, ICU, population health surveys and insurance claims. The table provides an overview of the datasets, the original number of observations, the number of observations after removing those with any missing values in the outcome variable and the number of variables included in the binary classification models used to predict the outcome. A detailed description of each preprocessed dataset and the binary workload used for modeling can be found in Appendix. The number of predictor variables in the workloads is consistent with what is seen in the clinical research literature [1].

**Table 1:** A description of the thirteen datasets used in the first simulation part of the study

Dataset	Description	No. observations (original)	**No. observations**	No. Variables used in the analysis
Better Outcomes Registry & Network (BORN)	A population registry containing comprehensive perinatal, newborn and child information in Ontario	963,083	963,083	18
Basic Stand Alone Inpatient Claims (BSA)	Claim-level information from 2008 Medicare inpatient claims	588,415	588,415	6
California Hospital Discharge (California)	Hospital discharge information from the HCUP state inpatient database for 2007	4,016,573	4,016,573	16
Canadian Community Health Survey (CCHS)	A pooled version of survey data across multiple years that gathers health information for the Canadian population	904,813	752,472	8
Canadian COVID-19 (COVID-19)	COVID-19 health records of Canadians collected by Esri Canada	1,384,881	745,623	7
FDA Adverse Event Reporting System (FAERS)	Adverse event and medication error reports submitted to FDA	881,204	251,409	7
Florida Hospital Discharge (Florida)	Hospital discharge information from the HCUP state inpatient database for 2007	2,327,563	2,327,563	12
Medical Information Mart for Intensive Care III (MIMIC-III)	Health-related information for patients who stayed in critical care units of the Beth Israel Deaconess Medical Center between 2001 and 2012	540,482	540,482	13
New York Hospital Discharge (New York)	Hospital discharge information from the HCUP state inpatient database for 2007	2,666,541	2,666,541	14
Nexoid COVID-19 Survival Calculator (Nexoid)	A web-based survey dataset on COVID-19 survival prediction collected by the Nexoid company in London, UK	968,408	968,394	19
Texas Inpatient Data (Texas)	Discharges from Texas hospitals	745,999	745,997	11
Washington State Hospital Discharge 2007 (Washington)	Hospital discharge information from the HCUP state inpatient database for 2007	644,902	644,901	8
Washington State Hospital Discharge 2008 (Washington2008)	Hospital discharge information from the HCUP state inpatient database for 2008	652,340	652,340	18

^*^After data transformation/removing observations with missing values on the outcome variable

For part 2 of the study, we show the seven smaller datasets that we used for our application case studies and comparisons in Table 2.

**Table 2:** A description of the seven datasets used in the case studies and evaluations in the second part of the study

Dataset	Description	No. observations (original)	No. observations^*^	No. Variables used in the analysis
Breast Cancer	Health information related to breast cancer recurrence in Yugoslavia	277	277	11
Breast Cancer Coimbra	Registry of women with breast cancer in Portugal between 2009 and 2013	116	116	10
Colposcopy/Schiller	One of three modality dataset related to subjective quality assessment of digital colposcopies	92	92	63
Danish Colorectal Cancer Group (DCCG)	Registry of all patients with colorectal cancer in Denmark since 2001	12,855	7,948 (700**)	11
Diabetic Retinopathy	Messidor image information related to signs of diabetic retinopathy	1151	600	20
Hot Flashes	A survey contains health information related to vasomotor symptoms for early breast cancer patients between 2020 and 2021	373	360	18
Thoracic Surgery	Post-operative life expectancy of patients who went through surgery for lung cancer between 2007 and 2011	470	470	17

^*^After data transformation/removing observations with missing values on the outcome variable

^**^The sample drawn for the evaluation which is different from the full clean dataset

### Augmentation scheme

Given a population dataset, the first step is to split it into training and test datasets, where the training dataset is used for subsequent sampling, augmentation and ML modeling, while the test data is retained for performance evaluation. In our augmentation scheme, outcome stratified random sampling was applied to draw 40 samples (base datasets) of sizes n_0_ without replacement, from the training data, where n_0_ $\in$ {20, 30, 40, 50, 60, 70, 80, 90, 100, 150, 200, 250, 300, 350, 400, 450, 500, 550, 600, 650, 700, 750, 800, 850, 900, 950, 1000, 2000, 3000, 4000, 5000, 6000, 7000, 8000, 9000, 10,000, 20,000, 30,000, 40,000, 50,000}. Then, each of the 40 base datasets was used to train a specific generative model. Subsequently, the synthetic records were simulated from that generative model with sizes according to the following geometric series. Let b ~ N(µ = 1.5, σ = 0.005) be a random variable that follows a normal distribution with a mean of 1.5 and a standard deviation of 0.005. The geometric series has more samples at low values and less at higher values as we expect there will be more variability at the lower end of the range.

A series contains 30 elements, and each element represents a size of synthetic dataset to be generated, denoted as n’ = [b^i+4^] (i = 1, … , 30), where [x] denotes rounding to the closest integer to x. Following this procedure, a total of 10 geometric series were created. The augmented dataset has a size of *n* = n_0_ + n’, which means that for each of the 40 values of n_0_, a total of 300 augmented datasets was generated of different sizes (i.e., different degrees of augmentation). Each of the augmented datasets was used to train an ML model. To ensure comparability of the results, the same testing dataset was used for all the augmented datasets for evaluation. In total 12,000 augmented datasets were therefore generated and evaluated for each of the 13 datasets.

### Machine learning analytic workload

In this study, the chosen workload ML model is a light gradient boosting machine (LGBM) [65]. Tree-based models are the most common type of ML prognostic methods used in clinical research [1]. They perform better than linear models, such as logistic regression [66–70], and have also been found to perform better than deep learning models on tabular datasets [71, 72].

Model tuning used 5-fold cross-validation and Bayesian optimization [73]. The range for the tuning parameters was previously suggested [74–77], and these are summarized in Appendix. High cardinality variables were converted to embeddings [78] using a scheme similar to target encoding. As noted earlier predictive model performance was evaluated using the ROC-AUC.

### Evaluating LGBM model performance

Model performance was evaluated using the ROC-AUC discrimination metric, which is one of the few recommended metrics for evaluating clinical prediction model performance [79]. Other metrics that are sometimes used are PR-AUC and the F1 score. These are deemed unsuitable in our context of comparing different clinically relevant datasets.

The PR-AUC is based on the precision-recall curve [80–89]. PR-AUC does not directly consider true negatives, in contrast to ROC-AUC. True negatives are important in clinical settings [79]. Furthermore, unlike ROC-AUC which is robust under class imbalance [90, 91], PR-AUC is not a universally superior metric in the presence of class imbalance [92]. Furthermore, the PR-AUC calculation is often approximated using the average precision [86, 93].

The F1 score is sensitive to class imbalance [94–98], making it unsuitable for studies involving multiple datasets like ours. F1 also ignores true negatives [79]. Another challenge with the F1 score is that it requires the definition of a threshold to dichotomize the predicted probability. In the context of a methodological research study, it is difficult to choose a non-arbitrary threshold because the prevalence varies with dataset, and the choice of threshold would depend on the clinical decision.

### Synthetic data generation methods

We used four commonly applied generative modeling methods to generate new observations for structured tabular data, namely, sequential decision trees [56, 99–101], Bayesian networks [102–105], conditional tabular generative adversarial network [106] and tabular variational autoencoders [106]. These four synthesis models are some of the most common ones used in the literature to synthesize data. An examination of the most commonly used modeling approaches shows that these four model types we considered represent a large proportion of methods used in practice [107].

The first method was implemented using Aetion® Generate, a commercial product from Aetion^1^, and the last three methods were implemented using an open-sourced Python package, Synthcity [108]. Our adaptation of Synthcity, which is publicly available, provides further pre-processing and post-processing on top of Synthcity. In the experiments, the variables to be synthesized in each dataset are only those that were used in the analysis (those in the last column in Tables 1 and 2).

#### Sequential decision trees

Similar to using a chaining method for multi-label classification problems, sequential decision trees (SEQ) generate synthetic data using conditional trees in a sequential fashion [56, 109, 110]. It has been commonly employed in the healthcare and social science domains for data synthesis [59, 99, 100, 111–116]. Sequential decision trees can accommodate continuous and categorical variables in the modeling process. The details of the implementation procedures can be referred to [56].

#### Bayesian networks

Bayesian Networks (BN) are models based on Directed Acyclic Graphs that consist of nodes representing the random variables and arcs representing the dependencies among these variables. To construct the BN model, the first step is to find the optimal network topology, and then to estimate the optimal parameters [102]. Starting with a random initial network structure, the Hill Climb heuristic search is used to find the optimal structure. Then, the conditional probability distributions are estimated using the maximum a posteriori estimator [117]. Once the network structure and the parameters are estimated, we can initialize the nodes with no incoming arcs by sampling from their marginal distributions and predict the rest of the connected variables using the estimated parameters.

#### Conditional tabular generative adversarial network

A basic generative adversarial network (GAN) consists of two artificial neural networks (ANNs), a generator and a discriminator [57]. The generator and the discriminator play a min-max game. The input to the generator is noise, while its output is synthetic data. The discriminator has two inputs: the real training data and the synthetic data generated by the generator. The output of the discriminator indicates whether its input is real or synthetic. The generator is trained to ‘trick’ the discriminator by generating samples that look real. On the other hand, the discriminator is trained to maximize its discriminatory capability.

Among all the variations of GAN architectures, the conditional tabular GAN (CTGAN) is often used in tabular data synthesis [118]. CTGAN builds on the traditional GANs by addressing the non-Gaussian and multimodal distributions of continuous variables and the highly imbalanced categorical variables [106]. CTGAN solves the first problem by proposing a per-mode normalization technique. For the second problem, each category of a categorical variable serves as the condition passed to the GAN.

#### Tabular variational autoencoder

Variational autoencoders (VAE) use ANNs and involve two steps (encoding and decoding) to generate new samples [58]. First, an encoder is generated to compress input data into a lower-dimensional latent space, in which the data points are represented by distributions. The second step is a decoding process, in which new data samples are reconstructed as output from the latent space. The neural network is optimized by minimizing the reconstruction loss between the output and the input. VAEs are known to generate complex data of various types due to its ability to learn more complex distributions [119]. Many variants have been proposed as an extension of VAE, such as triplet-based VAE [120], conditional VAE [121], and Gaussian VAE [122]. In particular, the tabular VAE (TVAE) was proposed as an adaption of the standard VAE to model and generate mixed-type tabular data with a modified loss function [106].

### Generalized linear mixed effect model for assessing augmentation

Based on the characteristics of the input base datasets, we fit a generalized linear mixed effect model to determine the characteristics that significantly influence the benefit of augmentation. The outcome for this model was determined by examining all of the simulation results for each n_0_ value for every dataset and every generative model, and a binary value was selected to indicate that for this {n_0_, dataset, generative model} combination augmentation improved ROC-AUC over the baseline (a one outcome) or not (a zero outcome). This resulted in 520 observations for every generative model.

Whether a dataset will benefit from a certain amount of augmentation depends on its complexity. For example, a simple dataset, which conceptually can mean a small dataset with few low cardinality categorical variables, is unlikely to have a marked increase in diversity after augmentation. This is because the space of possible values on the categorical variables is small. Whereas a more complex dataset with many high cardinality variables is likely to experience much more increases in diversity with augmentation and hence would be expected to perform better on unseen data.

Previous work on data complexity metrics [123, 124] and methods for sample size calculation that take data complexity into account [125, 126] have defined a set of metrics that we considered for our augmentation decision model. We propose that dataset complexity can be characterized by the following variables: the base dataset size n_0_, the number of predictor parameters, outcome distribution, standardized entropy, mutual information, separability measure and the ROC-AUC of the base dataset. These additional variables are defined as:

- **Base dataset size n**_**0**_. The number of records in the original dataset.
- **Degrees of freedom.** This is given a value of 1 for a numeric variable, and a categorical variable with k levels gives k − 1.
- **Imbalance factor.** The outcome distribution is represented by the imbalance. It describes the imbalance between the positive and negative classes in the binary outcome and is quantified as the maximum of prevalence/(1 – prevalence) and (1 – prevalence)/prevalence, where prevalence is the proportion of individuals who have a positive outcome. A lower imbalance factor implies a more balanced distribution of outcome classes in the dataset.
- **Standardized entropy.** This is calculated as the information for each predictor and the whole dataset. We take the mean of the standardized entropy across all predictors to reflect the average amount of information produced by the variables.
- **Mutual information.** The coefficient of variation across the mutual information calculated from all predictor pairs.
- **The separability measure.** This is defined as the ratio of the distance of intraclass nearest neighbors to the distance of interclass nearest neighbors to reflect the magnitude of distinguishability between two samples from different classes. To accommodate various types of variables for the intraclass and interclass distances, we further modify this measure by replacing the Euclidean distance with the Gower distance.

The simulated data are clustered, with the dataset constituting the clustering factor. A mixed effect model is suitable for clustered data and consists of both random and fixed effects, in which random effects capture the variation across the hierarchical or clustering groups in the dataset, while the fixed effects estimate the impact of a variable within a specific group [127]. In this study, augmentation patterns are highly likely to vary across different datasets. Therefore, to account for this clustering structure of the augmentation performance, the dataset was modeled as a random component. The characteristics of primary interest, including n_0_, imbalance factor, degrees of freedom, baseline ROC-AUC, number of predictors, entropy, mutual information and separability measure, were included as fixed effects to assess the statistical significance. The R package *lme4* was used to build the generalized linear mixed effect model with a logit link.

### Evaluation of augmentation

We illustrate the proposed augmentation method by applying it to real datasets and assessing whether this results in an improvement in the performance of the prediction model.

Seven real datasets were used: the Hot Flashes dataset, Danish Colorectal Cancer Group dataset, Breast Cancer Coimbra dataset, Breast Cancer dataset, Colposcopy/Schiller dataset, Diabetic Retinopathy dataset and Thoracic Surgery dataset. These datasets vary across dimensions and complexity. The detailed descriptions of the datasets are summarized in Appendix.

**Fig. 3:**
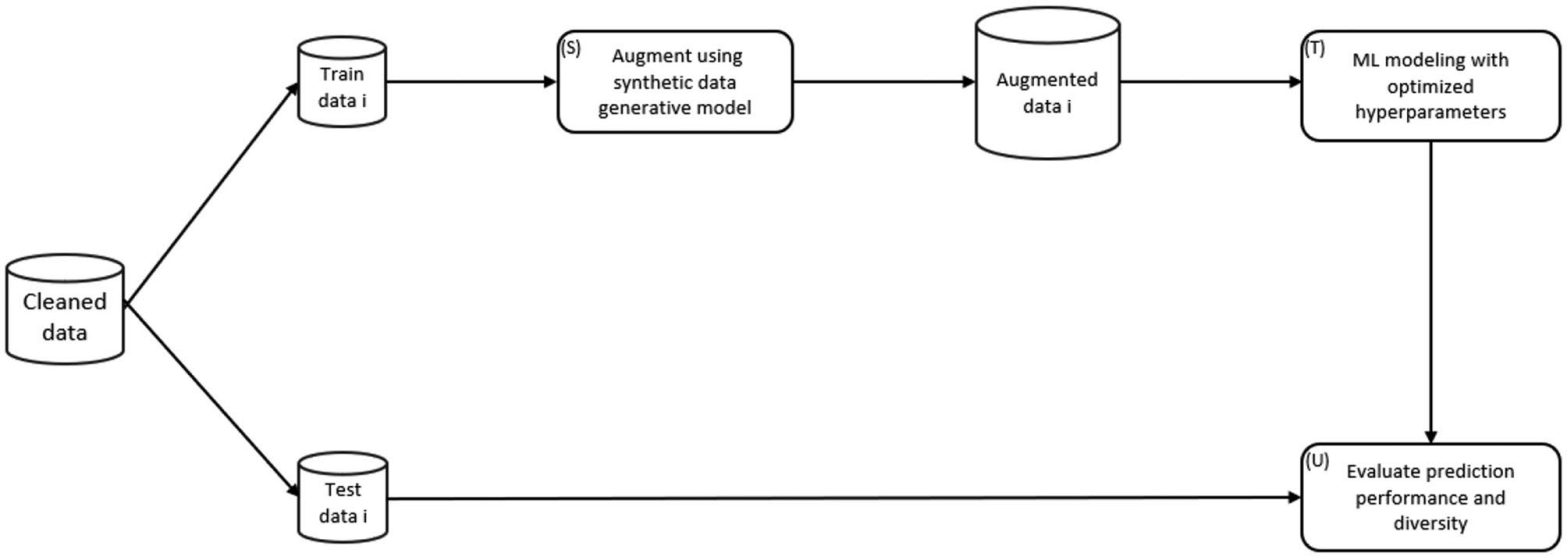
5-fold cross-validation procedure for case studies at iteration *i* (*i* = 1, … , 5). This is the process for the outer loop of nested cross-validation

A nested 5-fold cross-validation (CV) approach was applied for model training and prediction, which has been shown to yield almost unbiased estimates of model performance [128–130]. The whole process is summarized in the diagram in Fig. 3.

The original dataset was first preprocessed and then split into 5 pairs of training and testing data. For each pairs set *i* (*i* = 1, 2, … , 5), the eight characteristics of the analysis dataset were measured. The baseline ROC-AUC was determined as the average value of ROC-AUC obtained from the 5-fold training sets. Then, for each training data *i*, synthetic data were generated out of the four generative models and concatenated with the corresponding training data as the augmented data for model training (step S). The LGBM model was trained to examine the association between the outcome of interest and the data complexity measures (step T). The hyperparameters of the LGBM models were tuned and optimized using Bayesian optimization [73]. The range for the tuning parameters, specific to each model, was previously suggested [74–77]. It should be noted that augmentation was performed separately for each training partition in the outer loop to avoid data leakage that would result in optimistic model performance. A range of values for *n’* from 7 to 1 million was evaluated and remained the same for each iteration. The final ROC-AUC result was the averaged value of ROC-AUC across five iterations of the outer loop, and the *n’* value that provided the maximum ROC-AUC was deemed optimal (step U). To assess the improvements in ROC-AUC from the augmented datasets relative to the original datasets, we performed an exact permutation one-tailed test for the mean paired difference at an alpha level of 0.05.

### Evaluation of diversity

The objective of this analysis was to determine if improvements in the ROC-AUC of augmented data were due to the larger sample size or due to the generative models increasing the diversity of the datasets (which is the mechanism described in the literature).

#### Measuring diversity

Diversity is an important evaluation metric to assess the quality of generated synthetic data and is sometimes defined as the proportion of real data covered by the synthetic data [131, 132]. However, in our study, we are more interested in identifying synthetic data records that are significantly different from the original samples. In other words, a new data record is defined to be diverse if it is different (i.e., the extent to which it is an outlier or an anomaly) from the original sample. It is necessary to find an effective approach to detect the anomaly records in one dataset with reference to another one.

Since diversity is measured at the dataset level rather than an individual record level, one way to conceptualize diversity is to compare the multivariate variation in the original data and the augmented data. If augmentation results in greater variation, then that would be an indicator of greater data diversity. Several versions of multivariate coefficients of variation were introduced to measure the variability of populations using the characteristics of the numeric variables [133–136]. Another study proposed a method to determine the variability specifically for categorical data [137]. However, these methods are restricted to one type of variable, and our datasets have both categorical and numeric variables. An alternative approach is to examine methods for assessing data shift. Kamulete developed a data-driven approach, called D-SOS, to detect non-negligible adverse shifts in a sample using outlier scores [138]. In contrast to other statistical tests, D-SOS focuses on identifying distributions that are not benign but significantly shifting from the reference sample by placing more weights on instances in the outlying regions of the sample data. However, the contamination rate that aims to detect non-negligible adverse shifts is distribution-based and therefore, unsuitable for our context, which is to capture the amount of new and diverse observations.

Inspired by this idea, we designed a new metric to measure the diversity using outlier data records in the augmented dataset compared to the base dataset. A record in the augmented dataset is deemed to be an outlier using a score obtained from an extended isolation forest model trained on the base dataset. The extended isolation forest, an extension of the isolation forest, addresses the bias problem during the tree branching that arises in the standard isolation forest and therefore, is more robust in detecting anomalies [139, 140]. Then, the trained isolation forest model was applied to both the base and augmented datasets to predict the outlier score for each observation, where a larger predicted score indicates a higher possibility of an outlier record. An incremental sequence of thresholds τ_j_ was created from 0.01 to 1 with a step size of 0.01. Then, we calculated a threshold-dependent contamination rate quantified as the proportion of outliers in the data, which are the records with outlier scores equal to or exceeding τ_j_ at step j. For a given threshold, a higher contamination rate implies a greater percentage of outlier records, and consequently, the data are more diverse. The difference between the two contamination curves of the augmented and base datasets is the additional amount of diverse data records contributed to the original data by augmentation. We are only interested in the positive difference, as the negative difference means the contamination rate of the augmented data does not provide any meaningful increment in the diversity. Thus, the diversity metric is defined as follows:

1 $$diversity = {{\mathop \sum \nolimits_{j = 1}^{100} \{ 1\left( {{x_j} \ge 0} \right) \cdot \left( {{x_j}\left( {2 - {x_j}} \right)} \right) + 1({x_j} < 0) \cdot 0\} } \over {100}} , $$

where 1(·) is the indicator function, and ${{\rm{x}}_{\rm{j}}}$ represents the difference between the contamination rates of the augmented and base datasets at the threshold τ_j_. Thus, if the difference in the contamination rates is zero or positive, we calculate the diversity using a weighted contamination rate difference, which is always non-negative.

The precise steps of the calculation are included in Appendix.

#### Evaluating the impact of diversity

In addition to the four generative models, we included the bootstrap method as another approach to augment the base dataset by resampling the original records with replacement. The purpose of including the bootstrap method is to rule out the influence of increasing data size. Sampling with replacement as an augmentation method is expected to have a minimal impact on diversity. The size of the additional data that were sampled with replacement was the same as the amount of synthetic data generated from the generative model that led to the optimal performance.

Two comparisons are relevant here: (a) comparing the diversity between bootstrapped data and model-augmented data, where we expect that the latter would have a higher diversity, and (b) comparing the predictive model performance between bootstrapped data and model-augmented data, where we expect that the latter would have a higher predictive performance. While this does not demonstrate causality, if supported, it would provide evidence that higher diversity is associated with higher predictive model performance, and that increasing sample size alone does not explain predictive model performance improvement.

For each dataset, the diversity was averaged across the five iterations as the final diversity values for both the best generative model and bootstrap (step U). One-tailed exact permutation tests of the mean paired difference were performed to compare the diversity of the datasets and of the ROC-AUC results with resampling and generation. An alpha level of 0.05 was used.

## Results

### Overall augmentation performance

In this section, the performance of data augmentation against the size of synthetic data *n’* in 40 different *n*_*0*_ scenarios is summarized. To make the trends more interpretable and visible, the scales for the y-axis are varied, and the logarithm is taken for n’. Loess regression was used to fit a smooth curve for each generative model.

In the main body of the paper, we present results for the BSA and FAERS datasets. The results for the remaining datasets are included in Appendix. These two datasets were selected for inclusion in the main body since the former is a simple dataset and the latter is quite a complex dataset (with multiple variables with high cardinality). They illustrate the findings across the range of data complexity. The conclusions drawn from these two datasets are consistent with those from the other datasets.

**Fig. 4:**
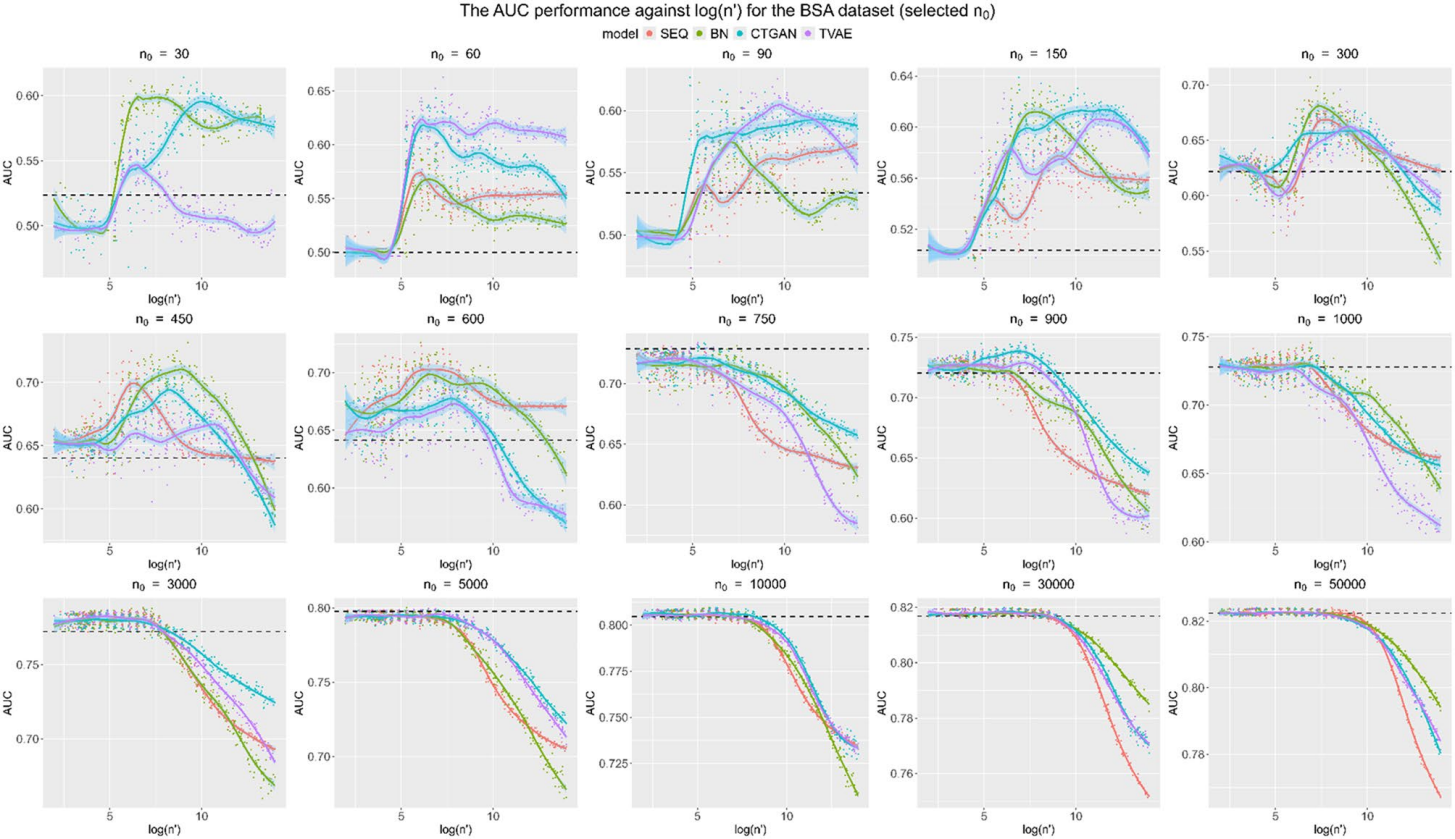
Augmentation performance of ROC-AUC against log (*n’*) for the BSA dataset for a subset of the baseline data sizes. The black dotted line is the baseline ROC-AUC for the base dataset of size *n*_*0*_

**Fig. 5:**
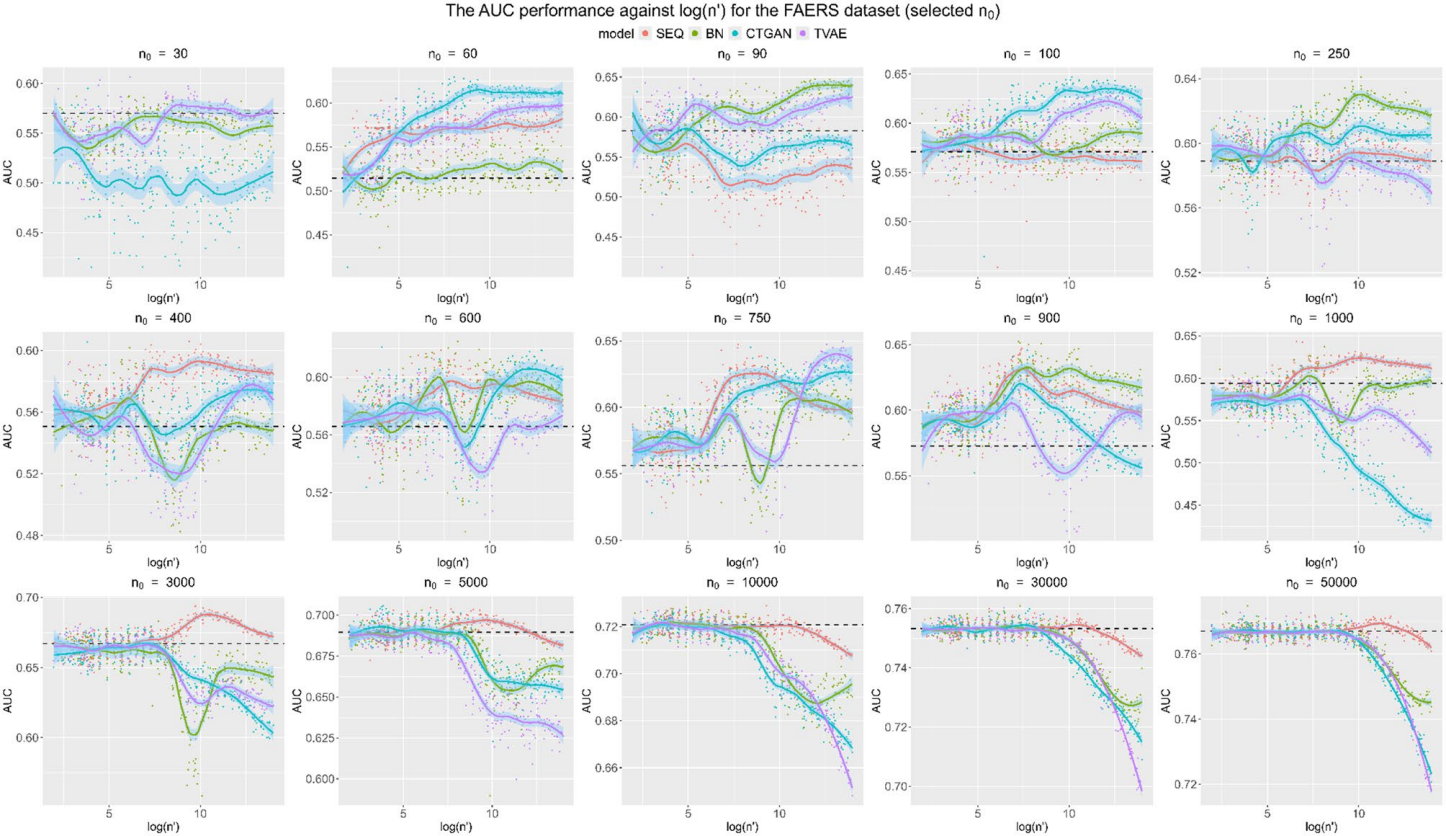
Augmentation performance of ROC-AUC against log(*n’*) for the FAERS dataset for a subset of the baseline data sizes. The black dotted line is the baseline ROC-AUC for the base dataset of size *n*_*0*_

In Figs. 4 and 5, it can be clearly seen that the augmentation can improve the performance measured by ROC-AUC, as more synthetic data are incorporated, especially for small and medium *n*_*0*_. In fact, the improvements in model performance as measured by the ROC-AUC can be nontrivial, in some cases exceeding absolute increases of 0.1. For the large *n*_*0*_, the improvement from augmentation is less or there is even deterioration. In addition, the performance of SDG models varies significantly across different *n*_*0*_ and base datasets, demonstrating the importance of identifying the most appropriate model in a specific situation. Moreover, compared to the BSA dataset, the FAERS dataset benefits more from data augmentation, as the highest *n*_*0*_ with noticeable improvement is relatively larger, around *n*_*0*_ = 3,000, whereas the highest *n*_*0*_ with noticeable improvement for the BSA dataset is approximately 650. Since the FAERS dataset is more complex with higher cardinality variables, further augmentation may generate more plausible values from the population, which leads to a more diverse augmented dataset compared to the BSA dataset.

### Generalized linear mixed effect model

The fixed effect estimates and associated p-values were summarized in Table 3 for the “augmentation benefit” model. The corresponding odds ratio estimates and 95% confidence intervals (CI) were presented in Table 4. To make the estimates comparable, a standardized version was provided for each type of estimate by standardizing the relevant characteristics.

**Table 3:** Fixed effect estimates and associated p-values for the generalized linear mixed effect model

	Unstandardized		Standardized	
Variable	Fixed effect estimate	*P*-value	Fixed effect estimate	*P*-value
**intercept**	**9.86**	**0.0393**	**1.17**	** < 0.0001**
n_0_	−2.38x10^−5^	0.0789	−0.26	0.1195
imbalance factor	−7.50x10^−3^	0.9411	−0.03	0.9391
**degrees of freedom**	**3.89x10**^**−4**^	**0.0054**	**0.47**	**0.0139**
**baseline ROC-AUC**	**−12.65**	**0.0009**	**−1.62**	**0.0010**
number of predictors	−9.91x10^−3^	0.9249	−0.05	0.9164
entropy	−2.26x10^−2^	0.9939	−0.01	0.9833
mutual information	0.10	0.8630	0.06	0.8506
separability measure	0.83	0.5044	0.19	0.5258

**Table 4:** The unstandardized and standardized odds ratio estimates and 95% confidence intervals for the generalized linear mixed effect model

	Unstandardized			Standardized		
Variable	Odds ratio estimate	95% CI (lower)	95% CI (upper)	Odds ratio estimate	95% CI (lower)	95% CI (upper)
**intercept**	**1.91x10**^**4**^	**1.62**	**2.25x10**^**8**^	**3.24**	**1.87**	**5.58**
n_0_	1.00	9.99x10^−1^	1.00	0.77	0.56	1.07
imbalance factor	0.99	0.81	1.21	0.97	0.47	2.02
**degrees of freedom**	**3.89x10**^**−4**^	**1.15x10**^**−4**^	**6.62x10**^**−4**^	**1.61**	**1.10**	**2.34**
**baseline ROC-AUC**	**3.22x10**^**−6**^	**1.89x10**^**−9**^	**5.47x10**^**−3**^	**0.20**	**0.08**	**0.52**
number of predictors	0.99	0.81	1.22	0.95	0.37	2.43
entropy	0.98	2.93x10^−3^	325.70	0.99	0.36	2.70
mutual information	1.11	0.35	3.45	1.06	0.59	1.90
separability measure	2.30	0.20	26.69	1.21	0.67	2.19

Both tables show that the unstandardized and standardized results are consistent in terms of the variable importance. The baseline ROC-AUC has the biggest impact on augmentation benefit, followed by the degrees of freedom as the second influential factor (highlighted in bold). The estimation results indicate that the datasets with lower baseline ROC-AUC and higher cardinality are more likely to benefit from augmentation. In addition, augmentation is also advantageous for smaller, more balanced data, and those with lower dimensions, more predictable variables, higher variability in variable dependency and lower outcome class separability, although these factors were not statistically significant.

The base data size, n_0_, was not found to be significant in these models. This may be due to that relationship being non-linear as observed in the previous plots, and these models were fitting a linear relationship.

### Evaluation of augmentation and diversity

The four generative models were employed to simulate additional datasets. Table 5 presents the augmentation results for each dataset, the generative model that leads to the optimal performance, the amount of synthetic data records needing to be generated to achieve the optimal performance and the performance using the bootstrap method.

**Table 5:** Analysis results of augmentation performance for the seven datasets

			ROC-AUC Results				Diversity Results	
Dataset	Model	**n’**_**max**_	Baseline ROC-AUC	Augmented ROC-AUC	Relative ROC-AUC (%)	Resampled ROC-AUC	Diversity generative	Diversity resample
Breast Cancer	CTGAN	25	0.7143	0.7451	4.31	0.6729	0.0017	0.0008
Breast Cancer Coimbra	BN	53	0.7392	0.8722	18.00	0.8291	0.0061	0.0019
Colposcopy/Schiller	CTGAN	2,205	0.5125	0.7341	43.23	0.6116	0.0883	0.0004
Danish Colorectal Cancer Group	TVAE	720	0.7171	0.7780	8.50	0.7077	0.0000	0.0008
Diabetic Retinopathy	BN	11,534	0.7400	0.7974	7.75	0.7299	0.1177	0.0002
Hot Flashes	CTGAN	720	0.7161	0.7668	7.08	0.6477	0.0023	0.0013
Thoracic Surgery	TVAE	6,602	0.5584	0.6700	19.98	0.6914	0.0000	0.0003

n’_max_: n’ that leads to maximum ROC-AUC. Baseline ROC-AUC: baseline ROC-AUC from the base data. Augmented ROC-AUC: maximum ROC-AUC from the augmented data. Resampled ROC-AUC: ROC-AUC from the augmented data with a size of n’_max_ using resampling with replacement method. Diversity generative: diversity of data augmented using a generative model. Diversity resample: diversity of data augmented using the bootstrap method

The baseline ROC-AUC values are within the range from poor to good [141]. The additional synthetic data sizes vary depending on both the generative model that was used and the dataset. As expected, the best generative model is not uniform.

The relative improvement in ROC-AUC due to generative model augmentation is remarkably high, ranging from 4.3% to 43.23% (average 15.55%), indicating a substantial gain in model performance after augmentation (baseline ROC-AUC vs augmented ROC-AUC: *p* = 0.0078). The resampling augmentation generally yields a much lower ROC-AUC, compared to the synthetic data generative models and on some occasions is even worse than the baseline scenario without augmentation (augmented ROC-AUC vs resampled ROC-AUC: *p* = 0.016). Increasing the sample size by resampling the original data often does not contribute to the improvement of model performance as much as the other synthetic data generative models.

The diversity results for the resampled data are generally lower than those for the data augmented using the generative models (generative diversity vs. resampled diversity: *p* = 0.046). Therefore, augmentation using the generative models does increase the diversity of the datasets beyond just a simple increase in the sample size from the original data distribution.

## Discussion

### Summary

The availability of health data for research purposes is limited, and these datasets are often small. However, training of ML models requires large amounts of data to obtain optimal performance on unseen data, and training on datasets that are too small can lead to model instability [142], and to overfitting and an inability to generalize predictions to unseen data [3, 143], even under ideal conditions (e.g., no data shift or drift). Consequently, the conclusions drawn from such models may be unstable and inaccurate. In such cases, data augmentation can be beneficial by simulating more, and more diverse, data based on the existing data.

Although it has been receiving increasing attention in recent years, especially in imaging data, time series data, text, and gene expression data applications, tabular clinical data augmentation has not been extensively evaluated, despite data augmentation being one of the primary use cases for synthetic data generation methods [144]. In this study, we fill this gap by evaluating the benefits of data augmentation for tabular health data.

The descriptive results from our simulations show that augmentation for small datasets can be beneficial in terms of ROC-AUC, and that excessive augmentation can reduce predictive model performance. The appropriate level of augmentation that maximizes performance differs for each dataset. However, the benefits of augmentation are less obvious or even detrimental for large datasets. Our generalized linear mixed effect model highlights that the improvements in predictive performance are most likely for more complex datasets or datasets with lower baseline ROC-AUC.

Our interpretation of this phenomenon is that with small or moderate data size to start, the simulated data positively contributes by increasing size and diversity, and thus, are more likely to add information that is similar to the unseen dataset. In contrast, for a large base dataset, the increase in size has less marginal predictive benefit, whereby the dataset may already contain sufficiently diverse information, and incorporating more simulated data is less likely to provide useful diversity. In fact, it may be increasing the unnecessary noise in the current dataset and hence weakening the relationships with the outcome. Moreover, the simpler datasets with fewer categorical variables and lower cardinality were found to benefit less from augmentation, and this is arguably because the space to increase diversity is limited (i.e., simulated records will look more like current records rather than be different). That lower baseline ROC-AUC benefits more from augmentation can be attributed to a ceiling effect, where higher ROC-AUC values will likely benefit less from augmentation.

Several studies reveal the significant enhancements in prediction performance from data augmentation on genomic data that are small but high-dimensional in nature [145–148], which may have thousands of categories (e.g. k-mer analysis) [149, 150]. Our results are not directly comparable as our analysis did not consider such high-dimensional datasets – data used for clinical prediction models tend to have lower dimensionality.

The typical ROC-AUC range in genomic disease prediction studies is generally between 0.55 and 0.8 for the most common complex diseases [151–153], and it is uncommon to have ROC-AUC exceed 0.8, except for rare diseases with high heritability [154]. At the low end of that range, our results suggest that improvements in predictive performance due to augmentation are to be expected.

Different generative models perform best depending on the dataset itself and its baseline size. Therefore, it is not possible to a priori say that a particular generative model is consistently superior for the augmentation task. Multiple SDG models need to be evaluated to find the best one to augment a particular dataset.

Our application of augmentation to seven small datasets further confirms the model performance improvement through augmenting the original dataset. These datasets resulted in model performance improvement ranging from 4.31% to 43.23% using the generative models (average 15.55%), whereas the datasets augmented with only resampling did not consistently perform better than that. We presented evidence showing that diversifying the existing data through synthetic data augmentation plays an important role in enhancing model performance, and therefore, increasing the sample size without making the data more diverse is not as beneficial.

A recent smaller-scale study of data augmentation on tabular data similarly did not find a predominant generative model [155], which is why our recommendation of evaluating multiple models on each dataset and selecting the best-performing one gives more reliable augmentation outcomes. Furthermore, the previous study did not examine the relationship between base sample size and degree of augmentation and did not consider data complexity and baseline model performance. Hence, the conclusions of that study were quite limited in this regard.

Previous work on the augmentation of longitudinal EHR data using generative models demonstrated improvements in prediction accuracy on a handful of datasets [156]. However, our results on tabular data show that augmentation depends on the data characteristics, the specific generative model used, and the degree of augmentation, and therefore will not always be beneficial. In addition, our findings suggest that dynamic selection among multiple generative models to identify the best one given the specific data parameters provides better results.

### Recommendations for practice and research

For datasets where the baseline ROC-AUC is high, augmentation may not provide a significant advantage. However, where the baseline ROC-AUC is medium or small, and where dataset sizes are in the 100 to 3,000 observations range, augmentation can potentially improve the performance of a model’s ROC-AUC, sometimes by a considerable amount. Datasets with high cardinality categorical variables can also benefit from augmentation. In contrast, augmentation will likely be less beneficial for large and simple datasets with strong relationships with the outcome (i.e., higher baseline ROC-AUC).

Analysts can try different degrees of augmentation using multiple generative models and evaluate them on holdout data to determine the amount of augmentation which can maximize the prognostic performance.

It should be noted that the training dataset for the generative models needs to be separated from the testing dataset. This is easier to do in a simple train/test split scenario. However, if augmentation is used in the context of, say, 5-fold cross-validation, then the generative models should be trained on the 4/5 training splits each time and evaluated on the remaining 1/5 split. This will ensure that there is no data leakage, which would result in optimistic results that would not carry over to unseen data in subsequent applications. For the final augmented dataset, the determined n’_max_ simulated records should be concatenated to the original dataset.

### Limitations and future work

Evaluating the performance of each dataset at different levels of augmentation can be computationally intensive. This means that the processing time to determine the best level of augmentation may not be small in practice.

Our analysis assumed that resampling with replacement was a good proxy for increasing the sample size without increasing diversity. The reasoning was that adding observations from the same distribution would have a minimal impact on diversity.

When datasets are small, some of the types of generative models that we used in our study have a higher risk of overfitting. However, the data dimensionality that was used has also tended to be low, which is a mitigating factor. And the default hyperparameters that were used for the generative models tended to train smaller models and hence reduced the opportunities for overfitting. Nevertheless, future work should examine generative models that are suited for small datasets, such as those based on pre-trained models.

Synthetic data generation has been shown to introduce bias in the generated data relative to the training data [157], and these biases are propagated across multiple generations of generative models (where the output of one is used as training for the next one) [158]. Our study did not examine the impact of augmentation on fairness. The impact of augmentation on fairness is an open question that should be the subject of further studies.

Data amplification, which is when more synthetic data is generated relative to the base dataset that was used by the generative model, has been shown not to improve the quality of population inferences nor the replicability of results for statistical models [159]. Amplification is different from augmentation in that amplified data does not include any of the original data within it. Our results did not consider population inferences or replicability. However, it would be informative for future work to examine whether augmentation gives different conclusions with respect to population inferences.

In addition to LGBM, we considered using other ensemble ML models, such as random forest, to examine the augmentation performance. However, several recent studies conclude that random forest models have less stable and generalizable performance due to overfitting on small samples [142, 160–162]. This is a worthwhile challenge that needs further investigation in future work. At present, we only present the LGBM results in this article.

Given our results showing augmented datasets with greater diversity have a higher improvement in predictive performance, further work can optimize generative models to specifically increase the diversity of the synthetic data to maximize the performance improvement for downstream ML workloads.

More recent predictive models, such as Tabular Prior-data Fitted Network (TabPFN) [163], were not considered in our analysis. As noted, tree-based machine learning models are the most commonly used ones in clinical predictive modeling work, and boosted trees have had consistently better performance than other approaches thus far, making them a suitable choice for this study. Future work should extend the current analysis to TabPFN to determine whether it can benefit from further augmentation.

## Electronic supplementary material

Below is the link to the electronic supplementary material.

Supplementary material 1

## Data Availability

The following provides information on the availability of each of the datasets used in this study: (1) Better Outcomes Registry & Network (BORN) | The BORN collects Ontario’s prescribed perinatal, newborn and child registry with the role of facilitating quality care for families across the province. It can be accessed through a data request at https://bornontario.ca/en/data/data.aspx. (2) Basic Stand Alone (BSA) The BSA inpatient claims dataset is about claim-level information that each record is an inpatient claim incurred by a 5% sample of Medicare beneficiaries. The dataset is publicly available at https://www.cms.gov/data-research/statistics-trends-and-reports/basic-stand-alone-medicare-claims-public-use-files/bsa-inpatient-claims-puf. (3) California State Hospital Discharge The California dataset contains the patient’s hospital 2008 discharge data from California, State Inpatient Databases (SID), Healthcare Cost and Utilization Project (HCUP), Agency for Healthcare Research and Quality [164], and is available for purchase at https://hcup-us.ahrq.gov/tech_assist/centdist.jsp. (4) Canadian Community Health Survey (CCHS) The CCHS data are Canadian population-level information concerning health status, health system utilization and health determinants collected by Statistics Canada through telephone survey. The availability of CCHS data is restricted and requires an access request at https://www150.statcan.gc.ca/n1/pub/82-620-m/2005001/4144189-eng.htm. (5) COVID-19 The COVID-19 dataset collects Canadian health records of COVID-19 gathered by the Public Health Agency of Canada and is available at Esri Canada (https://resources-covid19canada.hub.arcgis.com/). (6) FDA Adverse Event Reporting System (FAERS) The FAERS is a database comprising the information on adverse events and medication error reports submitted to FDA and can be downloaded at https://open.fda.gov/data/faers/. (7) Florida State Hospital Discharge The Florida dataset contains the patient’s hospital 2007 discharge data from Florida, State Inpatient Databases (SID), Healthcare Cost and Utilization Project (HCUP), Agency for Healthcare Research and Quality [164], and is available for purchase at https://hcup-us.ahrq.gov/tech_assist/centdist.jsp. (8) MIMIC-III MIMIC-III is a large database that contains deidentified health-related data associated with over forty thousand patients who stayed in critical care units of the Beth Israel Deaconess Medical Center between 2001 and 2012 [165, 166]. The access to the MIMIC database is upon signing a data use agreement with PhysioNet at https://physionet.org/content/mimiciii/1.4/[167]. (9) New York State Hospital DischargeThe New York dataset contains the patient’s hospital 2007 discharge data from New York, State Inpatient Databases (SID), Healthcare Cost and Utilization Project (HCUP), Agency for Healthcare Research and Quality [164], and is available for purchase at https://hcup-us.ahrq.gov/tech_assist/centdist.jsp. (10) COVID-19 Survival (Nexoid) The COVID-19 survival dataset is a web-based survey data collected by a company called Nexoid in United Kingdom. It is publicly available at https://www.covid19survivalcalculator.com/en/download. (11) Texas Hospital Discharge The Texas dataset contains the patient’s hospital discharge information for the first quarter of 2012 from Texas in the United States [168], and is publicly available at https://www.dshs.texas.gov/center-health-statistics/chs-data-sets-reports/texas-health-care-information-collection/health-data-researcher-information/texas-inpatient-public-use. (12) Washington State Hospital Discharge 2007 The Washington dataset contains the patient’s hospital 2007 discharge data from Washington, State Inpatient Databases (SID), Healthcare Cost and Utilization Project (HCUP), Agency for Healthcare Research and Quality [164], and is available for purchase at https://hcup-us.ahrq.gov/tech_assist/centdist.jsp. (13) Washington State Hospital Discharge 2008 The Washington2008 dataset contains the patient’s hospital 2008 discharge data from Washington, State Inpatient Databases (SID), Healthcare Cost and Utilization Project (HCUP), Agency for Healthcare Research and Quality [164], and is available for purchase at https://hcup-us.ahrq.gov/tech_assist/centdist.jsp. (14) Hot Flashes The Hot Flashes dataset stores the health information of patients with early breast cancer who experienced vasomotor symptoms, and the access request is available by contacting the senior authors of the original article. (15) Danish Colorectal Cancer Group. The Danish Colorectal Cancer Group (DCCG) dataset comprises all patients with colorectal cancer in Denmark between 2001 and 2018. The DCCG dataset can be requested from the Danish Colon Cancer registry. (16) Breast Cancer Coimbra. The Breast Cancer Coimbra dataset contains women with breast cancer recruited by the Gynaecology Department of the University Hospital Centre of Coimbra between 2009 and 2013 and is publicly available at https://archive.ics.uci.edu/dataset/451/breast+cancer+coimbra. (17) Breast Cancer. The Breast Cancer dataset collects information by the University Medical Centre, Institute of Oncology, Ljubljana, Yugoslavia, and is publicly available at https://archive.ics.uci.edu/dataset/14/breast+cancer. (18) Colposcopy/schiller. The Colposcopy/schiller dataset is one of three modality datasets that collects subjective quality assessment of digital colposcopies and is publicly available at https://archive.ics.uci.edu/dataset/384/quality+assessment+of+digital+colposcopies. (19) Diabetic Retinopathy. The Diabetic Retinopathy dataset extracts health information from the Messidor image set and is publicly available at https://archive.ics.uci.edu/dataset/329/diabetic+retinopathy+debrecen. (20) Thoracic Surgery. The Thoracic Surgery dataset describes the post-operative life expectancy of patients who underwent lung resections for primary lung cancer between 2007 and 2011 and is publicly available at https://archive.ics.uci.edu/dataset/277/thoracic+surgery+data.

## Footnotes

1 See <https://aetion.com/products/generate/>

## Code availability

The code used in this analysis can be accessed as follows: The synthetic data generation code is available in the pysdg package, available from: https://osf.io/xj9pr/. The machine learning modeling was performed using the R sdgm package available from: <  https://osf.io/DCJM6 > . The R code for applying the results on a new dataset is available from <  https://osf.io/4gu62/>.
