# Supplementary material for: Augmenting small tabular health data for training prognostic ensemble machine learning models using generative models
Source: BMC Med Inform Decis Mak. 2025 Nov 28;25:435. doi: 10.1186/s12911-025-03266-3 (PMC12661835; doi:10.1186/s12911-025-03266-3)
Supplement: Supplementary file 1 — Supplementary material 1 [file 12911_2025_3266_MOESM1_ESM.pdf]

Appendix

Augmenting small tabular health data for training prognostic ensemble machine learning models using generative models

Contents

Appendix A - Steps for Calculating Diversity ... 3
Appendix B - Results for augmentation performance (ROC-AUC) ... 4
B.1 BORN Dataset ... 5
B.2 BSA Dataset ... 7
B.3 California Dataset ... 9
B.4 CCHS Dataset ... 11
B.5 COVID-19 Dataset ... 13
B.6 FAERS Dataset ... 15
B.7 Florida Dataset ... 17
B.8 MIMIC-III Dataset ... 19
B.9 New York Dataset ... 21
B.10 Nexoid Dataset ... 23
B.11 Texas Dataset ... 25
B.12 Washington Dataset ... 27
B.13 Washington2008 Dataset ... 29
Appendix C - Results for case studies ... 31
C.1 Application to Breast Cancer Dataset ... 31
C.2 Application to Breast Cancer Coimbra Dataset ... 31
C.3 Application to Colposcopy/Schiller Dataset ... 32
C.4 Application to Danish Colorectal Cancer Group Dataset ... 33
C.5 Application to Diabetic Retinopathy Dataset ... 34
C.6 Application to Hot Flashes Dataset ... 35
C.7 Application to Thoracic Surgery Dataset ... 36
Appendix D - Hyperparameters ... 37
Appendix E – Details of the datasets ... 38
E.1 Better Outcomes Registry & Network ... 38

E.2 Basic Stand Alone Inpatient Claims 40
E.3 California Hospital Discharges 41
E.4 Canadian Community Health Survey 43
E.5 Canadian COVID-19 44
E.6 FDA Adverse Event Reporting System 45
E.7 Florida Hospital Discharges 46
E.8 Medical Information Mart for Intensive Care III 48
E.9 New York Hospital Discharges 49
E.10 Nexoid COVID-19 Survival Calculator 51
E.11 Texas Inpatients 53
E.12 Washington State Hospital Discharges 56
E.13 Washington State Hospital Discharges (2008) 57
E.14 Breast Cancer 59
E.15 Breast Cancer Coimbra 60
E.16 Colposcopy/Schiller 61
E.17 Danish Colorectal Cancer Group 65
E.18 Diabetic Retinopathy 67
E.19 Hot Flashes 69
E.20 Thoracic Surgery 71
F References 73

Appendix A - Steps for Calculating Diversity

The detailed implementation procedures of our proposed diversity metric are as follows.

1. Build an extended insolation forest model using the base dataset.
2. Apply the extended isolation forest model to the base dataset and calculate the outlier scores for the base dataset.
3. Apply the extended isolation forest model to the augmented dataset and calculate the outlier scores for the augmented dataset.
4. Generate a sequence of thresholds τj starting from 0.01 and increase to 1 by a step size of 0.01 such that τj ∈ {0.01, 0,02, …, 1}.
5. At each j, compute the contamination rate as the proportion of the outlier scores in the base dataset that is equal to or greater than τj.
6. At each j, compute the contamination rate as the proportion of the outlier scores in the augmented dataset that is equal to or greater than τj.
7. Find the difference between the two contamination rates.
8. Compute the diversity according to the equation in the body of the paper.

Our proposed metric enjoys several advantages. First, the metric is fairly straightforward to understand and implement. Secondly, it is data-driven and adaptive to any given dataset. Another strength is that it allows mixed data to be processed, offering flexibility to quantify the diversity between the two datasets.

Appendix B - Results for augmentation performance (ROC-AUC)

B.1 BORN Dataset

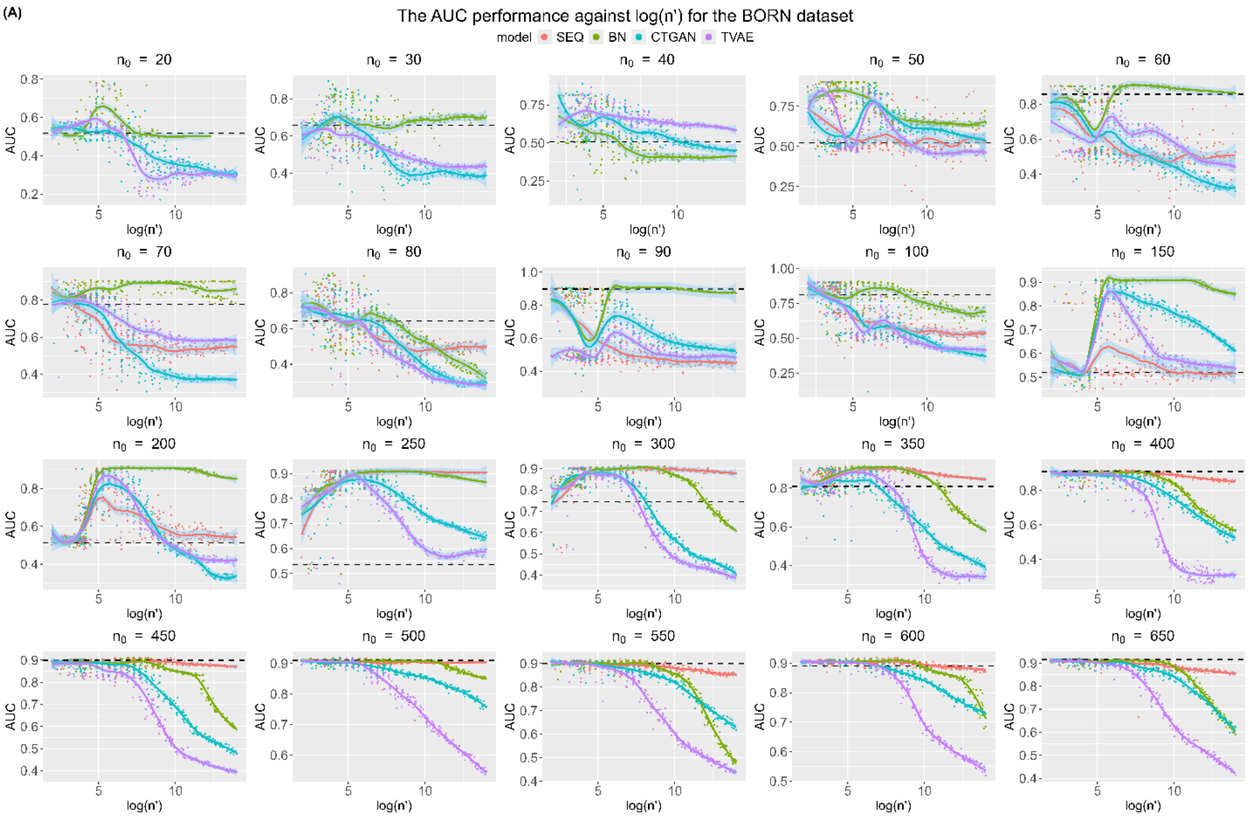

Figure B.1: Augmentation performance of ROC-AUC against log(n') for the BORN dataset (A).

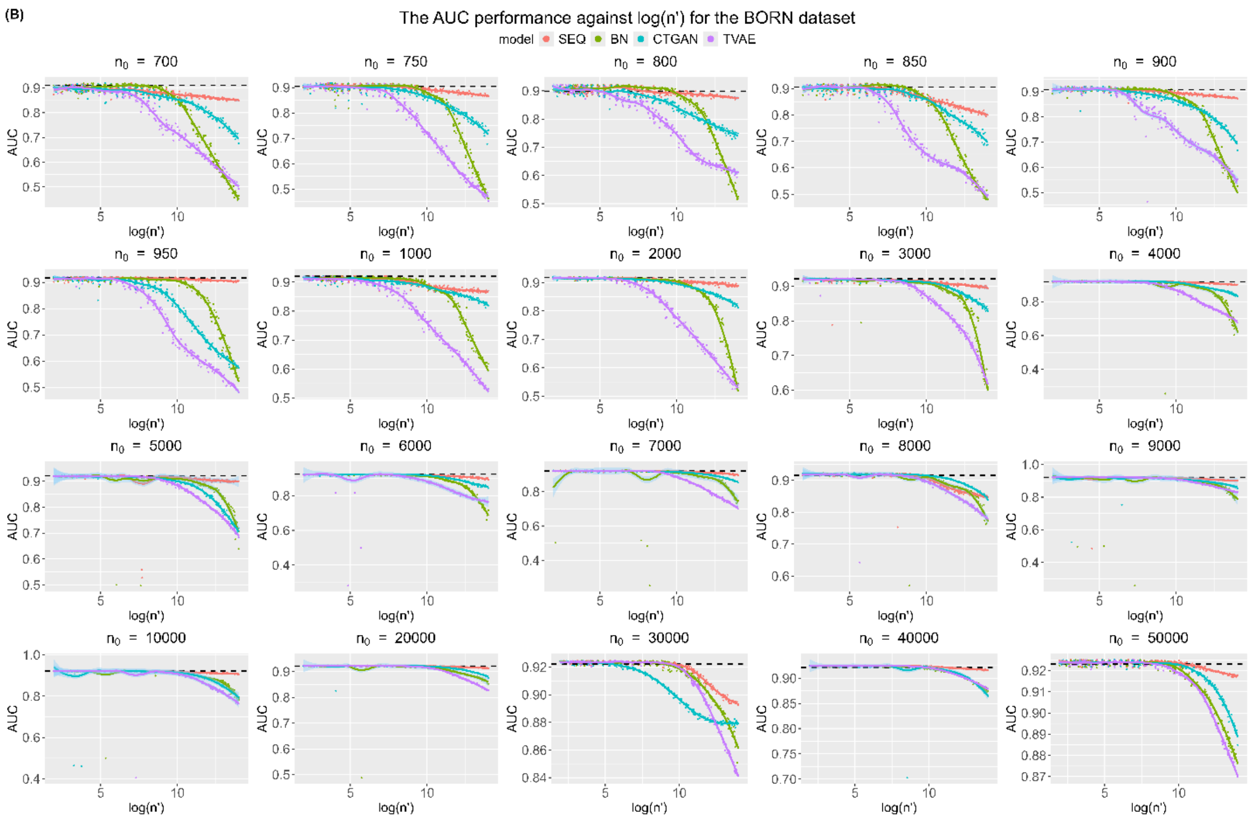

Figure B.2: Augmentation performance of ROC-AUC against log(n') for the BORN dataset (B).

B.2 BSA Dataset

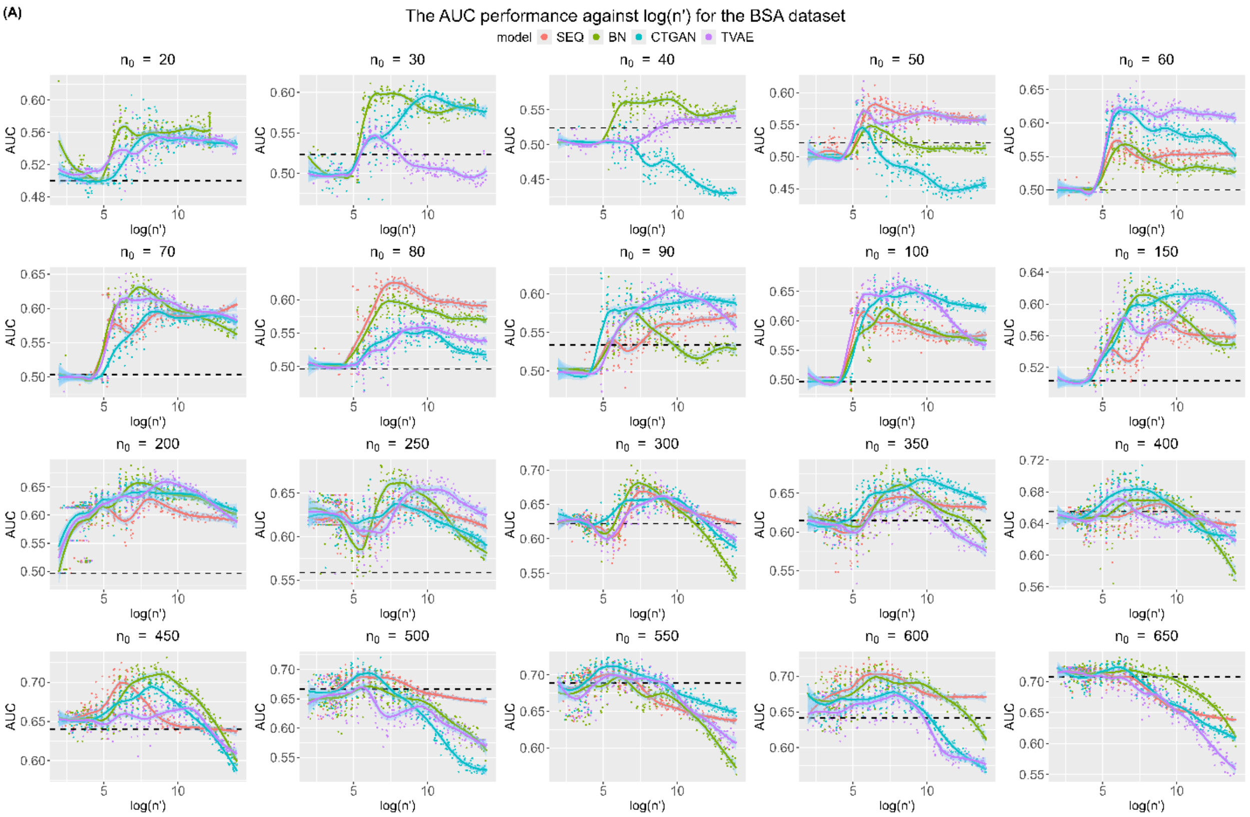

Figure B.3: Augmentation performance of ROC-AUC against log(*n′*) for the BSA dataset (A).

(B)

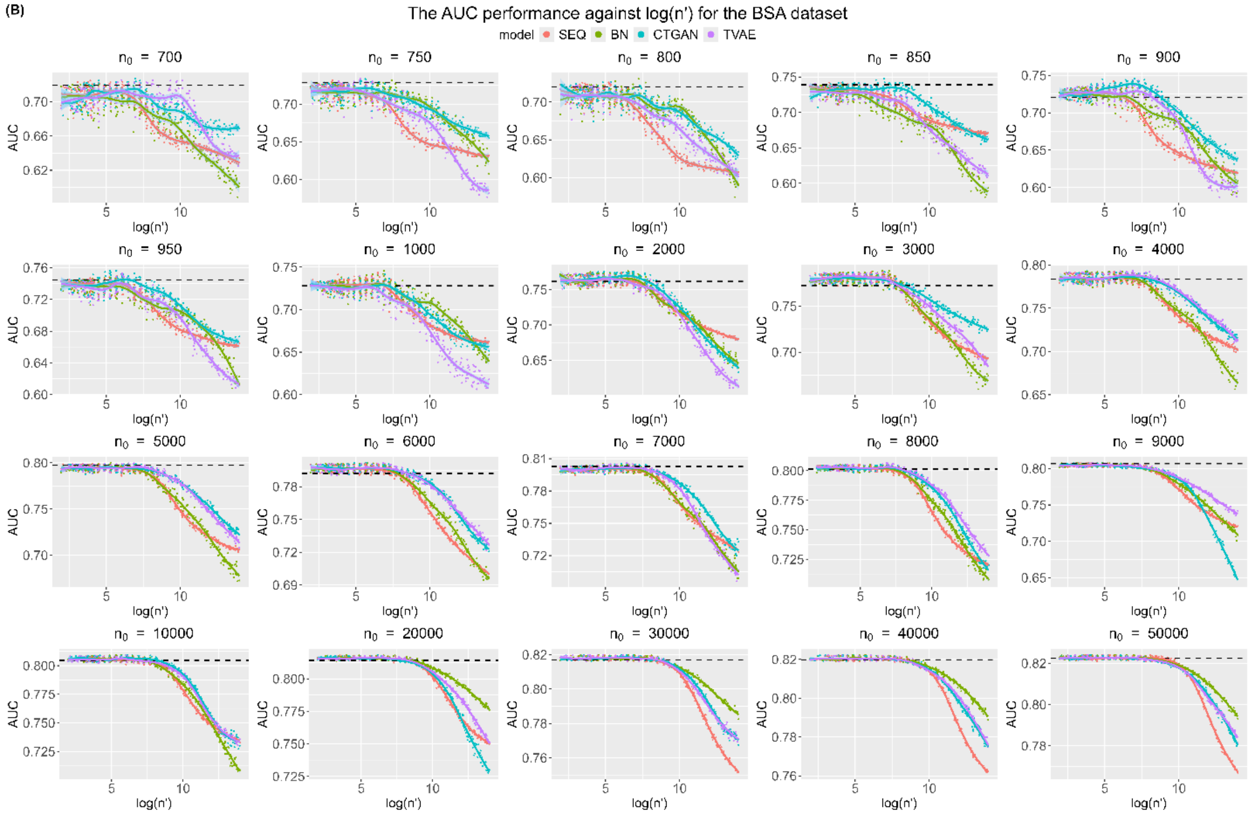

Figure B.4: Augmentation performance of ROC-AUC against log(n') for the BSA dataset (B).

B.3 California Dataset

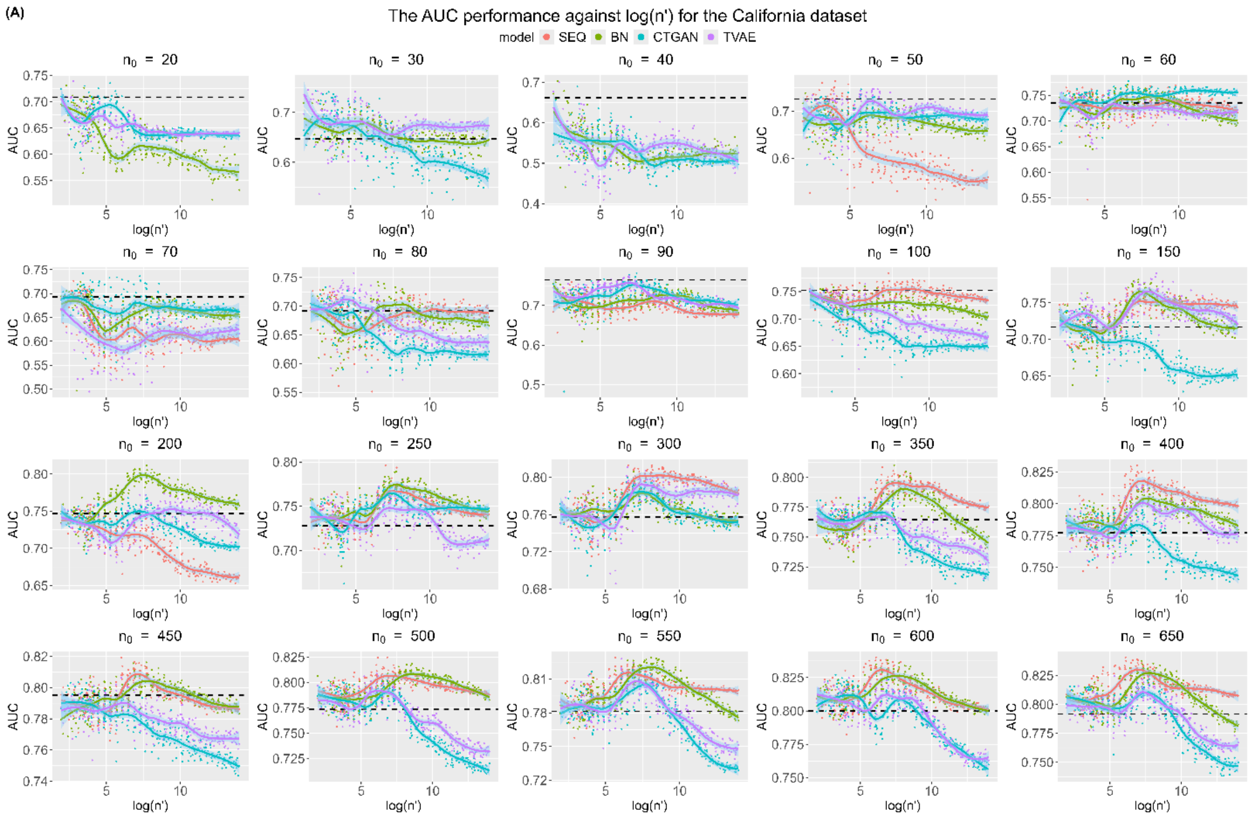

Figure B.5: Augmentation performance of ROC-AUC against log(n') for the California dataset (A).

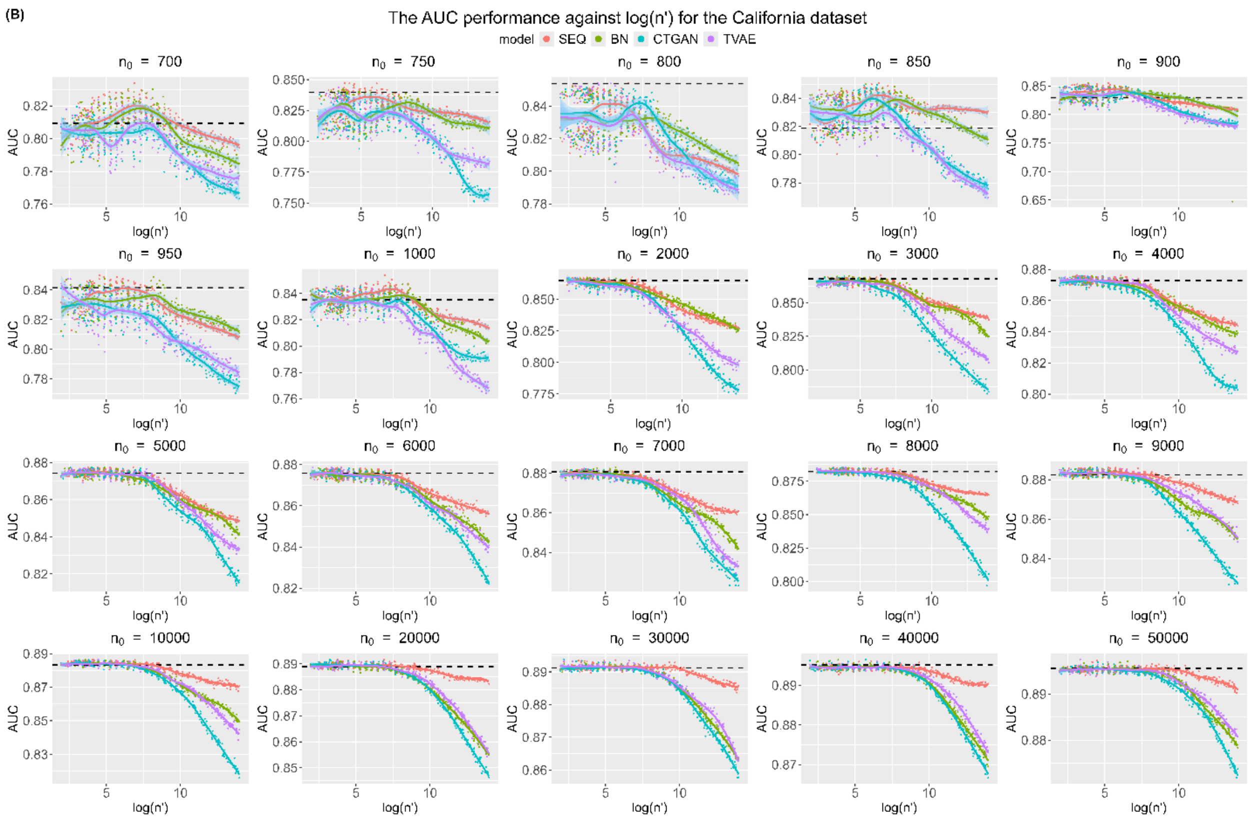

Figure B.6: Augmentation performance of ROC-AUC against log(n') for the California dataset (B).

B.4 CCHS Dataset

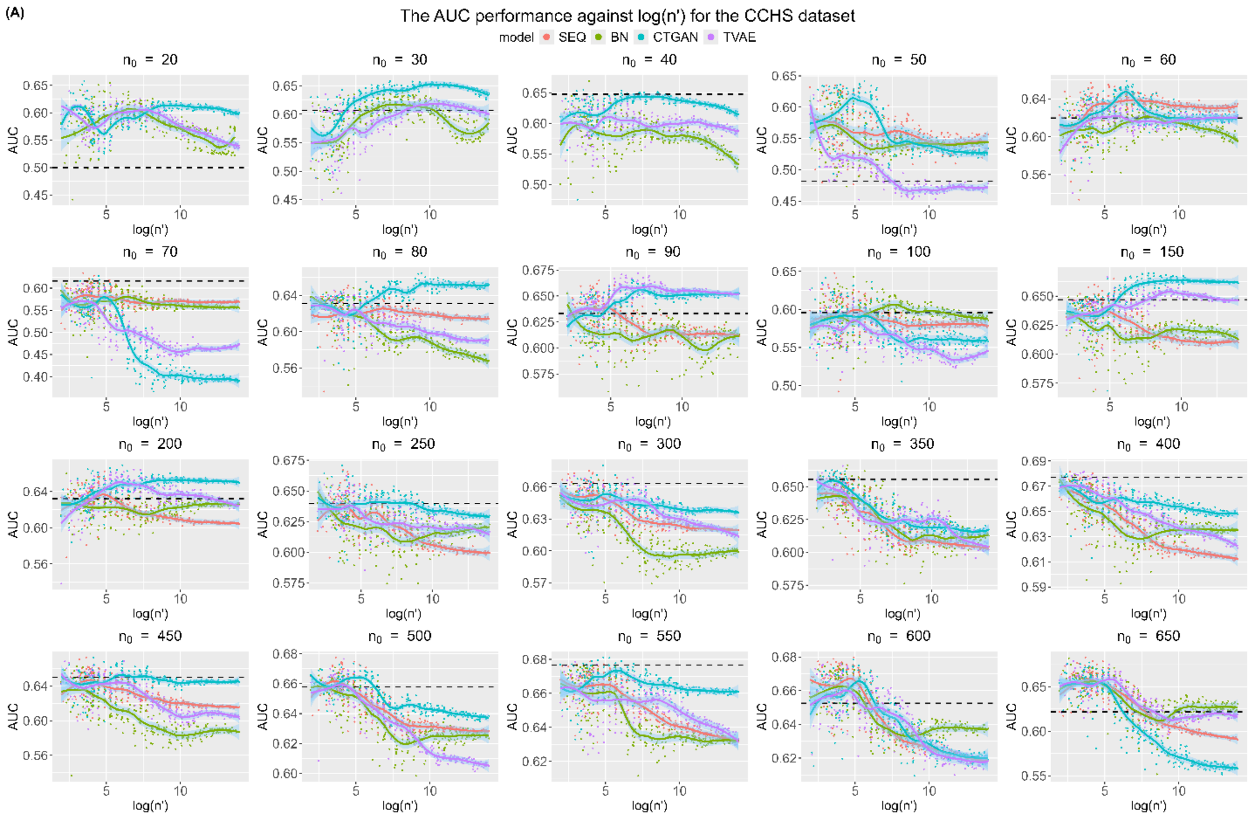

Figure B.7: Augmentation performance of ROC-AUC against log(n') for the CCHS dataset (A).

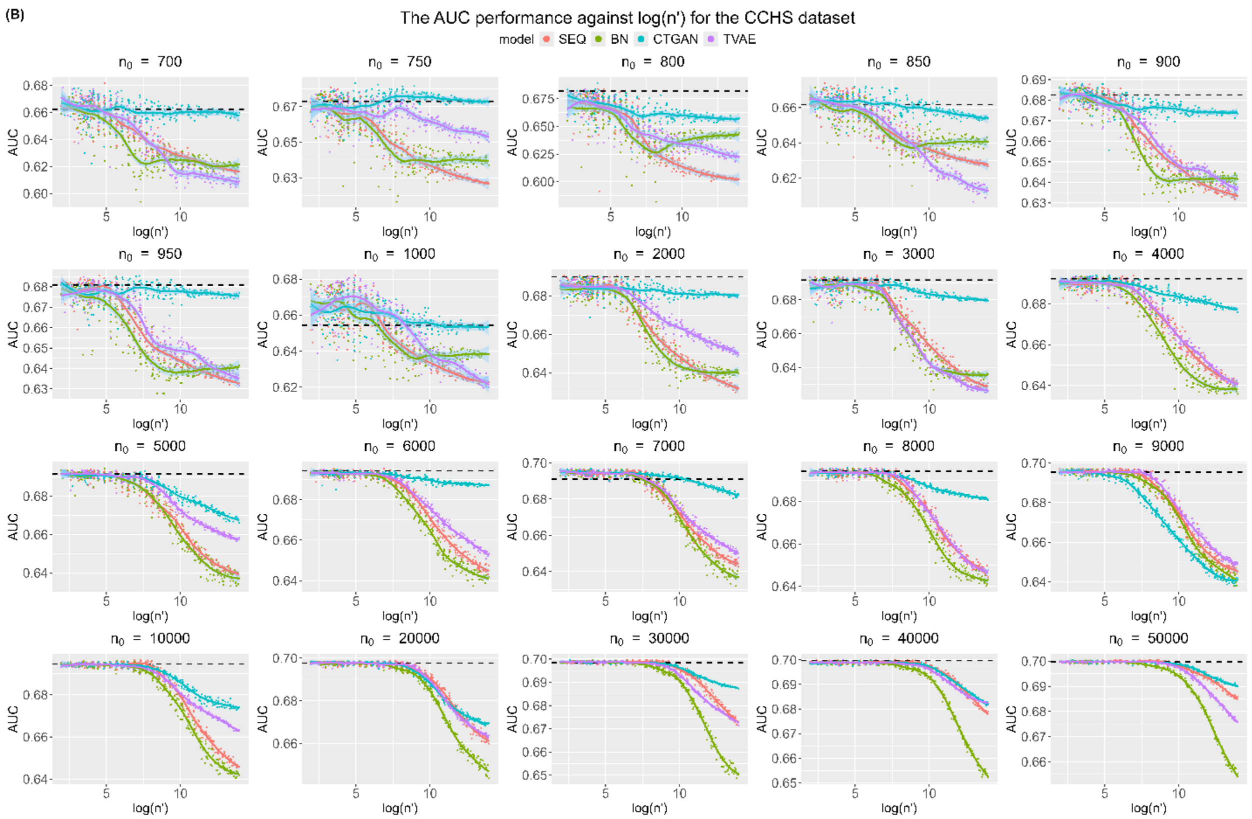

Figure B.8: Augmentation performance of ROC-AUC against log(n') for the CCHS dataset (B).

B.5 COVID-19 Dataset

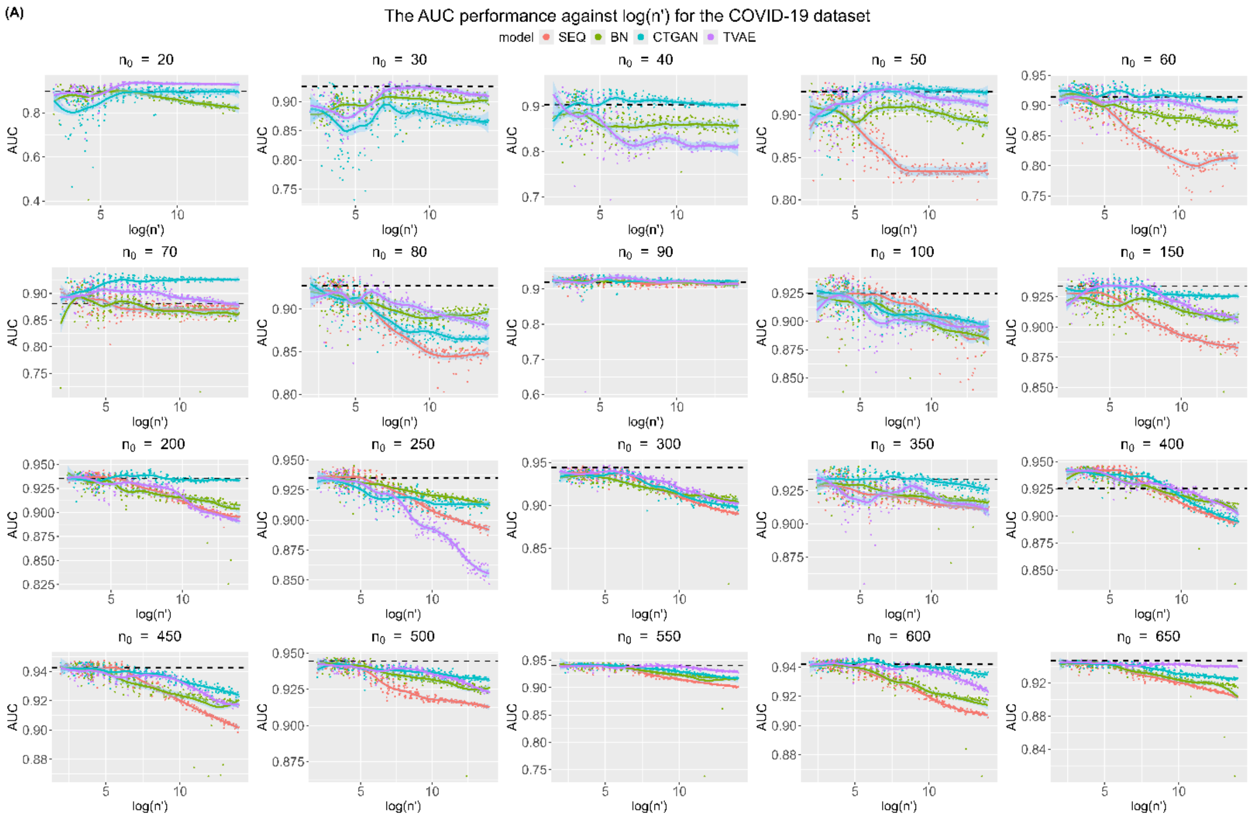

Figure B.9: Augmentation performance of ROC-AUC against log(n') for the COVID-19 dataset (A).

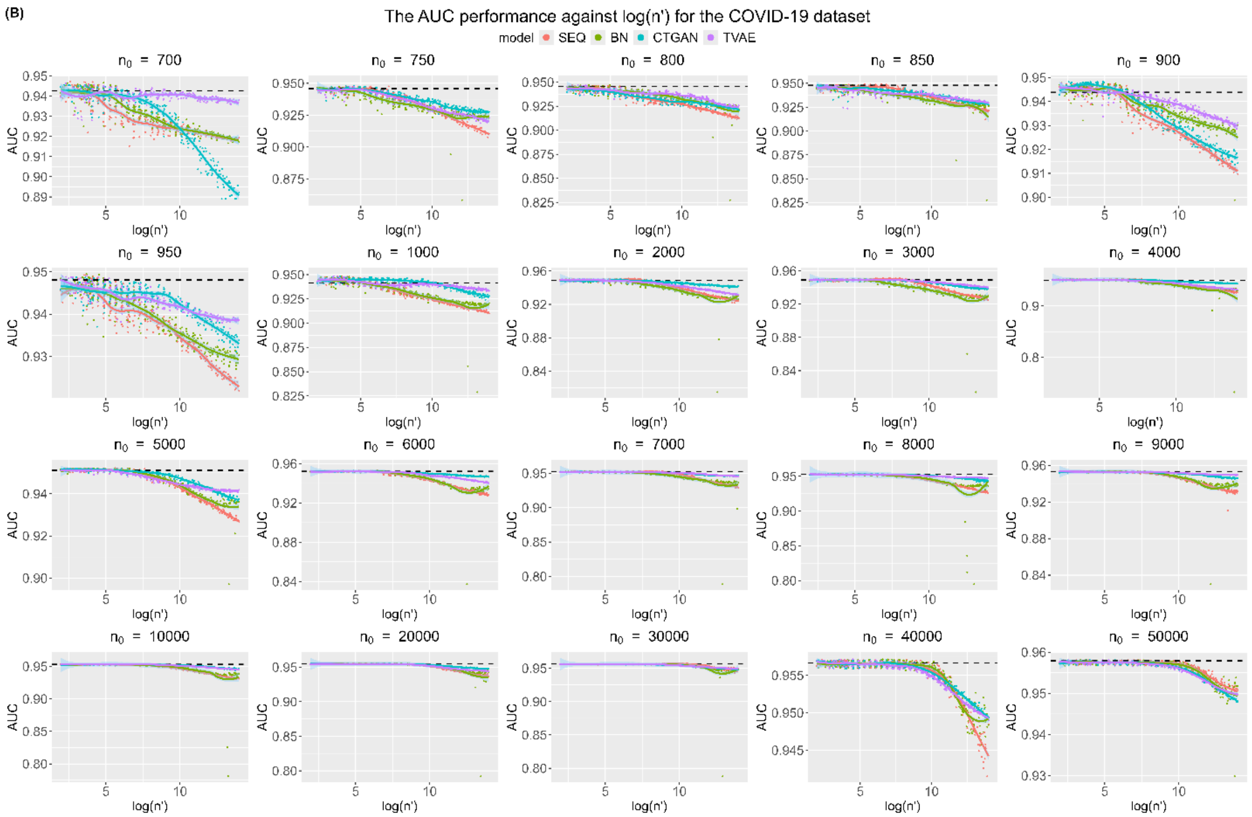

Figure B.10: Augmentation performance of ROC-AUC against log(*n'*) for the COVID-19 dataset (B).

B.6 FAERS Dataset

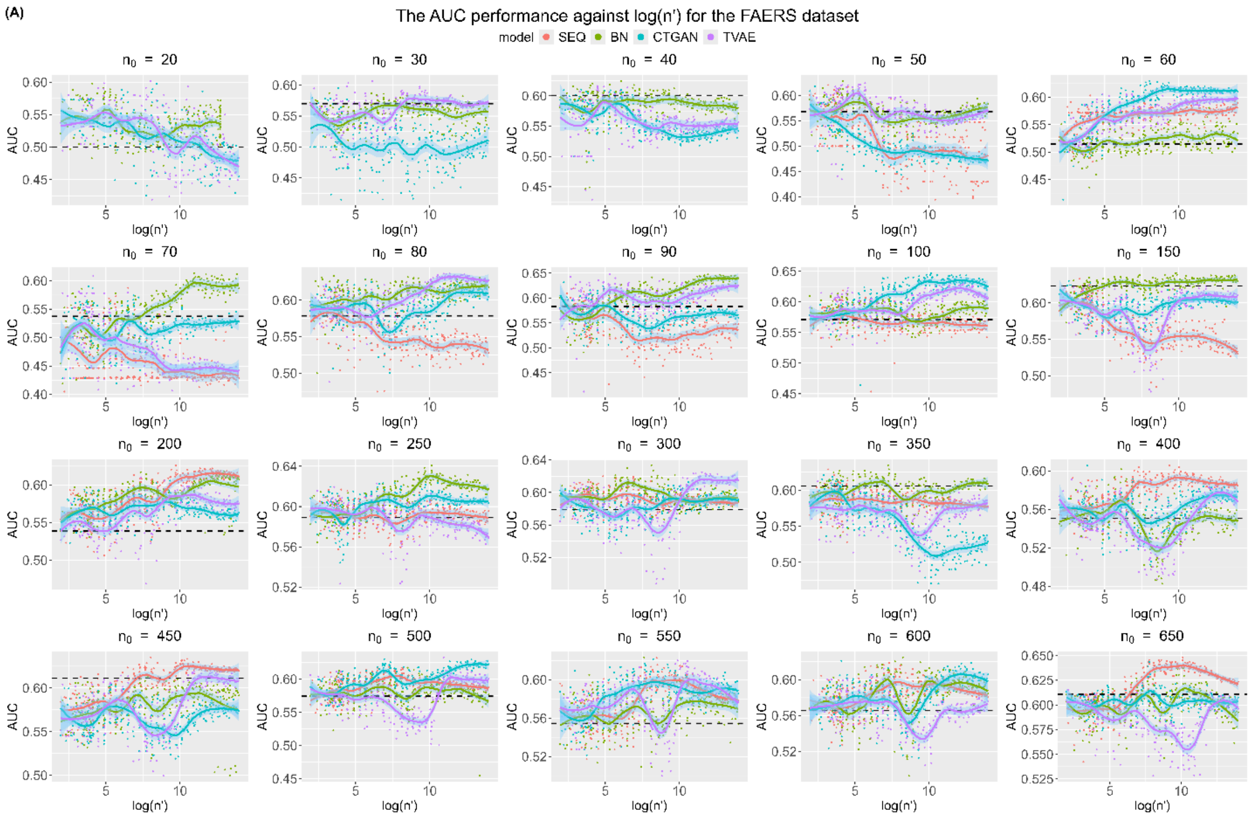

Figure B.11: Augmentation performance of ROC-AUC against log(n') for the FAERS dataset (A).

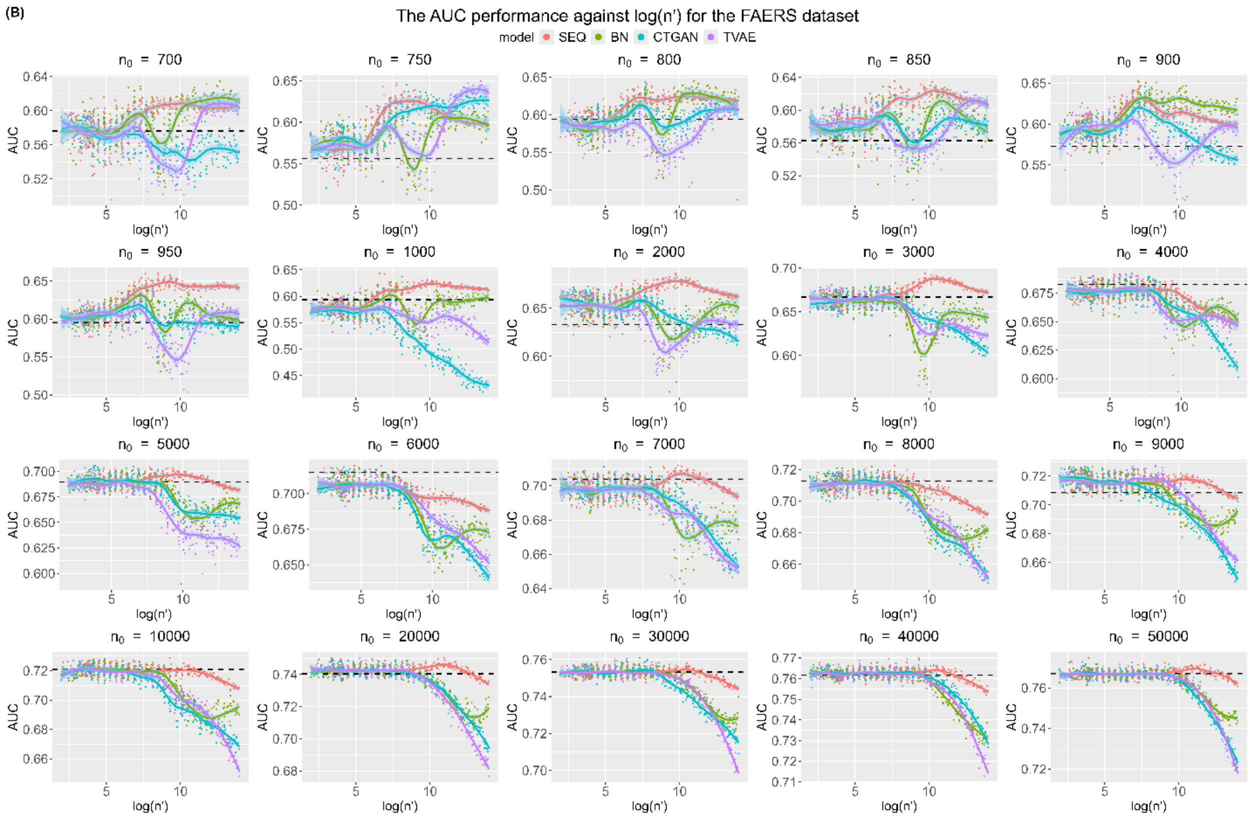

Figure B.12: Augmentation performance of ROC-AUC against log(*n'*) for the FAERS dataset (B).

B.7 Florida Dataset

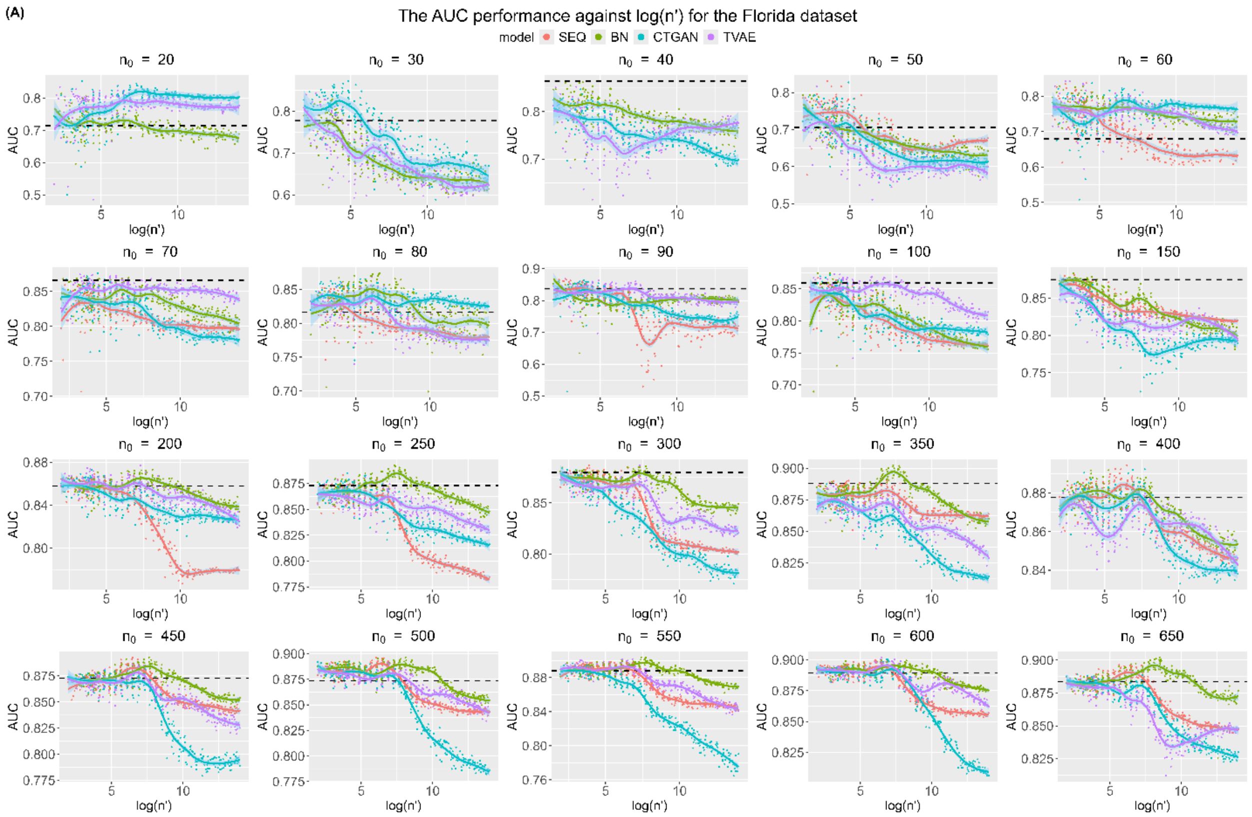

Figure B.13: Augmentation performance of ROC-AUC against log(n') for the Florida dataset (A).

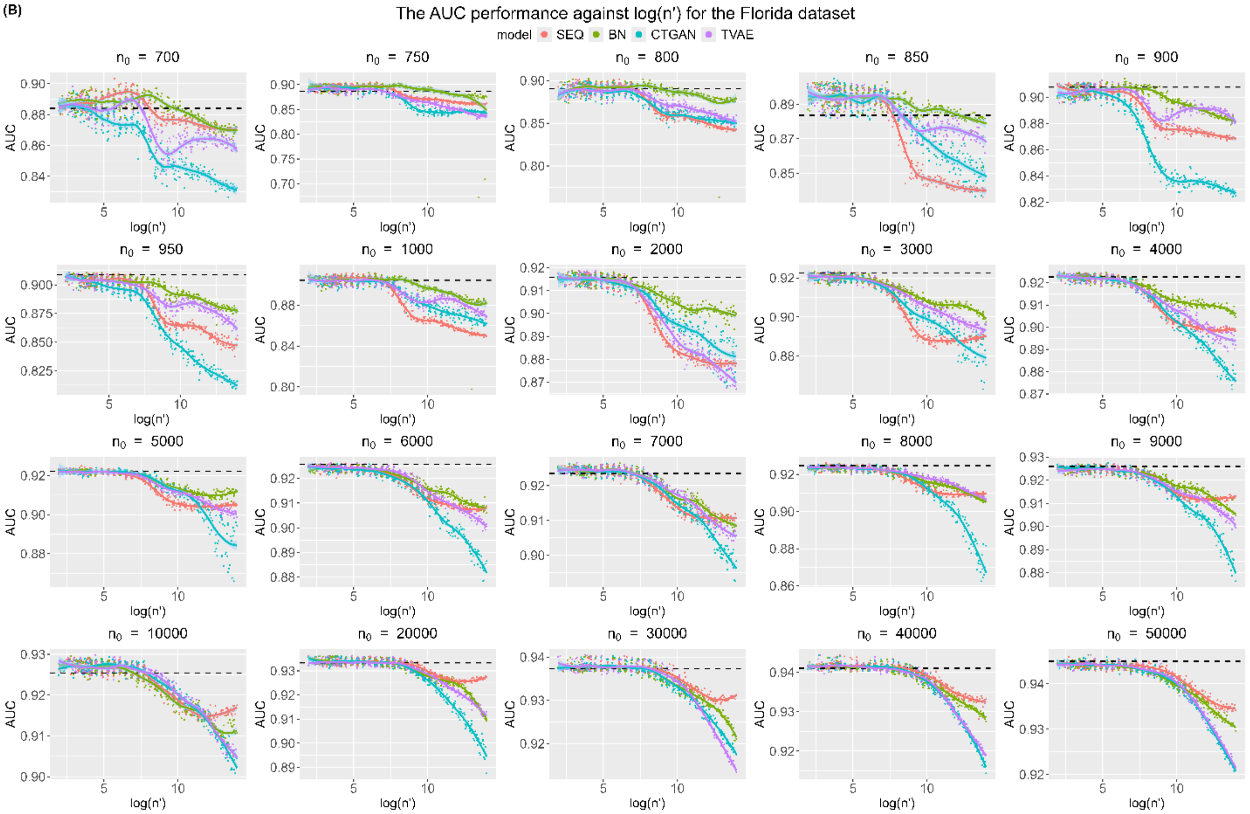

Figure B.14: Augmentation performance of ROC-AUC against log(n') for the Florida dataset (B).

B.8 MIMIC-III Dataset

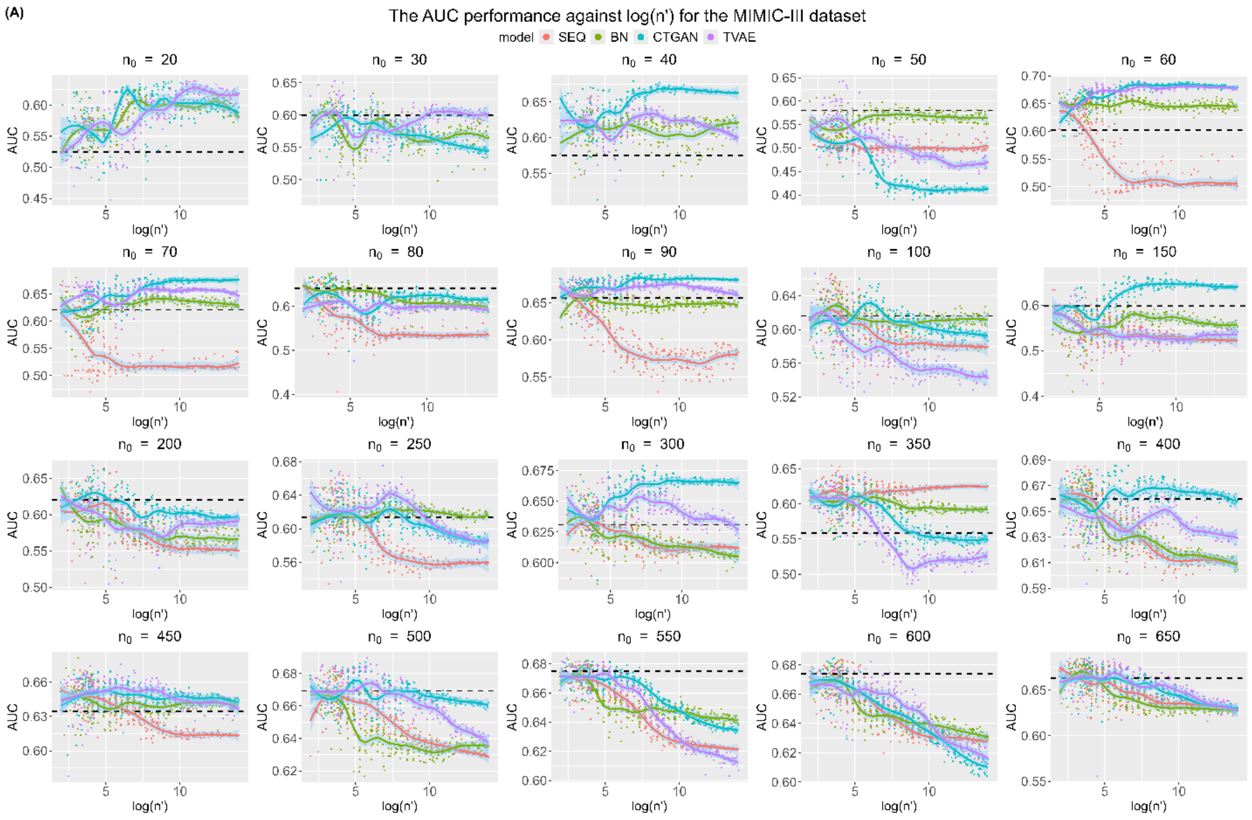

Figure B.15: Augmentation performance of ROC-AUC against log(n') for the MIMIC-III dataset (A).

(B)

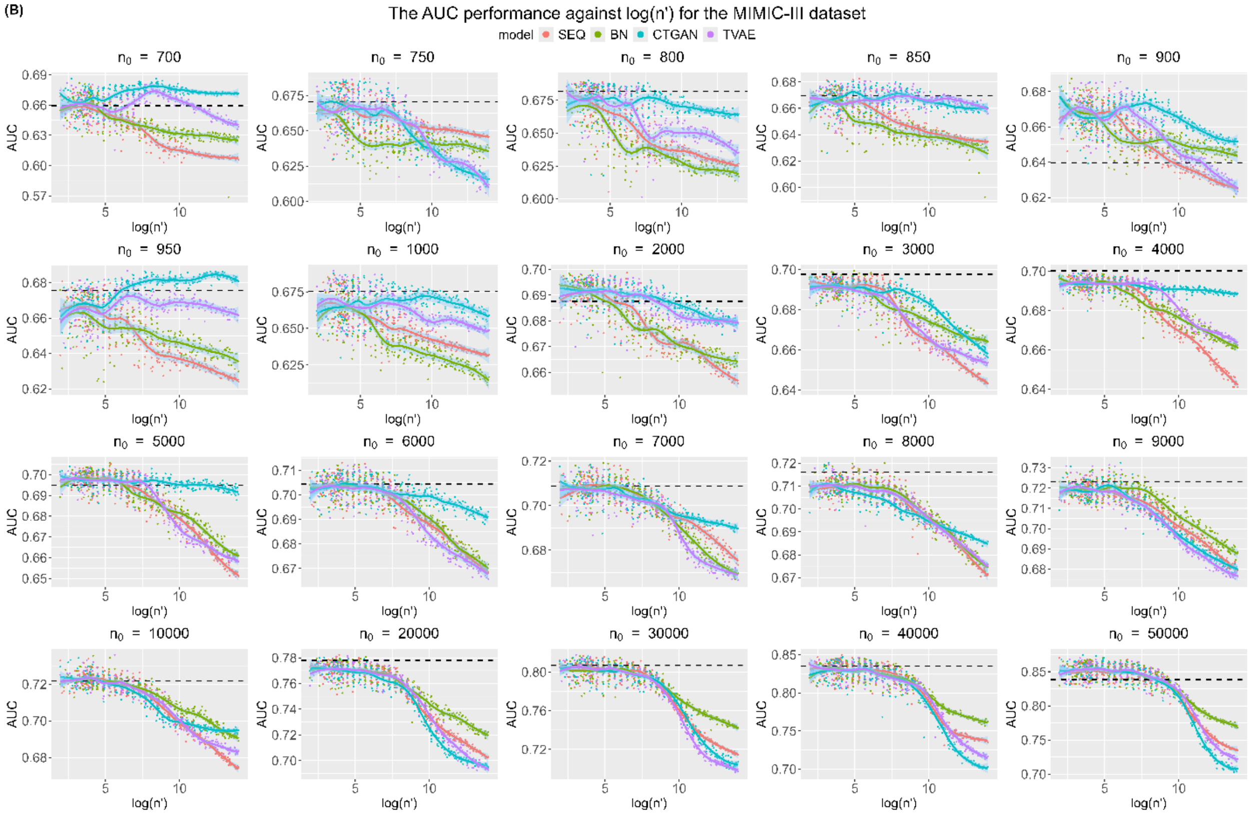

Figure B.16: Augmentation performance of ROC-AUC against log(n') for the MIMIC-III dataset (B).

B.9 New York Dataset

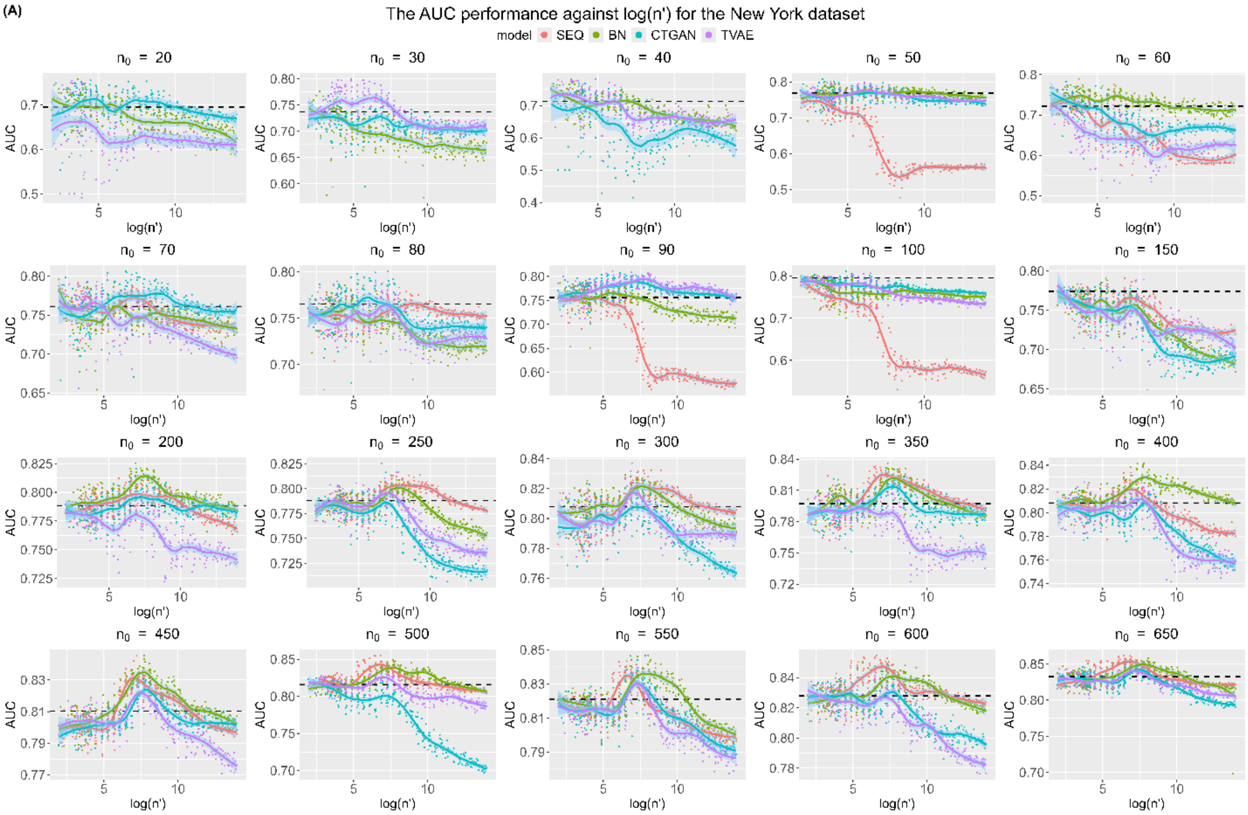

Figure B.17: Augmentation performance of ROC-AUC against log(n') for the New York dataset (A).

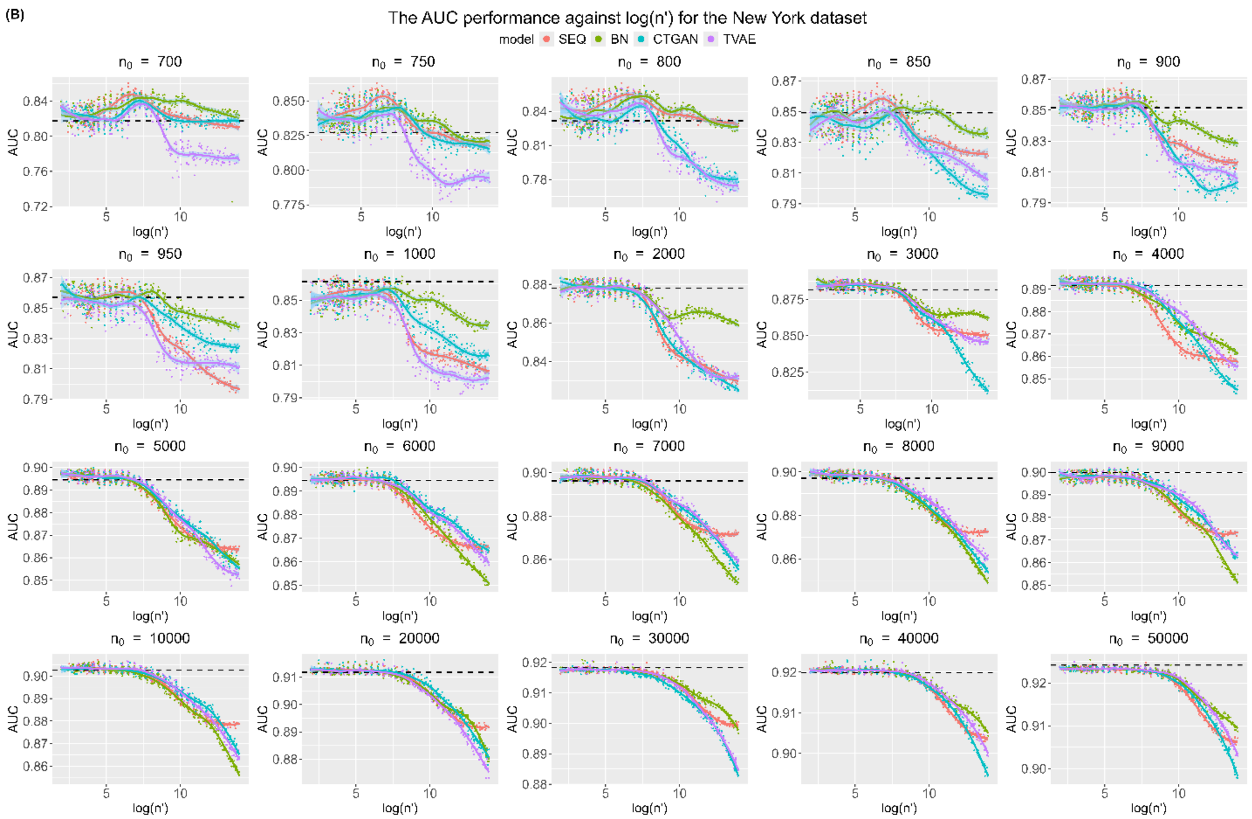

Figure B.18: Augmentation performance of ROC-AUC against log(n') for the New York dataset (B).

B.10 Nexoid Dataset

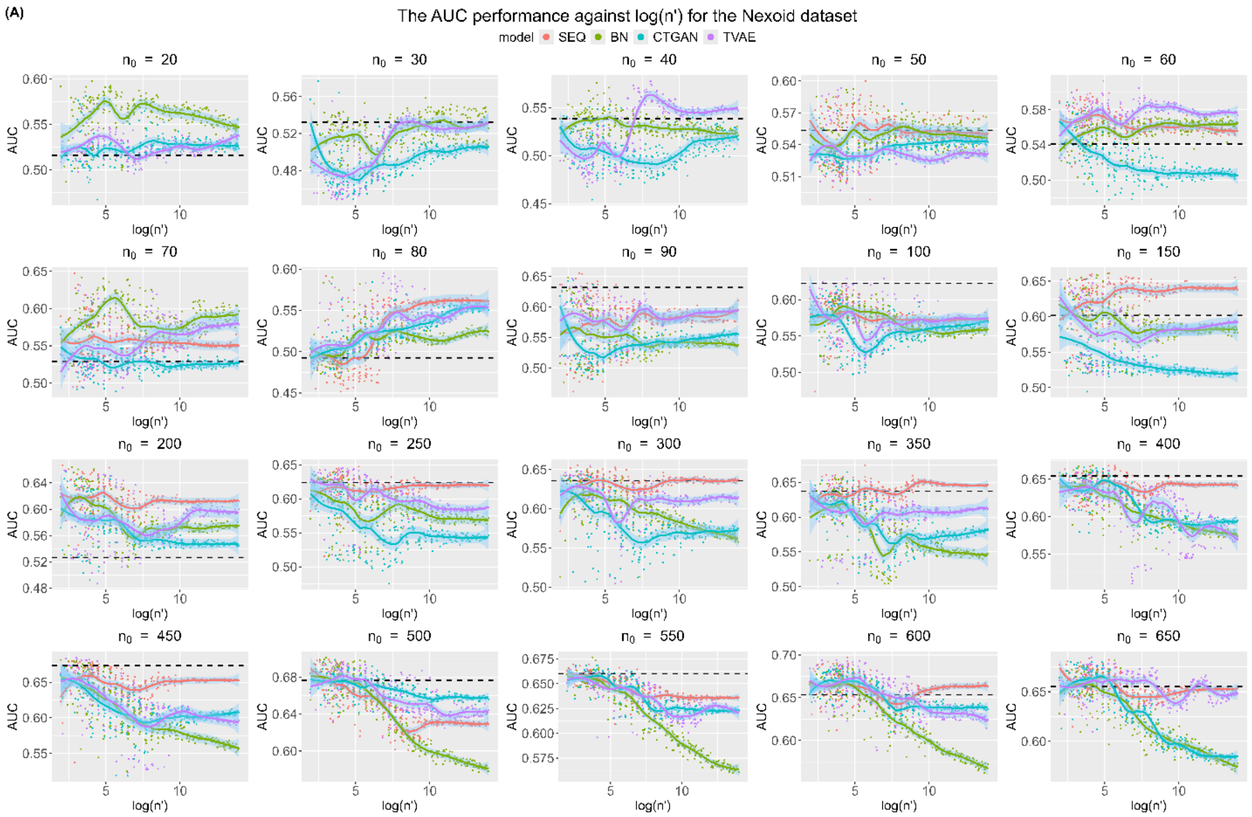

Figure B.19: Augmentation performance of ROC-AUC against log(n') for the Nexoid dataset (A).

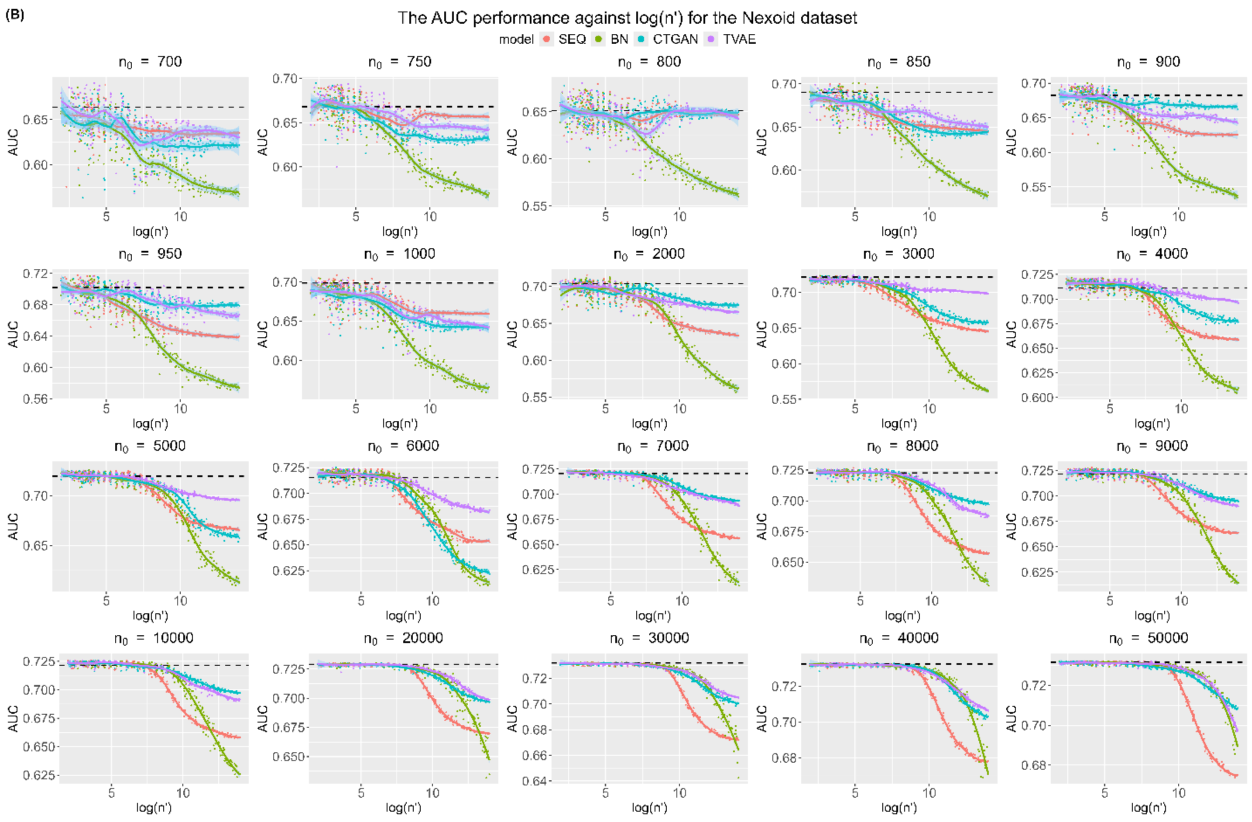

Figure B.20: Augmentation performance of ROC-AUC against log(n') for the Nexoid dataset (B).

B.11 Texas Dataset

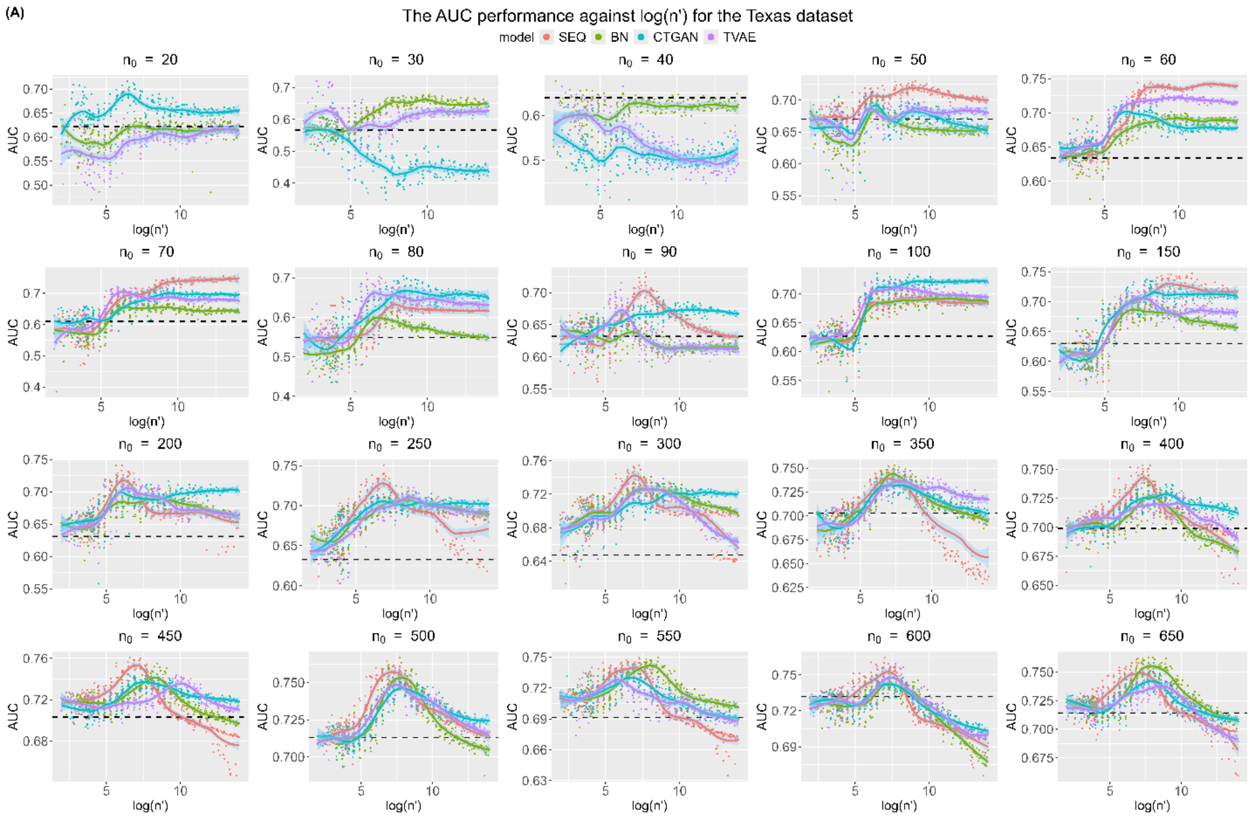

Figure B.21: Augmentation performance of ROC-AUC against log(n') for the Texas dataset (A).

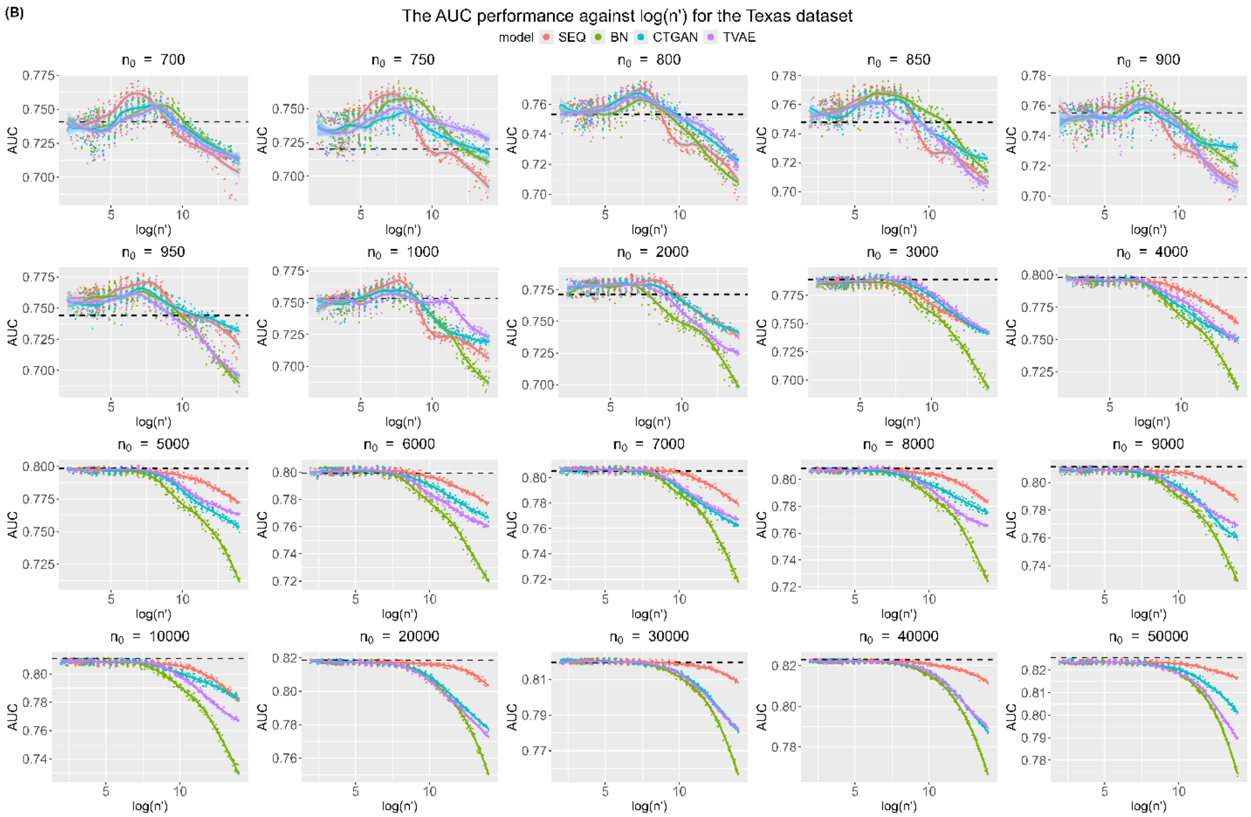

Figure B.22: Augmentation performance of ROC-AUC against log(n') for the Texas dataset (B).

B.12 Washington Dataset

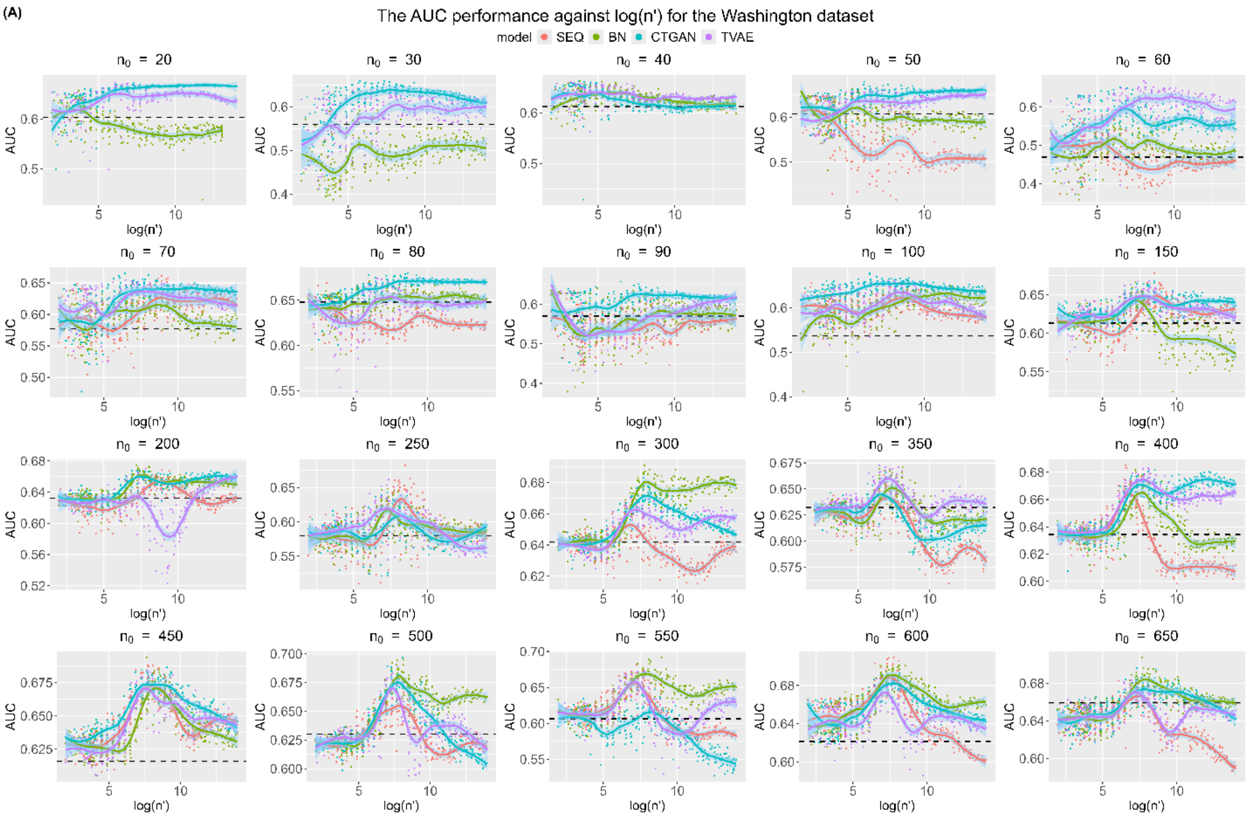

Figure B.23: Augmentation performance of ROC-AUC against log(n') for the Washington dataset (A).

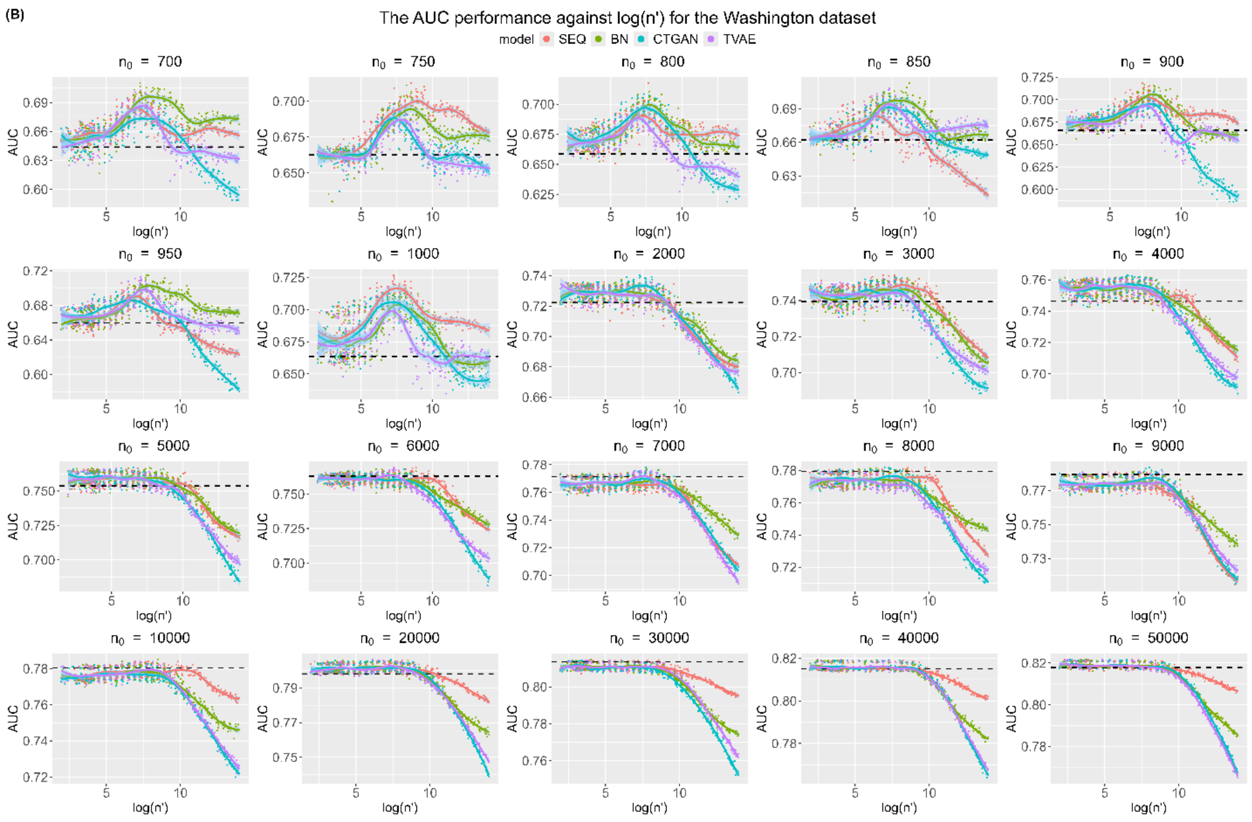

Figure B.24: Augmentation performance of ROC-AUC against log(n') for the Washington dataset (B).

B.13 Washington2008 Dataset

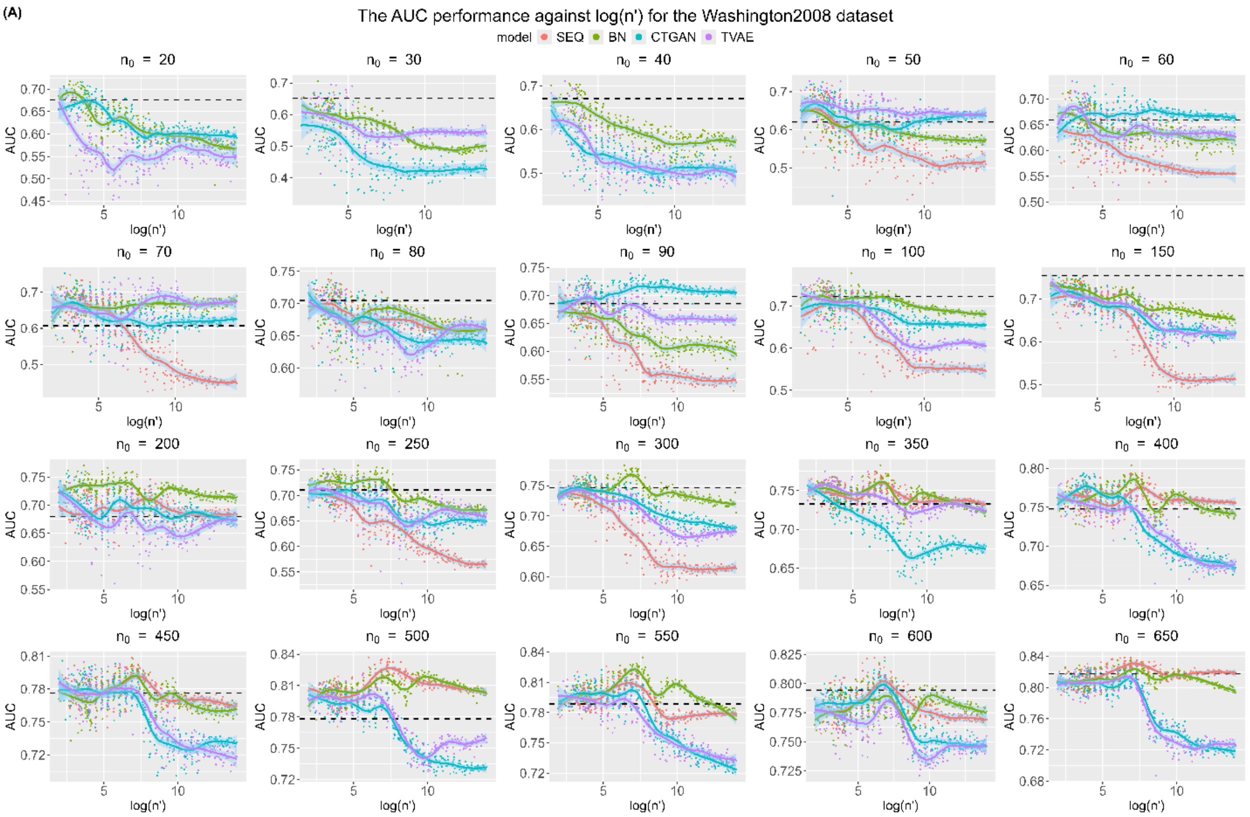

Figure B.25: Augmentation performance of ROC-AUC against log(n') for the Washington2008 dataset (A).

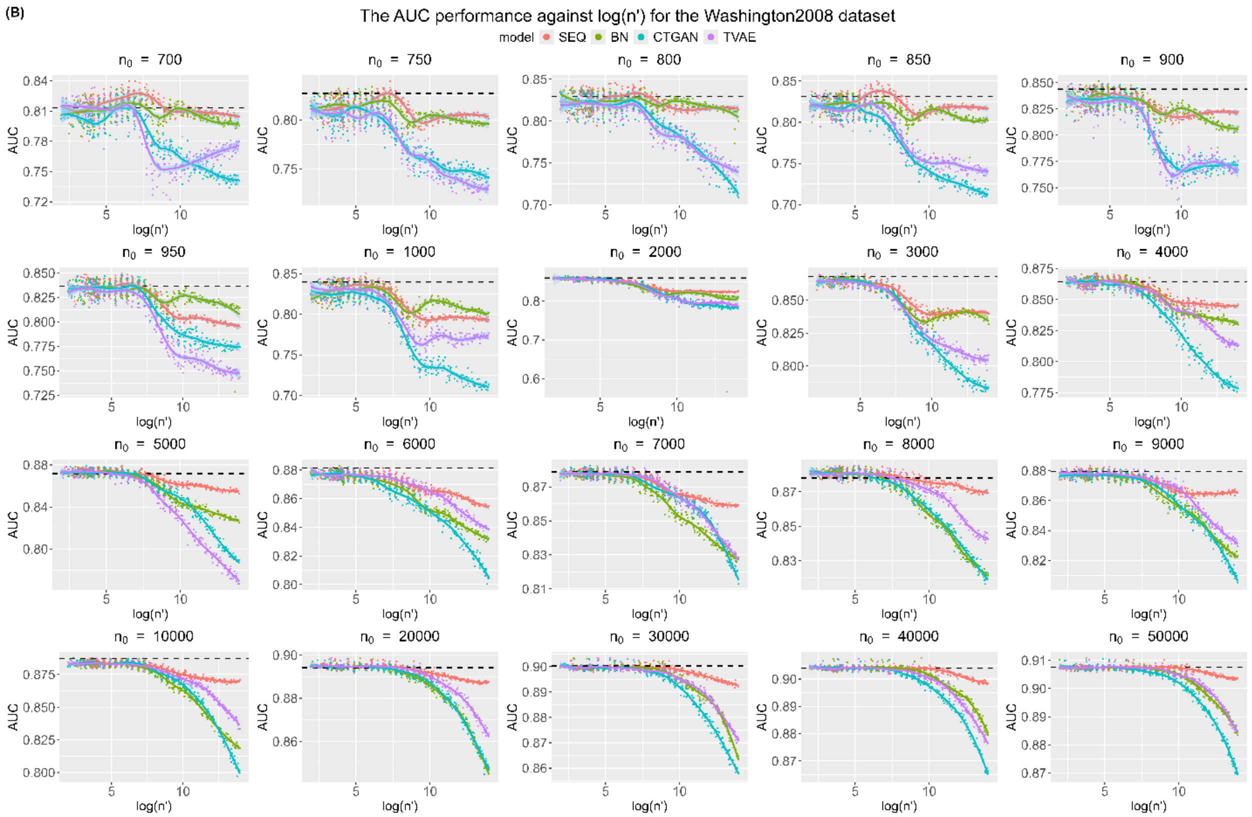

Figure B.26: Augmentation performance of ROC-AUC against log(n') for the Washington2008 dataset (B).

Appendix C - Results for case studies

C.1 Application to Breast Cancer Dataset

The first dataset to be analyzed is the Breast Cancer dataset that comprises the health information related to the recurrence of breast cancer, provided by the University Medical Centre, Institute of Oncology in Yugoslavia [1]. The original and preprocessed data share the same size of 277 records and dimension of 10 variables. The statistical descriptives of the variables for the dataset is summarized in Table E.14. We used this dataset to predict the recurrence of breast cancer based on its associated demographic and clinical factors.

Table C.1 displays the model performance from augmentation using the four generative models in terms of the number of synthetic observations that should be generated to achieve the optimal performance, baseline ROC-AUC, maximum ROC-AUC and maximum relative ROC-AUC in percentage.

Model	n'_{max}	Baseline ROC-AUC	Augmented ROC-AUC	Relative ROC-AUC (%)	Resampled ROC-AUC
SEQ	34	0.7143	0.7330	2.62	0.6500
BN	16	0.7143	0.7413	3.79	0.7060
**CTGAN**	**25**	**0.7143**	**0.7451**	**4.31**	**0.6729**
TVAE	21	0.7143	0.7406	3.69	0.7323

Table C.1: Analysis results of augmentation performance for the Breast Cancer dataset. n'_{max}: n' that leads to maximum ROC-AUC. Baseline ROC-AUC: baseline ROC-AUC from the base data. Augmented ROC-AUC: maximum ROC-AUC from the augmented data. Resampled ROC-AUC: ROC-AUC from the augmented data with a size of n'_{max} using the bootstrap method.

Analysis results show that the baseline ROC-AUC was 0.7143. Among the four generative models, CTGAN outperformed the other three in terms of the maximum ROC-AUC of 0.7451, which was acquired by simulating additional 25 observations, resulting in a relative change in ROC-AUC of 4.31%. Thus, the ML model performance was improved by 4.31% from augmenting the original data with extra 25 observations, compared to the baseline performance using the original data only. Moreover, the augmentation performance using bootstrap resampling is overall worse than using any generative model further spotlights the benefits of adding diverse information from the synthetic generative models. The fact that Table C.1 the augmentation performance using bootstrap resampling is even worse than the baseline performance further reveals that the bootstrap method might not be a good choice to enhance the model performance.

Similar to the previous case studies, the baseline ROC-AUC for this dataset would be considered moderate to good [2]. Therefore, at this level of performance augmentation does provide additional prognostic value.

C.2 Application to Breast Cancer Coimbra Dataset

The Breast Cancer Coimbra dataset contains a total of 116 women that were recruited and screened with breast cancer by the Gynaecology Department of the University Hospital Centre of Coimbra between 2009 and 2013 [3]. The data preprocessing results in 10 variables across 116 records, covering patient’s demographics, anthropometric measurements and information gathered by the routine blood tests. Table E.15 displays the detailed description of the chosen variables in the analysis. After computing

the data characteristics for the Breast Cancer Coimbra analysis dataset, our decision model suggests that augmentation should be performed. We are building an LGBM model to predict the presence of breast cancer using age, body mass index, glucose, insulin, homeostasis model assessment, leptin, adiponectin, resistin, and MCP-1.

Model	n'_{max}	Baseline ROC-AUC	Augmented ROC-AUC	Relative ROC-AUC (%)	Resampled ROC-AUC
SEQ	32	0.7392	0.8218	11.18	0.7599
**BN**	**53**	**0.7392**	**0.8722**	**18.00**	**0.8291**
CTGAN	71	0.7392	0.8416	13.85	0.7747
TVAE	94	0.7392	0.8490	14.86	0.7621

Table C.2: Analysis results of augmentation performance for the Breast Cancer Coimbra dataset. n'_{max}: n' that leads to maximum ROC-AUC. Baseline ROC-AUC: baseline ROC-AUC from the base data. Augmented ROC-AUC: maximum ROC-AUC from the augmented data. Resampled ROC-AUC: ROC-AUC from the augmented data with a size of n'_{max} using the bootstrap method.

The augmentation results for the Breast Cancer Coimbra dataset are summarized in Table C.2. The optimal performance was achieved by generating 53 more observations using Bayesian networks, leading to the maximum ROC-AUC value of 0.8722, compared to the baseline ROC-AUC of 0.7392. That leads to a remarkable increase in the relative ROC-AUC of 18.00%. In comparison, the performance of using bootstrap method to augment the original data is conspicuously much worse with the ROC-AUC being 0.8291. It demonstrates the value of incorporating diverse records rather than simply duplicating the records and the worth of using generative models to augment the data.

Similar to the previous case studies, the baseline ROC-AUC for this dataset would be considered moderate to good [2]. Therefore, at this level of performance augmentation does provide remarkable additional prognostic value.

C.3 Application to Colposcopy/Schiller Dataset

The Colposcopy/Schiller dataset is one of the three modality colposcopy data that examines the subjective quality assessment of digital colposcopies collected by Hospital Universitario de Caracas [4]. The dataset contains 287 observations with one target variable and 62 variables as predictors related to color information, image area and coverage in digital cervical imaging. The target variable is considered as the outcome in the analysis of whether the subjective judgment is good or bad. A detailed description of the outcome and remaining variables is reported in Table E.16. The LGBM model was used to predict the subjective quality assessment based on the sixty-two imaging variables.

Model	n'_{max}	Baseline ROC-AUC	Augmented ROC-AUC	Relative ROC-AUC (%)	Resampled ROC-AUC
SEQ	11	0.5125	0.6483	26.49	0.5454
BN	44	0.5125	0.6477	26.37	0.5147
**CTGAN**	**2205**	**0.5125**	**0.7341**	**43.23**	**0.6116**
TVAE	38	0.5125	0.6572	28.22	0.5628

Table C.3: Analysis results of augmentation performance for the Colposcopy/Schiller dataset. n'_{max}: n' that leads to maximum ROC-AUC. Baseline ROC-AUC: baseline ROC-AUC from the base data. Augmented ROC-AUC: maximum ROC-AUC from the augmented data. Resampled ROC-AUC: ROC-AUC from the augmented data with a size of n'_{max} using the bootstrap method.

The results are presented in Table C.3. The baseline performance yielded an ROC-AUC value of 0.5125. The best performance was achieved when the CTGAN was used to generate another set of 2,205 observations, resulting in an augmented ROC-AUC of 0.7341 and a substantial percentage improvement of 43.23% in the model performance. Moreover, the generative models also outperformed the bootstrap method. It reveals again that increasing the sample size without diversifying the dataset is less beneficial than increasing both the data size and diversity at the same time.

Similar to the previous case studies, the baseline ROC-AUC for this dataset would be considered poor [2]. Therefore, at this level of performance augmentation does provide pronounced additional prognostic value.

C.4 Application to Danish Colorectal Cancer Group Dataset

The Danish Colorectal Cancer Group (DCCG) database registered all patients in Denmark who were diagnosed with colorectal cancer or treated in a public Danish hospital since 2001 [5]. Patient data was acquired between 2001 and 2018 from the database, covering 12,855 clinical, surgical, radiological, and pathological records. After data preprocessing and removing the missingness in the postoperative medical complication, a total of 7,948 patient records remained, from which a random sample of 700 observations was drawn. In this analysis, we were interested in constructing a model to predict the probability of getting a postoperative medical complication based on associated risk factors, including age, gender, ASA score (i.e., pre-operative fitness score), localization of the tumor, procedure, pathological tumor (T) stage and node (N) stage, number of removed lymph nodes and number of lymph nodes with metastasis and unplanned intraoperative adverse event. A summary of statistics for the DCCG variables is presented in Table E.17. Similar to the Hot Flashes dataset, nested cross-validation was used to tune and evaluate an LGBM model.

Model	n'_{max}	Baseline ROC-AUC	Augmented ROC-AUC	Relative ROC-AUC (%)	Resampled ROC-AUC
SEQ	8	0.7171	0.7630	6.41	0.7344
BN	26	0.7171	0.7583	5.75	0.7405
CTGAN	820	0.7171	0.7768	8.32	0.6995
**TVAE**	**720**	**0.7171**	**0.7780**	**8.50**	**0.7077**

Table C.4: Analysis results of augmentation performance for the Danish Colorectal Cancer Group dataset. n'_{max}: n' that leads to maximum ROC-AUC. Baseline ROC-AUC: baseline ROC-AUC from the base data. Augmented ROC-AUC: maximum ROC-AUC from the augmented data. Resampled ROC-AUC: ROC-AUC from the augmented data with a size of n'_{max} using the bootstrap method.

Table C.4 summarizes the results for the DCCG dataset. Given the characteristics of the DCCG dataset, data augmentation was again recommended. It was found that by simulating another 720 synthetic observations from the TVAE generator on top of the original 700 observations, the optimal performance reaches an ROC-AUC of 0.7780 from the baseline ROC-AUC of 0.7171, leading to a relative ROC-AUC increase of 8.50%. In other words, the ML performance was enhanced from augmentation by 8.50% compared to the baseline performance without augmentation. Compared to TVAE, CTGAN performs similarly by improving its performance by up to 8.32%. Meanwhile, the performance of augmenting the analysis data to the same size n'_{max} using the bootstrap method is inferior to using the four generative models, which further highlights the value of making the dataset more diverse.

Similar to the previous case study, the baseline ROC-AUC for this dataset would be considered moderate to good [2]. Therefore, at this level of performance augmentation does provide additional prognostic value.

C.5 Application to Diabetic Retinopathy Dataset

The fifth dataset to be analyzed is the Diabetic Retinopathy dataset, which describes detections of diabetic retinopathy represented by either detected lesions or image-level descriptors from Messidor image set [6]. The total size of the original data is 1,151, from which a sample with a size of 600 was randomly selected. The detailed description of the analysis dataset is described in Table E.18. We built an LGBM prediction model to predict the signs of diabetic retinopathy using all the available imaging features.

Model	n'_{max}	Baseline ROC-AUC	Augmented ROC-AUC	Relative ROC-AUC (%)	Resampled ROC-AUC
SEQ	47	0.7400	0.7523	1.66	0.7281
**BN**	**11534**	**0.7400**	**0.7974**	**7.75**	**0.7299**
CTGAN	84	0.7400	0.7550	2.02	0.6943
TVAE	40737	0.7400	0.7600	2.70	0.7235

Table C.5: Analysis results of augmentation performance for the Diabetic Retinopathy dataset. n'_{max}: n' that leads to maximum ROC-AUC. Baseline ROC-AUC: baseline ROC-AUC from the base data. Augmented ROC-AUC: maximum ROC-AUC from the augmented data. Resampled ROC-AUC: ROC-AUC from the augmented data with a size of n'_{max} using the bootstrap method.

Table C.5 displays the augmentation performances using the four generative models and the bootstrap method. Given the baseline ROC-AUC is 0.7400, Bayesian networks led to the optimal performance with the maximum ROC-AUC being 0.7974 and the highest relative ROC-AUC increase of 7.75% when an additional 11,534 synthetic set was simulated and incorporated. On the other hand, the bootstrap method performed worse than the synthetic generative models and even worse than the baseline performance in most of the scenarios, which again shows the value of generating diverse data.

Similar to the previous case studies, the baseline ROC-AUC for this dataset would be considered moderate to good [2]. Therefore, at this level of performance augmentation does provide additional prognostic value.

C.6 Application to Hot Flashes Dataset

This dataset is a survey dataset that was collected from patients with early breast cancer to understand the frequency and severity of vasomotor symptoms (VMS) and the effectiveness of previously applied interventions between June 5, 2020 and March 5, 2021 at two cancer in Ontario [7]. The original dataset contains 373 health records related to demographics, menopausal status, cancer-associated symptoms and treatments. The outcome is a binary variable that indicates whether the severity of the VMS problem is high or not, and the other seventeen variables were chosen as predictors, with detailed descriptions of these variables summarized in Table E.19. After preprocessing and removing the missing values in the outcome, the number of observations was reduced to 360. An LGBM model was built to predict the probability of severe VMS based on the relevant predictors.

**Model**	n'_{max}	**Baseline ROC-AUC**	**Augmented ROC-AUC**	**Relative ROC-AUC (%)**	**Resampled ROC-AUC**
SEQ	25	0.7161	0.7488	4.57	0.7312
BN	198	0.7161	0.7497	4.69	0.6611
**CTGAN**	**720**	**0.7161**	**0.7668**	**7.08**	**0.6477**
TVAE	278	0.7161	0.7573	5.75	0.6783

Table C.6: Analysis results of augmentation performance for the Hot Flashes dataset. n'_{max}: n' that leads to maximum ROC-AUC. Baseline ROC-AUC: baseline ROC-AUC from the base data. Augmented ROC-AUC: maximum ROC-AUC from the augmented data. Resampled ROC-AUC: ROC-AUC from the augmented data with a size of n'_{max} using the bootstrap method.

The results show that augmentation has significantly improved the model performance as the data were augmented. In addition, CTGAN outperforms the other three models by boosting the ML performance by 7.08% with the baseline and maximum ROC-AUC values being 0.7161 and 0.7668 respectively, when 720 observations are simulated and added to the 360 observations, giving a total dataset of 1080 observations. The augmentation performance of bootstrap is overall shown to be worse than the four generative models, as the augmented ROC-AUC values are lower than the maximum ROC-AUC. It reveals that diversifying the dataset has greater benefits in improving the model performance than simply replicating the observations to increase the sample size.

The baseline ROC-AUC for this dataset would be considered moderate to good [2]. Therefore, at this level of baseline performance augmentation does provide additional prognostic value.

C.7 Application to Thoracic Surgery Dataset

The last case study uses the Thoracic Surgery dataset collected retrospectively by Wroclaw Thoracic Surgery Centre in Poland, which describes the post-operative life expectancy of lung cancer patients who underwent lung resections between 2007 and 2011 [8]. After data preprocessing, the analysis dataset remains 470 as the original data size, covering one outcome, 1-year survival, and sixteen variables related to demographics, clinical diagnostics, and post-operative complications. The characteristics of the variables are presented in Table E.20. The goal of this analysis is to predict the survival after 1-year period based on the associated factors.

Model	n'_{max}	Baseline ROC-AUC	Augmented ROC-AUC	Relative ROC-AUC (%)	Resampled ROC-AUC
SEQ	46	0.5584	0.6151	10.14	0.5498
BN	3144	0.5584	0.6668	19.41	0.6731
CTGAN	1028	0.5584	0.6380	14.25	0.6446
**TVAE**	**6602**	**0.5584**	**0.6700**	**19.98**	**0.6914**

Table C.7: Analysis results of augmentation performance for the Thoracic Surgery dataset. n'_{max}: n' that leads to maximum ROC-AUC. Baseline ROC-AUC: baseline ROC-AUC from the base data. Augmented ROC-AUC: maximum ROC-AUC from the augmented data. Resampled ROC-AUC: ROC-AUC from the augmented data with a size of n'_{max} using the bootstrap method.

The analysis results are summarized in Table C.7. TVAE turns out to be the best generative model to achieve the optimal augmentation performance for the Thoracic Surgery dataset. The maximum ROC-AUC that TVAE can achieve is 0.6700, which is approximately 19.98% higher than the baseline ROC-AUC of 0.5584. In other words, the ML model performance was substantially enhanced by 19.98% from augmenting the original data, relative to the baseline performance using the original data only. However, the bootstrap performed slightly better than the generative models for this dataset.

Similar to the previous case studies, the baseline ROC-AUC for this dataset would be considered poor [2]. Therefore, at this level of performance augmentation does provide significant additional prognostic value.

Appendix D - Hyperparameters

The following are the hyperparameters, their default values, and range for tuning the ensemble models.

LGBM				
**Hyperparameter**	**Default**	**Lower bound**	**Upper bound**	**Transform**
booster	1 (gbdt)	1 (gbdt)	2 (goss)	2^learning_rate
max_depth	6	1	15	
learning_rate	log2 (0.3)	-10	0	
early_stopping_rounds	7	7	30	
min_data_in_leaf	10	1	60	
num_leaves	15	4	60	
**Random forest**				
**Hyperparameter**	**Default**	**Lower bound**	**Upper bound**	**Transform**
num.trees	500	1	2000	round(n^min.node.size), where n is the number of observations
min.node.size	0.5	0	1	
max.depth	15	1	50	
min.bucket	10	1	60	
**XGBoost**				
**Hyperparameter**	**Default**	**Lower bound**	**Upper bound**	**Transform**
gamma	0	-15	3	2^gamma
eta	log2(0.3)	-10	0	2^eta
max_depth	6	1	15	
early_stopping_rounds	7	7	30	
max_leaves	15	4	60	
min_child_weight	1	0	7	2^min_child_weight

Table D.1: The default values and ranges of hyperparameters in the models.

Appendix E – Details of the datasets

The following are the details for the preprocessed datasets that were used prior to model training in this study.

E.1 Better Outcomes Registry & Network

Data are collected from the BORN Ontario birth registry that covers about 1 million records regarding Ontario's maternal demographic characteristics, obstetrical history, health behaviors, prenatal screening and newborn care information. We combine the pregnancy and infant datasets and examine the association between low birthweight and its related risk factors. The relevant factors include gestational age, maternal age, maternal body mass index, total number of pregnancies a mother has experienced, number of previous preterm pregnancies, number of previous abortions, maternal smoking status, alcohol exposure, prenatal screening, mental health concerns for addiction, anxiety, depression, maternal health conditions for diabetes and genetics and drug exposure to Cocaine, Hallucinogens and Opioids. We follow the definition of low birthweight[25] and classify the newborns whose birth weights are less than 2,500 grams as infants with low birthweight. A value of 1 is given for newborns with low birthweight and 0 otherwise. A summary of descriptive statistics for the variables is presented in Table E.1.

Variable	Description	Type	Mean (SD) or level count (% of total size) or number of categories	Missingness (% of the total size)
Birth weight	Whether a newborn baby has low birthweight (<2,500 grams)	Categorical	1: 7.01%<br>0: 92.99%	0.00%
Gestational age	Gestational age of a newborn baby	Numeric	1 < 34 weeks: 2.32%<br>2 34-36 weeks: 6.00%<br>3 37-38 weeks: 27.15%<br>4 39-41 weeks: 64.11%<br>5 >=42 weeks: 0.43%	0.00%
Maternal age	Maternal age in years at time of stillbirth or live birth	Numeric	1 <= 19: 2.15%<br>2 20-34: 74.12%<br>3 35-39: 19.31%<br>4 >=40: 4.36%	0.06%
Maternal BMI	Maternal pre-pregnancy body mass index	Numeric	1 <18.5: 4.53%<br>2 18.5-24.9: 43.44%<br>3 25-29.9: 20.22%<br>4 >=30: 15.86%	15.94%
Parity	Total number of pregnancies a mother has experienced	Numeric	0: 42.82%<br>1: 34.57%<br>2: 13.98%<br>3: 4.64%<br>>=4: 2.89%	1.10%
Preterm birth	Number of previous preterm pregnancies	Numeric	0: 93.57%<br>1: 4.41%<br>2: 0.71%<br>3: 0.13%<br>>=4: 0.05%	1.13%
Abortions	Number of previous abortions	Numeric	0: 65.77%<br>1: 20.85%<br>2: 7.25%<br>3: 2.50%<br>>=4: 1.50%	2.13%
Smoking	Maternal smoking status at time of admission	Categorical	Yes: 7.64%<br>No: 88.14%	4.21%

Alcohol	Alcohol exposure in pregnancy	Categorical	Yes: 2.23%<br>No: 92.54%	5.23%
Prenatal screening	Whether a mother has prenatal screening during pregnancy	Categorical	Yes: 66.30%<br>No: 33.70%	0.00%
Addiction	Mental health concern regarding addiction	Categorical	Yes: 0.60%<br>No: 93.72%	5.68%
Anxiety	Mental health concern regarding anxiety	Categorical	Yes: 8.99%<br>No: 85.33%	5.68%
Depression	Mental health concern regarding depression	Categorical	Yes: 7.58%<br>No: 86.74%	5.68%
Diabetes	Maternal health condition regarding diabetes	Categorical	Yes: 1.00%<br>No: 93.27%	5.73%
Genetics	Maternal health condition regarding genetics	Categorical	Yes: 0.00%<br>No: 94.26%	5.73%
Cocaine drug	Drug exposure to Cocaine in pregnancy	Categorical	Yes: 0.25%<br>No: 94.65%	5.10%
Hallucinogens drug	Drug exposure to Hallucinogens in pregnancy	Categorical	Yes: 0.02%<br>No: 94.88%	5.10%
Opioids drug	Drug exposure to Opioids in pregnancy	Categorical	Yes: 0.42%<br>No: 94.48%	5.10%

Note: SD: standard deviation

Table E.1: Descriptive statistics for the BORN dataset.

E.2 Basic Stand Alone Inpatient Claims

This dataset contains the claim-level information with each recording being an inpatient claim chosen from a 5% random sample of Medicare beneficiaries during 2008. In this study, we choose the variables including age, gender, DRG, ICD-9 primary procedure code, Medicare payment and the length of stay and explore the relationship between the length of stay and its relevant demographic and claim-related factors. The outcome is defined as a binary variable taking a value of 1 if the length of stay on the file is greater than or equal to 2.5 days, and 0 otherwise. **Table E.2** provides an overview of the detailed statistics for these variables.

Variable	Description	Type	Mean (SD) or 1 count (% of total size) or number of categories	Missingness (% of the total size)
Outcome	Whether the length of stay on a claim is greater than 2.5 days	Categorical	1: 42.65%<br>0: 57.35%	0.00%
Age	The beneficiary's age	Numeric	1 Under 65: 19.73%<br>2 65- 69: 13.19%<br>3 70-74: 14.65%<br>4 75-79: 15.55%<br>5 80-84: 16.10%<br>6 85 & older: 20.78%	0.00%
Gender	The beneficiary's gender	Categorical	1 Male: 43.88%<br>2 Female: 56.12%	0.00%
DRG	Diagnostic related groups to which a hospital claim belongs for prospective payment purposes	Categorical	311	0.00%
ICD-9	Primary procedure (primarily surgical procedures) performed during the inpatient stay	Categorical	86	47.00%
Payment	Quintile value (or code) to which the actual Medicare payment amount on the claim belongs	Categorical	1: 19.97%<br>2: 20.12%<br>3: 20.00%<br>4: 19.82%<br>5: 20.09%	0.00%

Note: SD: standard deviation

Table E.2: Descriptive statistics for the BSA dataset.

E.3 California Hospital Discharges

This dataset contains over 4 million inpatient discharge records in 2008 from community hospitals in California from State Inpatient Databases that are used to track the trends in healthcare utilization, access, charges, quality and outcomes in United States. We are interested in exploring the relationship between length of stay and its demographic and health factors. Specifically, the covariates of interest include age, female, race, aweekend, DRG, DX1, primary payer, total charges, chronic conditional indicators, and procedure classes for ICD-10-PCS procedure codes, comorbidity measures for alcohol abuse, depression, hypertension and obesity. The outcome is generated by dividing the patients into two groups based on the median of their length of stay. A value of 1 is assigned if the patient's length of stay is greater than or equal to 3 days and 0 otherwise. Detailed statistics of the variables are displayed in **Table E.3**.

Variable	Description	Type	Mean (SD) or level count (% of total size) or number of categorie	Missingness (% of the total size)
Outcome	Whether a patient's length of stay is greater than 3 days	Categorical	1: 54.10%<br>0: 45.90%	0.00%
AGE	Patient's age in years	Numeric	44.59 (28.58)	0.95%
FEMALE	Whether a patient's gender is female	Categorical	1 Female: 57.24%<br>0 Male: 39.85%	2.91%
RACE	Patient's race	Categorical	1 White: 46.72%<br>2 Black: 7.27%<br>3 Hispanic: 28.52%<br>4 Asian or Pacific Islander: 7.07%<br>5 Native American: 0.07%<br>6 Other: 2.04%	8.31%
AWEEKEND	Whether a patient's admission day is on a weekend	Categorical	1 Admitted Saturday - Sunday: 20.39%<br>0 Admitted Monday - Friday: 79.61%	0.00%
DRG	Diagnosis Related Group	Categorical	746	0.00%
DX1	ICD-9-CM Diagnosis	Categorical	8,548	0.00%
PAY1	Expected primary payer (Medicare, Medicaid, private insurances, etc.)	Categorical	1 Medicare: 31.13%<br>2 Medicaid: 25.57%<br>3 Private insurance: 34.79%<br>4 Self-pay: 3.41%<br>6 Other: 5.09%	0.02%
TOTCHG	Total charges	Numeric	45,065.28 (78,294.38)	12.00%
CHRON1	ICD-9-CM Chronic Condition Indicators	Categorical	1 Chronic condition: 34.22%<br>0 Non-chronic condition: 65.78%	0.00%
CHRONB1	Chronic Condition Indicators - body system	Categorical	19	0.00%
PCLASS1	Procedure Classes Refined for ICD-10-PCS procedure codes	Categorical	1 Minor diagnostic: 9.65%<br>2 Minor therapeutic: 28.12%<br>3 Major diagnostic: 0.46%	35.40%

			4 Major Therapeutic: 26.37%	
CM_ALCOH	AHRQ comorbidity measure for ICD-9-CM codes: alcohol abuse	Categorical	1 Comorbidity is present: 3.84%<br>0 Comorbidity is not present: 96.16%	0.00%
CM_DEPRE	AHRQ comorbidity measure for ICD-9-CM codes: depression	Categorical	1 Comorbidity is present: 5.80%<br>0 Comorbidity is not present: 94.20%	0.00%
CM_HTN_C	AHRQ comorbidity measure for ICD-9-CM codes: hypertension (combine uncomplicated and complicated)	Categorical	1 Comorbidity is present: 33.31%<br>0 Comorbidity is not present: 66.69%	0.00%
CM_OBESE	AHRQ comorbidity measure for ICD-9-CM codes: obesity	Categorical	1 Comorbidity is present: 7.23%<br>0 Comorbidity is not present: 92.77%	0.00%

Note: SD: standard deviation

Table E.3: Descriptive statistics for the hospital California dataset.

E.4 Canadian Community Health Survey

The CCHS data is a cross-sectional telephone survey administered by Statistics Canada that collects information on the health status, health care utilization and health determinants of Canadians. This dataset is a pooled version of survey data from 2001 to 2013, and the variables we are using are presented in Table E.4.

The model outcome is cardiovascular health and the covariates are age, sex, education, house income, household size, and immigration as predictors to predict the ideal state of cardiovascular health using variables from the dataset [9]. To assess cardiovascular health, we follow the definition of ideal cardiovascular health introduced by the American Heart Association to calculate the Cardiovascular Health in Ambulatory Care Research Team (CANHEART) health index score, which is determined by 7 health factors including smoking, obesity, hypertension, diabetes, physical activity, and fruit and vegetable consumption [10]. The final CANHEART index score ranges from 0 (worst) to 6 (best). The outcome is assigned to be 1 if the score is above 3 [11], which is considered to be an intermediate or ideal state of cardiovascular health and 0 otherwise.

Variable	Description	Type	Mean (SD) or level count (% of total size)	Missingness (% of the total size)
CANHEART	Whether a patient is in ideal cardiovascular health; this is a sum of the prior six variables.	Categorical	1 Ideal: 63.95%<br>0 Non-ideal: 36.05%	0.00%
Age	Patient's age in years	Numeric	47.24 (20.19)	0.00%
Sex	Patient's gender	Categorical	1 Male: 45.84%<br>2 Female: 54.16%	0.00%
Education	Patient's highest level of education	Categorical	1 < Secondary school graduate: 26.14%<br>2 Secondary school graduate: 16.93%<br>3 Some post-secondary education: 6.65%<br>4 Post-secondary certificate: 48.55%	1.72%
Marital status	Patient's marital status	Categorical	1 Married: 43.41%<br>2 Common-law: 8.12%<br>3 Widow/separation/divorce: 19.55%<br>4 Single/never married: 28.74%	0.17%
House income	Total household income from all sources	Numeric	57,603.42 (32,061.49)	9.59%
Household size	Size of entire household	Numeric	2.39 (1.23)	15.66%
Immigration	Whether a patient is an immigrant	Categorical	1 Yes: 13.86%<br>2 No: 84.33%	1.81%

Note: SD: standard deviation

Table E.4: Descriptive statistics for the CCHS dataset.

E.5 Canadian COVID-19

The first dataset is the Canadian COVID-19 dataset from the Public Health Agency of Canada. It contains over 1 million health records of individuals who have tested positive for COVID-19. We are interested in fitting a model that predicts mortality caused by COVID-19. The binary outcome of interest is derived from the case status in the dataset, and a value of 1 is assigned if the patient has died due to COVID-19 while a value 0 is assigned if the patient has recovered. The selected predictors for modeling include the following variables: date, age group, gender, region, exposure, province. **Table E.5** presents an overview of the variables that are included in the binary model.

Variable	Description	Type	Mean (SD) or level count (% of total size) or number of categories	Missingness (% of the total size)
Case status	The status of a patient	Categorical	1: 1.48 %<br>0: 98.52%	0.00%
Date	The date when a case is reported	Numeric (computed as the number of days since 1st January 2020)	348.07 (96.16)	0.00%
Age group	Patient's age group in years	Numeric	1 <20: 17.92%<br>2 20-29: 20.26%<br>3 30-39: 17.08%<br>4 40-49: 14.82%<br>5 50-59: 13.53%<br>6 60-69: 8.26%<br>7 70-79: 3.99%<br>8 >80: 2.16%	1.99%
Gender	Patient's gender	Categorical	Female: 49.39%<br>Male: 50.05%	0.56%
Region	Health unit in Canada	Categorical	40	0.00%
Exposure	The type of being exposed to someone with COVID-19	Categorical	Close contact: 31.21%<br>Outbreak: 10.78%<br>Travel-related: 1.14%	56.87%
Province	Province in Canada where case is reported	Categorical	Ontario: 70.25%<br>Alberta: 29.75%	0.00%

Note: SD: standard deviation

Table E.5: Descriptive statistics for the COVID-19 dataset.

E.6 FDA Adverse Event Reporting System

The next dataset contains the reports submitted to the FDA Adverse Event Reporting System for patients with adverse events. The binary outcome of interest for this dataset is whether or a patient has died. Our primary goal with this dataset is to explore the relationship between patient mortality and various predictors, including event date, gender, age, weight, drug name and the indication for drug use. Detailed statistics for these variables can be found in **Table E.6**.

Variable	Description	Type	Mean (SD) or level count (% of total size) or number of categories	Missingness (% of the total size)
Outcome	Whether a patient has died	Categorical	1 Death: 9.94%<br>0 Non-death: 90.06%	0.00%
Event date	Date the adverse event occurred	Numeric (difference from 1/1/2020)	466.52 (827.94)	62.26%
Gender	Patient's gender	Categorical	Female: 51.82%<br>Male: 37.85%	10.33%
Age	Patient's age in years	Numeric	55.90 (20.80)	33.41%
Weight	Patient's weight in kg	Numeric	73.05 (25.70)	74.13%
Drug name	Name of medicinal product	Categorical	10,545	0.00%
Indication	Medical terminology describing the indication for use	Categorical	4,287	0.00%

Note: SD: standard deviation

Table E.6: Descriptive statistics for the FAERS dataset.

E.7 Florida Hospital Discharges

This dataset contains over 2.3 million inpatient discharge records in 2007 from community hospitals in Florida from State Inpatient Databases that are used to track the trends in healthcare utilization, access, charges, quality and outcomes in United States. We are interested in exploring the relationship between length of stay and its demographic and health factors. Specifically, the covariates of interest include age, female, race, admission type, aweekend, DRG, DX1, primary payer, total charges and zip code. The outcome is created by classifying the patients into two groups based on the median length of their stay. A value of 1 is assigned if the patient's length of stay is greater than or equal to 3 days and 0 otherwise. Detailed statistics of the variables are displayed in **Table E.7**.

Variable	Description	Type	Mean (SD) or level count (% of total size) or number of categories	Missingness (% of the total size)
Outcome	Whether a patient's length of stay is greater than 3 days	Categorical	1: 60.46%<br>0: 39.54%	0.00%
AGE	Patient's age in years	Numeric	51.23 (27.04)	0.00%
FEMALE	Whether a patient's gender is female	Categorical	1 Female: 56.02%<br>0 Male: 43.98%	0.00%
RACE	Patient's race	Categorical	1 White: 65.53%<br>2 Black: 16.92%<br>3 Hispanic: 13.28%<br>4 Asian or Pacific Islander: 0.76%<br>5 Native American: 0.27%<br>6 Other: 2.47%	0.76%
ATYPE	Admission type	Categorical	1 Emergency: 54.15%<br>2 Urgent: 16.43%<br>3 Elective: 20.73%<br>4 Newborn: 8.17%<br>5 Trauma Center: 0.52%	0.00%
AWEEKEND	Whether a patient's admission day is on a weekend	Categorical	1 Admitted Saturday - Sunday: 19.51%<br>0 Admitted Monday - Friday: 80.49%	0.00%
DRG	Diagnosis Related Group	Categorical	861	0.00%
DX1	ICD-9-CM Diagnosis	Categorical	7,380	0.00%
PAY1	Expected primary payer (Medicare, Medicaid, private insurances, etc.)	Categorical	1 Medicare: 42.71%<br>2 Medicaid: 17.50%<br>3 Private insurance: 27.52%<br>4 Self-pay: 6.23%<br>5 No charge: 2.28%<br>6 Other: 3.77%	0.00%
TOTCHG	Total charges	Numeric	33,604.48 (52,812.95)	0.01%
CHG1	All inclusive detailed charges	Numeric	1,818.47 (2,841.07)	0.00%

ZIP	Zip code	Categorical	14,728	0.64%

Note: SD: standard deviation

Table E.7: Descriptive statistics for the hospital Florida dataset.

E.8 Medical Information Mart for Intensive Care III

The dataset is extracted from the MIMIC-III relational database (version 1.4), which contains deidentified clinical data of the patients who were admitted to the Beth Israel Deaconess Medical Center in Boston, Massachusetts [12–14]. It contains various tables of patient data regarding demographics, admission information, lab tests, diagnosis codes, caregiver information, and discharge notes. We use this dataset to investigate the relationship between 30-day readmission and its related demographics, vital signs and lab test values. The demographics include the age of the patients when they were first admitted to the ICU, their ethnicity group and admission type. The vital signs consider the (systolic and diastolic) blood pressure, heart rate and respiration rate. Several lab measurements are also incorporated into the analysis. The selection criteria for readmitted patients are to include those who were readmitted within 30-day of initial hospital discharge from the ICU. The patients who were readmitted to the ICU are assigned a label of 1, while those who were not readmitted are assigned a label of 0. **Table E.8** summarizes the descriptive statistics of the selected variables.

Variable	Description	Type	Mean (SD) or level count (% of total size) or number of categories	Missingness (% of the total size)
Readmission	Whether a patient is re-admitted to ICU	Categorical	1 Yes: 21.01%<br>0 No: 78.99%	0.00%
Age	Patient's age in the time of first admission	Numeric	63.43 (16.16)	0.00%
Ethnicity	Patient's ethnicity group	Categorical	39	15.14%
Admission type	Patient's admission type	Categorical	Elective: 18.10%<br>Emergency: 78.52%<br>Urgent: 3.37%	0.00%
Heart rate	Vital sign for heart rate	Numeric	87.86 (15.89)	0.47%
NT-proBNP	Lab test for N-terminal prohormone of brain natriuretic peptide	Numeric	4.10 (1.17)	43.55%
Creatinine	Lab test for serum creatinine	Numeric	4.10 (1.17)	43.54%
Bun	Lab test for blood urea nitrogen	Numeric	4.10 (1.17)	43.53%
Potassium	Lab test for potassium	Numeric	4.10 (1.17)	43.52%
Cholesterol	Lab test for cholesterol	Numeric	4.10 (1.17)	43.54%

Note: SD: standard deviation

Table E.8: Descriptive statistics for the MIMIC-III dataset.

E.9 New York Hospital Discharges

This dataset consists of over 2.6 million inpatient discharge records in 2007 from community hospitals in New York from State Inpatient Databases that are used to track the trends in healthcare utilization, access, charges, quality and outcomes in the United States. We are interested in examining the relationship between length of stay and demographic and health factors. Specifically, the covariates of interest include age, female, race, admission type, aweekend, DRG, DX1, primary payer, total charges, zip code, chronic conditional indicators and procedure classes for ICD-10-PCS procedure codes. The outcome is created by classifying the patients into two groups based on the median of their length of stay. A value of 1 is assigned if the patient's length of stay is greater than or equal to 3 days and 0 otherwise. Detailed statistics of the variables are displayed in **Table E.9**.

Variable	Description	Type	Mean (SD) or level count (% of total size) or number of categories	Missingness (% of the total size)
Outcome	Whether a patient's length of stay is greater than 3 days	Categorical	1: 61.82%<br>0: 38.18%	0.00%
AGE	Patient's age in years	Numeric	48.87 (27.36)	0.00%
FEMALE	Whether a patient's gender is female	Categorical	1 Female: 56.68%<br>0 Male: 43.32%	0.00%
RACE	Patient's race	Categorical	1 White: 56.73%<br>2 Black: 17.43%<br>3 Hispanic: 13.56%<br>4 Asian or Pacific Islander: 3.38%<br>5 Native American: 1.01%<br>6 Other: 5.90%	1.98%
ATYPE	Admission type	Categorical	1 Emergency: 60.22%<br>2 Urgent: 9.86%<br>3 Elective: 20.86%<br>4 Newborn: 8.90%<br>5 Trauma Center: 0.00%	0.16%
AWEEKEND	Whether a patient's admission day is on a weekend	Categorical	1 Admitted Saturday - Sunday: 19.26%<br>0 Admitted Monday - Friday: 80.74%	0.00%
DRG	Diagnosis Related Group	Categorical	863	0.00%
DX1	ICD-9-CM Diagnosis	Categorical	7,956	0.00%
PAY1	Expected primary payer (Medicare, Medicaid, private insurances, etc.)	Categorical	1 Medicare: 36.08%<br>2 Medicaid: 23.69%<br>3 Private insurance: 32.38%<br>4 Self-pay: 5.44%<br>5 No charge: 0.17%<br>6 Other: 2.23%	0.00%
TOTCHG	Total charges	Numeric	24,628.84 (43,545.43)	0.01%
ZIP	Zip code	Categorical	10,814	0.00%

CHRON1	ICD-9-CM Chronic Condition Indicators	Categorical	1 Chronic condition: 40.86%<br>0 Non-chronic condition: 59.14%	0.00%
CHRONB1	Chronic Condition Indicators - body system	Categorical	19	0.00%
PCLASS1	Procedure Classes Refined for ICD-10-PCS procedure codes	Categorical	1 Minor diagnostic: 15.19%<br>2 Minor therapeutic: 31.30%<br>3 Major diagnostic: 0.54%<br>4 Major Therapeutic: 25.86%	27.12%

Note: SD: standard deviation

Table E.9: Descriptive statistics for the hospital New York dataset.

E.10 Nexoid COVID-19 Survival Calculator

The COVID-19 survival dataset that is used in the study is a web-based survey data collected by the research team by Nexoid, a company in the United Kingdom. They collect demographic, socioeconomic and health-related information of individuals to predict two crucial aspects related to COVID-19: the probability of being infected with COVID-19 as well as the probability of mortality associated with COVID-19. In our study, we focus on the probability of COVID-19 infection using important demographic, behavioral and health factors including age, sex, race, smoking, nursing home, COVID-19 symptoms, COVID-19 contact, health worker, and the presence of comorbidities such as asthma, kidney disease, liver disease, heart disease, lung disease, diabetes, and hypertension. The outcome of interest is determined by the risk scores of getting infected with COVID-19. The patients whose risk scores exceed the mean risk score are considered as having a high risk of contracting COVID-19, while those with scores below the mean are classified as having a low risk. **Table E.10** summarizes the basic statistics of the selected variables.

Variable	Description	Type	Mean (SD) or level count (% of total size)	Missingness (% of the total size)
Outcome	Whether a patient has a high risk of getting infected with COVID-19	Categorical	1 Yes: 39.20%<br>0 No: 60.80%	0.00%
Age	Age group in years	Numeric	1 0_10: 0.58%<br>2 10_20: 3.35%<br>3 20_30: 21.56%<br>4 30_40: 29.84%<br>5 40_50: 21.09%<br>6 50_60: 12.48%<br>7 60_70: 7.47%<br>8 70_80: 2.97%<br>9 80_90: 0.54%<br>10 90_100: 0.12%<br>11 100_110: 0.01%	0.00%
Sex	Patient's gender	Categorical	Female: 63.13%<br>Male: 36.53%	0.34%
Race	Patient's race	Categorical	White: 24.19%<br>Hispanic: 1.40%<br>Asian: 1.21%<br>Mixed: 0.96%<br>Black: 0.46%<br>Other: 0.32%	71.45%
Smoking	Type of smoking	Categorical	Heavy: 1.68%<br>Medium: 7.64%<br>Light: 4.44%<br>Quit0: 5.54%<br>Quit5: 6.58%<br>Quit10: 9.23%<br>Vape: 5.95%<br>Never smoked: 58.74%	0.19%
BMI	Body mass index	Numeric	29.37 (7.81)	0.00%
House count	House person count	Numeric	3.14 (1.57)	0.00%
Public transport count	Number of public transports used	Numeric	0.38 (1.70)	71.12%
Nursing home	Whether it is a nursing home	Categorical	1: 0.07%<br>0: 99.93%	0.00%
COVID-19 symptoms	Whether a patient shows symptoms of COVID-19	Categorical	1: 2.04%<br>0: 97.96%	0.00%
COVID-19 contact	Whether a patient has close contact with someone infected with	Categorical	1: 4.33%<br>0: 95.67%	0.00%

	COVID-19			
Health worker	Whether a patient is a healthcare worker	Categorical	1: 1.79%<br>0: 98.21%	0.00%
Asthma	Whether a patient has asthma	Categorical	1: 15.26%<br>0: 84.74%	0.00%
Kidney disease	Whether a patient has kidney disease	Categorical	1: 0.36%<br>0: 99.64%	0.00%
Liver disease	Whether a patient has liver disease	Categorical	1: 0.21%<br>0: 99.79%	0.00%
Heart disease	Whether a patient has heart disease	Categorical	1: 1.87%<br>0: 98.13%	0.00%
Lung disease	Whether a patient has lung disease	Categorical	1: 1.45%<br>0: 98.55%	0.00%
Diabetes	Whether a patient has diabetes	Categorical	1: 6.17%<br>0: 93.83%	0.00%
Hypertension	Whether a patient has hypertension	Categorical	1: 13.83%<br>0: 86.17%	0.00%

Note: SD: standard deviation

Table E.10: Descriptive statistics for the Nexoid dataset.

E.11 Texas Inpatients

Texas inpatient dataset contains 75 variables. Similar to the Washington state hospital discharge data, in this dataset, we explore the relationship between those demographic and health factors and the length of stay in the Texas hospitals. The involved covariates include age, sex, race, ethnicity, location, weekday, risk mortality, severity, DRG and fees with detailed descriptions in **Table E.11**. According to their length of stay in the hospital, the patients are classified into two groups, and the outcome is assigned a value of 1 if the patient's length of stay is greater than or equal to 3 days and 0 otherwise.

Variable	Description	Type	Mean (SD) or level count (% of total size) or number of categories	Missingness (% of the total size)
Outcome	Whether a patient's length of stay is greater than 3 days	Categorical	1: 59.58%<br>0: 40.42%	0.00%
Age	Patient's age groups	Numeric	0: 11.95%<br>1: 1.66%<br>2: 1.62%<br>3: 1.08%<br>4: 1.39%<br>5: 1.58%<br>6: 1.58%<br>7: 4.84%<br>8: 5.28%<br>9: 4.94%<br>10: 3.75%<br>11: 3.41%<br>12: 3.93%<br>13: 4.96%<br>14: 5.58%<br>15: 5.95%<br>16: 6.47%<br>17: 6.01%<br>18: 5.78%<br>19: 5.32%<br>20: 3.94%<br>21: 2.40%<br>22: 0.21%<br>23: 2.68%<br>24: 2.86%<br>25: 0.54%<br>26: 0.28%	0.00%

Sex	Patient's gender	Categorical	Female: 56.25%<br>Male: 37.16%	6.58%
Race	Patient's race	Categorical	1 American Indian/Eskimo/Aleut: 0.77%<br>2 Asian or Pacific Islander: 1.68%<br>3 Black: 12.61%<br>4 White: 61.45%<br>5 Other: 23.35%	0.13%
Ethnicity	Whether a patient is of Hispanic origin	Categorical	1 Hispanic Origin: 28.14%<br>2 Not of Hispanic Origin: 70.45%	1.41%
Location	Patient's mailing address in Texas and contiguous states	Categorical	AR: 0.48%<br>FC: 0.25%<br>LA: 0.21%<br>NM: 0.57%<br>OK: 0.32%<br>TX: 97.21%<br>XX: 0.02%<br>ZZ: 0.92%	0.02%
Weekday	The day of week a patient is admitted	Categorical	1 Monday: 16.97%<br>2 Tuesday: 17.22%<br>3 Wednesday: 16.38%<br>4 Thursday: 15.89%<br>5 Friday: 14.98%<br>6 Saturday: 9.42%<br>7 Sunday: 9.14%	0.00%
Risk mortality	Risk of mortality score from the All Patient Refined (APR) Diagnosis Related Group (DRG) from the 3M™ APR-DRG Grouper.	Categorical	0 No class specified: 0.10%<br>1 Minor: 60.15%<br>2 Moderate: 20.26%<br>3 Major: 13.26%	0.00%

			4 Extreme: 6.22%	
Severity	Severity of illness score from the All Patient Refined (APR) Diagnosis Related Group (DRG) from the 3M™ APR-DRG Grouper.	Categorical	0 No class specified: 0.10%<br>1 Minor: 35.40%<br>2 Moderate: 33.39%<br>3 Major: 22.76%<br>4 Extreme: 8.35%	0.00%
DRG	All Patient Refined (APR) Diagnosis Related Group (DRG) as assigned by 3M APR-DRG Grouper	Categorical	316	0.00%
Fees	Total non-covered amount of the charge	Numeric	57.51 (1375.47)	0.02%

Note: SD: standard deviation

Table E.11: Descriptive statistics for the Texas inpatient dataset.

E.12 Washington State Hospital Discharges

The seventh dataset, Washington State Hospital Discharge dataset, contains over 350 variables. Among these, we model the relationship between those demographic and health factors and the length of stay in the hospital. The covariates were: age, atype, aweekend, died, DRG, primary diagnosis code, and ZIP code. A detailed description of these variables is displayed in **Table E.12**. The outcome of our study categorizes patients into two groups based on their length of stay. A value of 1 is assigned if the patient's length of stay is greater than or equal to 3 days and 0 otherwise.

Variable	Description	Type	Mean (SD) or 1 count (% of total size) or number of categories	Missingness (% of the total size)
Outcome	Whether a patient's length of stay is greater than 3 days	Categorical	1: 49.07%<br>0: 50.93%	0.00%
Age	Patient's age in years	Numeric	45.58 (28.45)	0.01%
Atype	Admission type	Categorical	1: 34.69%<br>2: 18.03%<br>3: 34.23%<br>4: 12.81%<br>5: 0.23%	0.00%
Aweekend	Whether admission occurs on a weekend	Categorical	1: 19.32%<br>0: 80.68%	0.00%
Died	Whether a patient died during hospitalization	Categorical	1: 1.99%<br>0: 98.01%	0.00%
DRG	Diagnosis-related-group (DRG) in effect on discharge date	Categorical	862	0.00%
DX1	Primary diagnosis	Categorical	5,864	15.14%
ZIP	Patient's ZIP code	Categorical	4,272	0.06%

Note: SD: standard deviation

Table E.12: Descriptive statistics for the hospital Washington dataset.

E.13 Washington State Hospital Discharges (2008)

This dataset contains 652,340 inpatient discharge records in 2008 from community hospitals in Washington from State Inpatient Databases that are used to track the trends in healthcare utilization, access, charges, quality and outcomes in the United States. We are interested in examining the relationship between length of stay and demographic and health factors. Specifically, the covariates of interest include age, female, race, admission type, aweekend, DRG, DX1, primary payer, total charges, zip code, chronic conditional indicators, and procedure classes for ICD-10-PCS procedure codes, comorbidity measures for alcohol abuse, depression, hypertension and obesity. The outcome is created by classifying the patients into two groups based on the median of their length of stay. A value of 1 is assigned if the patient's length of stay is greater than or equal to 2 days and 0 otherwise. Detailed statistics of the variables are displayed in **Table E.13**.

Variable	Description	Type	Mean (SD) or level count (% of total size) or number of categories	Missingness (% of the total size)
Outcome	Whether a patient's length of stay is greater than 2 days	Categorical	1: 74.58%<br>0: 25.42%	0.00%
AGE	Patient's age in years	Numeric	45.79 (28.43)	0.01%
FEMALE	Whether a patient's gender is female	Categorical	1 Female: 58.69%<br>0 Male: 41.31%	0.01%
RACE	Patient's race	Categorical	1 White: 23.86%<br>2 Black: 1.18%<br>3 Hispanic: 2.80%<br>4 Asian or Pacific Islander: 1.31%<br>5 Native American: 0.48%<br>6 Other: 0.02%	70.34%
ATYPE	Admission type	Categorical	1 Emergency: 35.59%<br>2 Urgent: 17.80%<br>3 Elective: 33.22%<br>4 Newborn: 12.74%<br>5 Trauma Center: 0.65%	0.00%
AWEEKEND	Whether a patient's admission day is on a weekend	Categorical	1 Admitted Saturday - Sunday: 19.50%<br>0 Admitted Monday - Friday: 80.50%	0.00%
DRG	Diagnosis Related Group	Categorical	746	0.00%
DX1	ICD-9-CM Diagnosis	Categorical	6,149	0.01%
PAY1	Expected primary payer (Medicare, Medicaid, private insurances, etc.)	Categorical	1 Medicare: 31.21%<br>2 Medicaid: 20.03%<br>3 Private insurance: 42.89%<br>4 Self-pay: 2.80%<br>5 No charge: 0.60%<br>6 Other: 2.46%	0.00%
TOTCHG	Total charges	Numeric	26,040.52 (43,943.20)	0.01%
ZIP	Zip code	Categorical	4,191	0.06%
CHRON1	ICD-9-CM Chronic	Categorical	1 Chronic condition: 35.16%	0.01%

	Condition Indicators		0 Non-chronic condition: 64.82%	
CHRONB1	Chronic Condition Indicators - body system	Categorical	19	0.01%
PCLASS1	Procedure Classes Refined for ICD-10-PCS procedure codes	Categorical	1 Minor diagnostic: 6.66%<br>2 Minor therapeutic: 24.98%<br>3 Major diagnostic: 0.44%<br>4 Major Therapeutic: 31.07%	36.84%
CM_ALCOH	AHRQ comorbidity measure for ICD-9-CM codes: alcohol abuse	Categorical	1 Comorbidity is present: 3.01%<br>0 Comorbidity is not present: 96.99%	0.00%
CM_DEPRE	AHRQ comorbidity measure for ICD-9-CM codes: depression	Categorical	1 Comorbidity is present: 6.31%<br>0 Comorbidity is not present: 93.69%	0.00%
CM_HTN_C	AHRQ comorbidity measure for ICD-9-CM codes: hypertension (combine uncomplicated and complicated)	Categorical	1 Comorbidity is present: 28.69%<br>0 Comorbidity is not present: 71.31%	0.00%
CM_OBESE	AHRQ comorbidity measure for ICD-9-CM codes: obesity	Categorical	1 Comorbidity is present: 5.55%<br>0 Comorbidity is not present: 94.45%	0.00%

Note: SD: standard deviation

Table E.13: Descriptive statistics for the hospital Washington2008 dataset.

E.14 Breast Cancer

The Breast Cancer dataset is a dataset related to the recurrence of breast cancer patients, provided by the University Medical Centre, Institute of Oncology in Yugoslavia [1]. The dataset consists of 277 health records. The target variable is whether a patient experienced breast cancer recurrence and the relevant factors cover the patient's age, menopausal status, tumor size, node caps, breast, breast quad, irradiation, lymph nodes, and degree of malignancy. The full description of the variables is in **Table E.14.**

Variable	Description	Type	Mean (SD) or level count (% of total size)	Missingness (% of the total size)
Outcome	Whether a patient had a recurrence event	Categorical	1: 29.24%<br>0: 70.76%	0.00%
Age	Patient's age	Numeric	3.64 (1.01)	0.00%
Menopausal status	Patient's menopausal status	Categorical	Ge40: 44.40%<br>Lt40: 1.81%<br>Premeno: 53.79%	0.00%
Tumor size	Size of a tumor	Numeric	1: 2.89%<br>2: 10.11%<br>3: 10.47%<br>4: 17.33%<br>5: 18.41%<br>6: 20.58%<br>7: 6.86%<br>8: 7.94%<br>9: 1.08%<br>10: 1.44%<br>11: 2.89%	0.00%
Node caps	Capsule of a lymph node	Categorical	Yes: 20.22%<br>No: 79.78%	0.00%
Breast	Location of the breast	Categorical	Left: 52.35%<br>Right: 47.65%	0.00%
Breast quad	Division of the breast into four sections or quadrants	Categorical	Central: 7.58%<br>Left low: 38.27%<br>Left up: 33.94%<br>Right low: 8.30%<br>Right up: 11.91%	0.00%
Irradiation	Whether a patient undergone radiation therapy	Categorical	Yes: 22.38%<br>No: 77.62%	0.00%
Lymph nodes	Involved lymph nodes	Numeric	1: 75.45%<br>2: 20.94%<br>3: 1.08%<br>4: 2.17%<br>5: 0.36%	0.00%
Degree of malignancy	Severity of a malignant tumor	Numeric	1: 23.83%<br>2: 46.57%<br>3: 29.60%	0.00%

Note: SD: standard deviation

Table E.14: Descriptive statistics for the Breast Cancer dataset.

E.15 Breast Cancer Coimbra

The Breast Cancer Coimbra dataset comprises the health information of women with breast cancer measured by the Gynaecology Department of the University Hospital Centre of Coimbra in the years

from 2009 to 2013 [3]. It has 116 observations with 10 variables. The outcome describes whether the female patient is with breast cancer. The remaining 9 variables include patient's age, body mass index, glucose, insulin, homeostasis model assessment, leptin, adiponectin, resistin and MCP-1. The detailed characteristics of the variables are described in **Table E.15**.

Variable	Description	Type	Mean (SD) or level count (% of total size)	Missingness (% of the total size)
Outcome	Whether a patient had breast cancer	Categorical	1: 44.83%<br>0: 55.17%	0.00%
Age	Patient's age	Numeric	57.30 (16.11)	0.00%
Body mass index	Patient's body mass index	Numeric	27.58 (5.02)	0.00%
Glucose	Patient's serum glucose	Numeric	97.79 (22.53)	0.00%
Insulin	Patient's serum insulin	Numeric	10.01 (10.07)	0.00%
Homeostasis model assessment	An index to evaluate insulin resistance	Numeric	2.69 (3.64)	0.00%
Leptin	Serum values of Leptin	Numeric	26.62 (19.18)	0.00%
Adiponectin	Serum values of Adiponectin	Numeric	10.18 (6.84)	0.00%
Resistin	Serum values of Resistin	Numeric	14.73 (12.39)	0.00%
MCP-1	Serum values of Chemokine Monocyte Chemoattractant Protein 1	Numeric	534.65 (345.91)	0.00%

Note: SD: standard deviation

Table E.15: Descriptive statistics for the Breast Cancer Coimbra dataset.

E.16 Colposcopy/Schiller

The Colposcopy/Schiller dataset is one of the three modality colposcopy data that evaluates the subjective quality assessment of digital colposcopies collected by Hospital Universitario de Caracas [4]. The data have 287 health records. The outcome is whether the subjective assessment is good or not. The remaining 62 variables are associated with color information, image area and coverage in digital cervical imaging. **Table E.16** summarizes the description of the dataset variables.

Variable	Description	Type	Mean (SD) or level count (% of total size)	Missingness (% of the total size)
Outcome	Whether the subjective assessment is good	Categorical	1: 72.83%<br>0: 27.17%	0.00%
Cervix area	Image area with cervix	Numeric	0.49 (0.24)	0.00%
Os area	Image area with external os	Numeric	0.01 (0.01)	0.00%
Walls area	Image area with vaginal walls	Numeric	0.18 (0.19)	0.00%
Speculum area	Image area with the speculum	Numeric	0.26 (0.19)	0.00%
Artifacts area	Image area with artifacts	Numeric	0.04 (0.04)	0.00%
Cervix artifacts area	Cervix area with the artifacts	Numeric	0.03 (0.03)	0.00%
Os artifacts area	External os area with the artifacts	Numeric	0.04 (0.13)	0.00%
Walls artifacts area	Vaginal walls with the artifacts	Numeric	0.03 (0.08)	0.00%
Speculum artifacts area	Speculum area with the artifacts	Numeric	0.01 (0.06)	0.00%
Cervix specularities area	Cervix area with the specular reflections	Numeric	0.01 (0.02)	0.00%
Os specularities area	External os area with the specular reflections	Numeric	0.01 (0.02)	0.00%
Walls specularities area	Vaginal walls area with the specular reflections	Numeric	0.02 (0.05)	0.00%
Speculum specularities area	Speculum area with the specular reflections	Numeric	0.14 (0.15)	0.00%
Specularities area	Total area with specular reflections	Numeric	0.05 (0.05)	0.00%
Area h max diff	Maximum area differences between the four cervix quadrants	Numeric	0.21 (0.16)	0.00%
Rgb cervix r mean	Average color information in the cervix (R channel)	Numeric	48.39 (26.78)	0.00%
Rgb cervix r std	Stddev color information in the cervix (R channel)	Numeric	33.28 (14.89)	0.00%
Rgb cervix r mean minus std	(Avg - stddev) color information in the cervix (R channel)	Numeric	15.11 (24.53)	0.00%
Rgb cervix r mean plus std	(Avg + stddev) information in the cervix (R channel)	Numeric	81.67 (35.72)	0.00%
Rgb cervix g mean	Average color information in the cervix (G channel)	Numeric	41.70 (23.03)	0.00%
Rgb cervix g std	Stddev color information in the cervix (G channel)	Numeric	30.92 (15.34)	0.00%
Rgb cervix g mean minus std	(Avg - stddev) color information in the cervix (G	Numeric	10.78 (19.12)	0.00%

	channel)			
Rgb cervix g mean plus std	(Avg + stddev) information in the cervix (G channel)	Numeric	72.62 (34.15)	0.00%
Rgb cervix b mean	Average color information in the cervix (B channel)	Numeric	84.10 (43.83)	0.00%
Rgb cervix b std	Stddev color information in the cervix (B channel)	Numeric	47.43 (20.61)	0.00%
Rgb cervix b mean minus std	(Avg - stddev) color information in the cervix (B channel)	Numeric	36.67 (31.48)	0.00%
Rgb cervix b mean plus std	(Avg + stddev) information in the cervix (B channel)	Numeric	131.54 (60.83)	0.00%
Rgb total r mean	Average color information in the image (R channel)	Numeric	75.09 (27.43)	0.00%
Rgb total r std	Stddev color information in the image (R channel)	Numeric	55.43 (19.24)	0.00%
Rgb total r mean minus std	(Avg - stddev) color information in the image (R channel)	Numeric	19.66 (22.76)	0.00%
Rgb total r mean plus std	(Avg + stddev) color information in the image (R channel)	Numeric	130.52 (41.56)	0.00%
Rgb total g mean	Average color information in the image (G channel)	Numeric	65.66 (26.87)	0.00%
Rgb total g std	Stddev color information in the image (G channel)	Numeric	50.91 (20.65)	0.00%
Rgb total g mean minus std	(Avg - stddev) color information in the image (G channel)	Numeric	14.75 (17.84)	0.00%
Rgb total g mean plus std	(Avg + stddev) color information in the image (G channel)	Numeric	116.57 (44.48)	0.00%
Rgb total b mean	Average color information in the image (B channel)	Numeric	102.45 (31.72)	0.00%
Rgb total b std	Stddev color information in the image (B channel)	Numeric	64.26 (16.25)	0.00%
Rgb total b mean minus std	(Avg - stddev) color information in the image (B channel)	Numeric	38.18 (26.79)	0.00%
Rgb total b mean plus std	(Avg + stddev) color information in the image (B channel)	Numeric	166.71 (42.70)	0.00%
Hsv cervix h mean	Average color information in the cervix (H channel)	Numeric	3.92 (0.38)	0.00%
Hsv cervix h std	Stddev color information in the cervix (H channel)	Numeric	2.44 (0.56)	0.00%
Hsv cervix s mean	Average color information in	Numeric	127.81 (51.19)	0.00%

	the cervix (S channel)			
Hsv cervix s std	Stddev color information in the cervix (S channel)	Numeric	48.70 (23.93)	0.00%
Hsv cervix v mean	Average color information in the cervix (V channel)	Numeric	86.19 (43.67)	0.00%
Hsv cervix v std	Stddev color information in the cervix (V channel)	Numeric	47.20 (20.44)	0.00%
Hsv total h mean	Average color information in the image (H channel)	Numeric	4.00 (0.33)	0.00%
Hsv total h std	Stddev color information in the image (H channel)	Numeric	2.51 (0.19)	0.00%
V total s mean	Average color information in the image (S channel)	Numeric	111.00 (42.07)	0.00%
Hsv total s std	Stddev color information in the image (S channel)	Numeric	53.33 (20.95)	0.00%
Hsv total v mean	Average color information in the image (V channel)	Numeric	106.63 (32.58)	0.00%
Hsv total v std	Stddev color information in the image (V channel)	Numeric	64.24 (16.39)	0.00%
Fit cervix hull rate	Coverage of the cervix convex hull by the cervix	Numeric	0.90 (0.20)	0.00%
Fit cervix hull total	Image coverage of the cervix convex hull	Numeric	0.52 (0.24)	0.00%
Fit cervix bbox rate	Coverage of the cervix bounding box by the cervix	Numeric	0.77 (0.19)	0.00%
Fit cervix bbox total	Image coverage of the cervix bounding box	Numeric	0.60 (0.25)	0.00%
Fit circle rate	Coverage of the cervix circle by the cervix	Numeric	0.57 (0.14)	0.00%
Fit circle total	Image coverage of the cervix circle	Numeric	0.84 (0.44)	0.00%
Fit ellipse rate	Coverage of the cervix ellipse by the cervix	Numeric	0.93 (0.21)	0.00%
Fit ellipse total	Image coverage of the cervix ellipse	Numeric	0.50 (0.23)	0.00%
Fit ellipse goodness	Goodness of the ellipse fitting	Numeric	135.51 (87.55)	0.00%
Distance to center cervix	Distance between the cervix center and the image center	Numeric	0.50 (0.26)	0.00%
Distance to center os	Distance between the cervical os center and the image center	Numeric	0.46 (0.17)	0.00%

Note: SD: standard deviation

Table E.16: Descriptive statistics for the Colposcopy/Schiller dataset.

E.17 Danish Colorectal Cancer Group

The Danish Colorectal Cancer Group (DCCG) database registered all patients in Denmark who were diagnosed with colorectal cancer or treated in a public Danish hospital since 2001 [5]. The original data was obtained from the database with a total of 12,855 observations. After data preprocessing, we draw a random sample of 700 observations for analysis. The outcome of interest is whether a patient had a postoperative medical complication, and associated risk factors include age, gender, ASA score (i.e., pre-operative fitness score), localization of the tumor, procedure, pathological tumor (T) stage and node (N) stage, number of removed lymph nodes and number of lymph nodes with metastasis and unplanned intraoperative adverse event. The statistics descriptives for the chosen DCCG variables are displayed in **Table E.17**.

Variable	Description	Type	Mean (SD) or level count (% of total size)	Missingness (% of the total size)
Postoperative medical complication	Whether a patient had an unwanted medical condition (outcome)	Categorical	1: 16.26%<br>0: 83.74%	0.00%
Age	Patient's age	Numeric	72.44 (9.91)	0.00%
Gender	Patient's gender	Categorical	Female: 55.40%<br>Male: 44.60%	0.00%
ASA score	Score to evaluate the overall health status according to the American Society of Anesthesiologists Physical Status Classification System	Categorical	ASA1: 17.44%<br>ASA2: 54.79%<br>ASA3: 24.96%<br>ASA4: 1.26%	1.55%
Localization of the tumor	A place in the body where the tumor was located	Categorical	Ascending colon: 34.41%<br>Caecum: 40.17%<br>Hepatic flexure: 14.15%<br>Splenic flexure: 0.08%<br>Transverse colon: 11.19%	0.00%
Procedure	Surgical procedure that was performed	Categorical	Right hemicolectomy: 85.52%<br>Extended right hemicolectomy: 14.48%	0.00%
Pathological T stage	Measure to classify the extent of cancer (T stage) by the TNM staging system	Categorical	pT1 stage: 5.60%<br>pT2 stage: 13.12%<br>pT3 stage: 60.32%<br>pT4 stage: 18.39%<br>pTx or pT0 stage: 0.58%	1.99%
Pathological N stage	Measure to classify the extent of cancer spread to regional lymph nodes (N stage) by the TNM staging system	Categorical	pN1 stage: 21.62%<br>pN2 stage: 14.83%<br>pNx or pN0 stage: 63.16%	0.39%
Number of removed lymph nodes	Pathologically shown total number of removed lymph nodes	Numeric	39.05 (108.69)	0.00%
Number of lymph nodes with metastasis	Pathologically shown total number of lymph nodes with metastasis	Numeric	1.71 (4.14)	1.26%
Unplanned intraoperative adverse event	Whether there was an unexpected intraoperative adverse event	Categorical	Yes: 1.91%<br>No: 91.53%	6.56%

Note: SD: standard deviation

Table E.17: Descriptive statistics for the DCCG dataset.

E.18 Diabetic Retinopathy

The Diabetic Retinopathy dataset comprises the detections of diabetic retinopathy represented by either detected lesions or image-level descriptors from Messidor image set [6]. The data size is 1,151. The outcome in the selected sample is whether a patient's image contains signs of diabetic retinopathy, and the other imaging information such as the numbers of the detection of microaneurysms found across various confidence levels, are considered as the relevant factors. and we randomly draw a sample with a size of 600. The outcome in the selected sample is whether **Table E.18** summarizes the statistics descriptives of the dataset.

Variable	Description	Type	Mean (SD) or level count (% of total size)	Missingness (% of the total size)
Outcome	Whether a patient's image contains signs of diabetic retinopathy.	Categorical	1: 54.67%<br>0: 45.33%	0.00%
MA1	Number of the detection of microaneurysms found at various confidence levels	Numeric	39.67 (26.81)	0.00%
MA2	Number of the detection of microaneurysms found at various confidence levels	Numeric	38.03 (25.03)	0.00%
MA3	Number of the detection of microaneurysms found at various confidence levels	Numeric	36.17 (23.53)	0.00%
MA4	Number of the detection of microaneurysms found at various confidence levels	Numeric	33.22 (21.64)	0.00%
MA5	Number of the detection of microaneurysms found at various confidence levels	Numeric	29.58 (19.86)	0.00%
MA6	Number of the detection of microaneurysms found at various confidence levels	Numeric	21.75 (15.27)	0.00%
MA7	Number of the detection of microaneurysms found at various confidence levels	Numeric	65.14 (59.26)	0.00%
Exu1	Number of exudate pixels at various confidence levels	Numeric	23.45 (22.00)	0.00%
Exu2	Number of exudate pixels at various confidence levels	Numeric	8.92 (11.90)	0.00%
Exu3	Number of exudate pixels at various confidence levels	Numeric	2.03 (4.51)	0.00%
Exu4	Number of exudate pixels at various confidence levels	Numeric	0.70 (3.07)	0.00%
Exu5	Number of exudate pixels at various confidence levels	Numeric	0.27 (1.31)	0.00%
Exu6	Number of exudate pixels at various confidence levels	Numeric	0.11 (0.49)	0.00%
Exu7	Number of exudate pixels at various confidence levels	Numeric	0.05 (0.22)	0.00%
Euclidean distance	The Euclidean distance of the center of the macula and the center of the optic disk	Numeric	0.52 (0.03)	0.00%
Diameter of optic disk	The diameter of the optic disk	Numeric	0.11 (0.02)	0.00%
Quality	Quality assessment	Categorical	1: 99.67%	0.00%

			0: 0.33%	
Pre-screening	Pre-screening for whether a patient had a severe retinal abnormality	Categorical	1: 92.00%<br>0: 8.00%	0.00%
AM_FM	Confidence of the detection of Diabetic Retinopathy indicated by the Amplitude-Modulation Frequency-Modulation-based classification	Categorical	1: 33.67%<br>0: 66.33%	0.00%

Note: SD: standard deviation

Table E.18: Descriptive statistics for the Diabetic Retinopathy dataset.

E.19 Hot Flashes

The Hot Flashes is a survey dataset that was collected from 373 patients with early breast cancer to understand the frequency and severity of vasomotor symptoms (VMS) and the effectiveness of previously applied interventions between June 5, 2020 and March 5, 2021 at two cancer in Ontario [7]. The outcome of interest is a binary variable, representing whether the severity of the VMS problem for a patient is high (coded as 1) or not (coded as 0). The dataset also collected a number of associated risk factors, including demographics, symptoms of having hot flashes, and medical treatments for hot flashes. A summary of the selected analysis variables is reported in Table E.19.

Variable	Description	Type	Mean (SD) or level count (% of total size)	Missingness (% of the total size)
Severity	Whether a patient had severe VMS (outcome)	Categorical	1: 49.44%<br>0: 50.56%	0.00%
Age	Age in years	Numeric	56.31 (10.52)	0.00%
Assessment of VMS	Whether a patient is asked about/assessed for symptoms of hot flashes by HCP during clinic visits	Categorical	1: 58.33%<br>0: 35.83%	5.83%
Hot flashes per week	The number of hot flashes in a week that occurred in the past week	Numeric	28.74 (47.00)	18.06%
Menopausal status	Current self-reported menopausal status	Categorical	1: 55.00%<br>0: 36.11%	8.89%
Feeling extremely hot/sweaty	Whether a patient had the symptom of feeling extremely hot/sweaty	Categorical	1: 76.67%<br>0: 23.33%	0.00%
Redness of my face/chest	Whether a patient had the symptom of redness on the face/chest	Categorical	1: 18.89%<br>0: 81.11%	0.00%
Feeling chills/clammy after hot flashes have passed	Whether a patient had the symptom of feeling chills/clammy after hot flashes passed	Categorical	1: 26.94%<br>0: 73.06%	0.00%
Waking up at night/difficulty sleeping	Whether a patient had the symptom of wake-up at night/having difficulty sleeping	Categorical	1: 55.56%<br>0: 44.44%	0.00%
Irritability	Whether a patient had irritability problems	Categorical	1: 12.22%<br>0: 87.78%	0.00%
Memory problems	Whether a patient had memory problems	Categorical	1: 10.83%<br>0: 89.17%	0.00%
Endocrine therapy	Endocrine therapy treatment for breast cancer	Categorical	1: 88.61%<br>0: 11.39%	0.00%
Ovarian function suppression	Ovarian function suppression treatment for breast cancer	Categorical	1: 19.44%<br>0: 80.56%	0.00%
Chemotherapy	Chemotherapy treatment for breast cancer	Categorical	1: 56.94%<br>0: 43.06%	0.00%
Change to breast cancer treatment	Changes made to breast cancer treatment due to hot flashes	Categorical	1: 18.33%<br>0: 81.67%	0.00%
Drug treatment for VMS	VMS treatments prescribed, recommended or tried by the patient	Categorical	1: 31.11%<br>0: 68.89%	0.00%
CAM therapies for VMS	Complementary treatments prescribed, recommended or tried by the patient	Categorical	1: 17.22%<br>0: 82.78%	0.00%

Referral to a menopause clinic	Patient referred or seen by a gynecologist or dedicated menopause clinic to assist in managing hot flashes	Categorical	1: 6.67%<br>0: 93.06%	0.28%

Note: SD: standard deviation

Table E.19: Descriptive statistics for the Hot Flashes dataset.

E.20 Thoracic Surgery

The Thoracic Surgery dataset contains the post-operative life expectancy of the patients who underwent major lung resections between 2007 and 2011. The dataset was collected retrospectively by Wroclaw Thoracic Surgery Centre, which is associated with the Department of Thoracic Surgery of the Medical University of Wroclaw and Lower-Silesian Centre for Pulmonary Diseases, Poland, while the research database constitutes a part of the National Lung Cancer Registry, administered by the Institute of Tuberculosis and Pulmonary Diseases in Warsaw, Poland [8]. The dataset has 470 observations. The outcome is a binary variable indicating whether the patient survived after 1 year period. The remaining sixteen variables are diagnosis code, patient's age, forced vital capacity, exhaled volume, performance status, pain, haemoptysis, dyspnoea, coughing, weakness, tumor size, diabetes mellitus, peripheral arterial diseases, smoking and asthma. The detailed variable characteristics are displayed in **Table E.20**.

Variable	Description	Type	Mean (SD) or level count (% of total size)	Missingness (% of the total size)
Outcome	Whether a patient died within one year after surgery	Categorical	1: 14.89%<br>0: 85.11%	0.00%
DGN	Diagnosis - specific combination of ICD-10 codes for primary and secondary as well multiple tumours if any	Categorical	DGN1: 0.21%<br>DGN2: 11.06%<br>DGN3: 74.26%<br>DGN4: 10.00%<br>DGN5: 3.19%<br>DGN6: 0.85%<br>DGN8: 0.43%	0.00%
AGE	Patient's age	Numeric	62.53 (8.71)	0.00%
PRE4	Forced vital capacity	Numeric	3.28 (0.87)	0.00%
PRE5	Volume that has been exhaled at the end of the first second of forced expiration	Numeric	4.57 (11.77)	0.00%
PRE6	Performance status - Zubrod scale	Categorical	PRZ0: 27.66%<br>PRZ1: 66.60%<br>PRZ2: 5.74%	0.00%
PRE7	Pain before surgery	Categorical	1: 6.60%<br>0: 93.40%	0.00%
PRE8	Haemoptysis before surgery	Categorical	1: 14.47%<br>0: 85.53%	0.00%
PRE9	Dyspnoea before surgery	Categorical	1: 6.60%<br>0: 93.40%	0.00%
PRE10	Cough before surgery	Categorical	1: 68.72%<br>0: 31.28%	0.00%
PRE11	Weakness before surgery	Categorical	1: 16.60%<br>0: 83.40%	0.00%
PRE14	T in clinical TNM - size of the original tumour, from OC11 (smallest) to OC14 (largest)	Categorical	OC11: 37.66%<br>OC12: 54.68%<br>OC13: 4.04%<br>OC14: 3.62%	0.00%
PRE17	Type 2 DM - diabetes mellitus	Categorical	1: 7.45%<br>0: 92.55%	0.00%
PRE19	MI up to 6 months	Categorical	1: 0.43%<br>0: 99.57%	0.00%
PRE25	PAD - peripheral arterial diseases	Categorical	1: 1.70%<br>0: 98.30%	0.00%
PRE30	Smoking	Categorical	1: 82.13%<br>0: 17.87%	0.00%
PRE32	Asthma	Categorical	1: 0.43%<br>0: 99.57%	0.00%

Note: SD: standard deviation

Table E.20: Descriptive statistics for the Thoracic Surgery dataset.

F References

1 Zwitter M, Soklic M. Breast Cancer. 1988.

2 Hond AAH de, Steyerberg EW, Calster B van. Interpreting area under the receiver operating characteristic curve. *The Lancet Digital Health*. 2022;4:e853–5. doi: 10.1016/S2589-7500(22)00188-1

3 Patrcio M, Pereira J, Crisstomo J, *et al.* Breast Cancer Coimbra. 2018.

4 Fernandes K, Cardoso J, Fernandes J. Quality Assessment of Digital Colposcopies. 2017.

5 Ingeholm P, Gögenur I, Iversen LH. Danish Colorectal Cancer Group Database. *Clin Epidemiol*. 2016;8:465–8. doi: 10.2147/CLEP.S99481

6 Antal B, Hajdu A. Diabetic Retinopathy Debrecen. 2014.

7 Cole KM, Clemons M, Alzahrani M, *et al.* Vasomotor symptoms in early breast cancer—a “real world” exploration of the patient experience. *Support Care Cancer*. 2022;30:4437–46. doi: 10.1007/s00520-022-06848-3

8 Lubicz M, Pawelczyk K, Rzechonek A, *et al.* Thoracic Surgery Data.

9 Azizi Z, Lindner S, Shiba Y, *et al.* A comparison of synthetic data generation and federated analysis for enabling international evaluations of cardiovascular health. *Sci Rep*. 2023;13:11540. doi: 10.1038/s41598-023-38457-3

10 Lloyd-Jones DM, Hong Y, Labarthe D, *et al.* Defining and Setting National Goals for Cardiovascular Health Promotion and Disease Reduction. *Circulation*. 2010;121:586–613. doi: 10.1161/CIRCULATIONAHA.109.192703

11 Maclagan LC, Park J, Sanmartin C, *et al.* The CANHEART health index: a tool for monitoring the cardiovascular health of the Canadian population. *CMAJ*. 2014;186:180–7. doi: 10.1503/cmaj.131358

12 Johnson AEW, Pollard TJ, Shen L, *et al.* MIMIC-III, a freely accessible critical care database. *Sci Data*. 2016;3:160035. doi: 10.1038/sdata.2016.35

13 Goldberger AL, Amaral LAN, Glass L, *et al.* PhysioBank, PhysioToolkit, and PhysioNet: Components of a New Research Resource for Complex Physiologic Signals. *Circulation*. 2000;101. doi: 10.1161/01.CIR.101.23.e215

14 Johnson A, Pollard T, Mark R. MIMIC-III Clinical Database (version 1.4). 2016.
